# Saproxylic beetles of the Po plain woodlands, Italy

**DOI:** 10.3897/BDJ.2.e1106

**Published:** 2014-07-22

**Authors:** Silvia Stefanelli, Francesca Della Rocca, Giuseppe Bogliani

**Affiliations:** † Department of Earth and Environmental Sciences, University of Pavia, Pavia, Italy

**Keywords:** Dead wood, saproxylic beetles, species checklist

## Abstract

Forest ecosystems play an important role for the conservation of biodiversity, and for the protection of ecological processes. The Po plain woodlands which once covered the whole Plain, today are reduced in isolated highly threatened remnants by modern intensive agriculture. These close to natural floodplain forests are one of the most scarce and endangered ecosystems in Europe.

Saproxylic species represent a major part of biodiversity of woodlands. The saproxylic insects are considered one of the most reliable bio-indicators of high-quality mature woodlands and have a very important role in regard to the protection and monitoring of forest biodiversity due to their highly specific living environments. As a result of the dramatic reduction of mature forests and the decreased availability of deadwood most of the saproxylic communities are greatly diminishing.

The study was conducted in the Ticino Valley Regional Park and the aim is to contribute to the expansion of knowledge on the saproxylic beetles of Lombardy. We investigated 6 sampling sites belonging to alluvial and riparian mixed forests. For each forest we selected 12 trees. For beetles’ collection we used two different traps: Eclector Traps and Trunk Window Traps (total of 72 traps and 864 samples collected).

We determined 4.387 beetles from 87 saproxylic species belonging to 21 families. Of these species 51 were not included in the previous checklist of the Park.

By comparing the two different techniques used for catching saproxylic beetles, we found a significantly high difference in species richness between Window Traps (WT) and Eclector Traps (ET) with a higher number of species captured in the Window Traps. However, the combined use of two different types of traps significantly expanded the spectrum of insects captured

Among the species reported as Least Concern in the IUCN Red List, we found interesting species such as the Elateridae *Calambus bipustulats*, the Eucnemidae *Melasis buprestoides* and the following species never previously found in the Park: Cerambycidae *Xylotrechus rusticus*, the Cetoniidae *Valgus hemipterus*, the Elateridae *Lacon punctatus*, the Mycetophagidae *Mycetophagus piceus*, *Litargus connexus*.

Although we didn’t find species listed in the Annexes of the EU Habitat Directive, some of the species found are locally threatened because of their rarity, local distribution, and strong linkage to old forests. Among these species there are the Bothrideridae *Bothrideres bipunctatus*, the Cerambycidae *Prionus coriarius* and *Xylotrechus rusticus*, the Dryophthoridae *Dryophthorus corticalis*, the Eucnemidae *Nematodes filum* (with only 1 individual captured in *Alnus* unmanged forest), the Histeridae *Aeletes atomarius* and *Paromalus flavicornis*, the Laemophloeidae *Cryptolestes duplicatus*, the Latridiidae *Enicmus rugosus* and *Latridius hirtus*, the Mycetophagidae *Mycetophagus piceus*, and the Zopheridae *Colydium elongatum* and *Pycnomerus terebrans*.

## Introduction

Forest ecosystems play an important role for the conservation of biodiversity and for the maintenance of ecological processes. Italian forests make up 34.7% of the country’s territory and are among the lushest in Europe (European Environment Agency 2006). North Italy, in particular the area of the Po Plain Valley, was once entirely covered by alluvial floodplain forests. Today these kinds of forests are reduced to small remnants, are considered one of most at risk ecosystems, and are strongly threatened by intensive agriculture and industrial activity (Minelli et al. 2002). Two examples of these ecosystems are the "Bosco Della Fontana" in Mantova and the "Ticino Valley Regional Park".

Saproxylic species represent roughly 20–30% of the invertebrate fauna of the European broad-leaved forests (Wermelinger et al. 2002, Vallauri et al. 2005) and constitute a huge part of woodland biodiversity. Moreover, saproxylic insects are considered one of the most reliable bio-indicators of high-quality mature woodlands (Speight 1989, Fowles et al. 1999, Brustel 2001, Alexander 2004, Johnsson et al. 2005) and have a very important role in regard to the protection and monitoring of forest biodiversity due to their highly specific living environments (Schlaghamerský 2000). The role of insects in the decomposition of deadwood and in the distribution of woody debris is essential (Schlaghamerský 2003, Harmon et al. 1986), and their conservation is strongly needed in order to preserve overall biodiversity.

As a result of the dramatic reduction in mature forests and the decreased availability of deadwood, most saproxylic communities are diminishing greatly. Several species are suffering from fragmented distribution, and in some cases, are disappearing from their former strongholds (Geiser 1998).

The ecology and distribution of many saproxyilic species in Mediterranean countries are poorly studied or completely unknown (Buse et al. 2010). Data on the saproxylic fauna of lowland forests, particularly on floodplains, is very limited (Nieto and Alexander 2010). The aim of this study is to expand our knowledge about the Italian saproxylic beetle populations and, more specifically, to gain more information about the species present in the floodplain forests of the Po Valley along a gradient of deadwood consumption within the Ticino Valley Natural Park.

## Materials and methods

### Study area

The study was conducted in the Ticino Valley Natural Park located in Northwest Italy 30 km south of the city of Milan. The park, which was acknowledged as the MAB Biosphere Reserve “Valle del Ticino” (UNESCO 2005), covers an area of 287 km^2^ along the banks of the Ticino River from Lake Maggiore to its confluence with the Po River. This particular geographic position crosses the most urbanized area of the country and represents an important ecological corridor between the Alps and the Apennines by creating a biological connection between continental Europe and the Mediterranean area. The Ticino Valley represents an area of high biodiversity with a large variety of habitats: conifer forests, lowland forests, waterways, wetlands, cultivated fields, heathlands, and meadows (Bogliani and Furlanetto 1995). In particular, the valley forests represent what remains of the ancient lowland deciduous forests that once covered the plains of northern Italy. Woodlands cover 195.46 km^2^ or 60% of the whole natural park and mainly consist of the two habitat types listed in Annex I of the EU Habitat Directive 92/43/CEE (Falco et al. 2008): 1) 91E0* – Alluvial forests with *Alnus glutinosa* and *Fraxinus excelsior* (Alno-Pandion, Alnion incanae, Salicion albae) and 2) 91F0* – Riparian mixed forests of *Quercus robur*, *Ulmus laevis*, and *Ulmus minor*.

**Figure 1.**
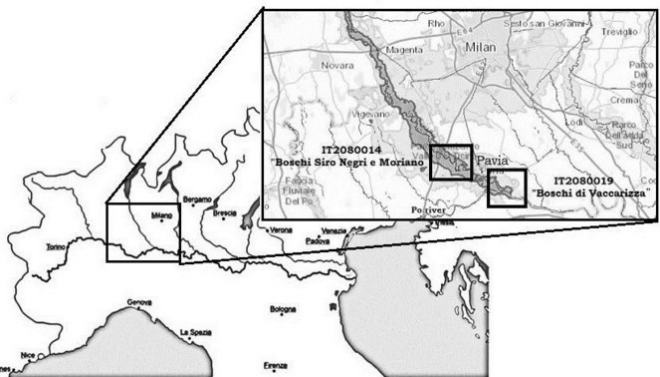
The Southern side of the Ticino Valley Natural Park.

This study covers two Sites of Community Importance (SCI): “Boschi Siro Negri e Moriano” (IT2080014) and “Boschi di Vaccarizza” (IT2080019) located on the southern side of the Park (Fig. 1)

The SCI “Boschi Siro Negri e Moriano” extends along the Ticino River from the bridge of the Milano-Genova highway to the city of Pavia and covers an area of 13.52 km^2^. The SCI has an elongated shape and is inserted inside a broad valley groove with a low slope that widens as you move from North to South. The river is included in a dense hydrographic network represented by irrigation ditches that constitute an interesting environmental and wetland habitat with a high natural value (Perracino 2010). The forest vegetation includes both habitat types described above with the most common trees being the pedunculated oak (*Quercus robur*), three species of poplars (*Populus alba*, *Populus nigra*, *Populus tremulus*), and the locust tree (*Robinia pseudoacacia*) and the most common high shrubs being hazel (*Corylus avellana*), hawthorn (*Crataegus monogyna*), and bird cherry (*Prunus padus*). The most interesting area in the SCI is the Integral Nature Reserve "Bosco Siro Negri" (BN10). The Reserve, established in 1973, is characterized by the presence of unmanaged lowland forests that represent the vegetation which covered a large part of the Po Valley before Roman expansion (Tomaselli and Gentile 1971).

The SCI “Boschi di Vaccarizza” covers an area of 4.65 km^2^ between the city of Pavia and the Southern border of the park; it is completely included inside the Ticino Valley Regional Park, but it’s located in an area subjected to deep transformations by human activities. The SCI is located downstream of the confluence of the Ticino River into Po River. In this stretch, the floodplain is very large when compared to many other waterways in the area and is occupied, for the most part, by poplar plantations which characterize the cultivation of the area. Only in the Northern part of the SCI and in the ranges in close contact with the river did we find a more complex vegetation structure (Perracino 2010). The study area includes only the northern part of the SCI and sampling was only carried out in one type of habitat: the wet forests belonging to type "*91E0: Alluvional forests" (Falco et al. 2008) which featured a prevalence of alder trees, *Alnus glutinosa*.

### Study design

We investigated 6 sampling sites consisting of both managed and unmanaged forests. We considered unmanaged forests those which had not been influenced by direct human disturbance for at least 20 years (Paillet et al. 2010). We analyzed data from 2 riparian mixed forests (habitat code 91F0*) dominated by *Quercus robur* (called here in this paper "*Quercus* forests") and 4 Alluvial forests (habitat code 91E0*). The Alluvial forests included 2 riparian forests of *Alnus glutinosa* (Corine Biotope code 44.3 and called in this paper “*Alnus* forests”) and 2 arborescent galleries of tall *Salix alba*, *Salix fragilis*, and *Populus nigra* (Corine biotope 44.13: called in this paper “*Populus* forests”).

In the SCI "Boschi Siro Negri e Moriano", we considered two *Populus* forests consisting of 1 managed site (BN1) and 1 unmanaged site (BN21). We also analyzed two *Quercus* forests consisting of 1 managed (BN5) and 1 unmanaged site (BN10).

In the SCI "Boschi di Vaccarizza", we investigated two *Alnus* forests. In this case we also investigated 1 unmanged forest (V1) and 1 managed forest (V2).

For each forest, we selected and georeferenced 12 trees, 6 fallen and 6 standing, belonging to three decay classes of wood according to the criteria described in the manual "BioSoil - Biodiversity Project" (Cindolo and Petriccione 2006): Class 1 – hard and compact wood with intact bark, Class 2 – hardwood or initial disintegration (penetrable up to 1cm) with bark partially absent, and Class 3 – soft wood (penetrable up to 3 cm or more) with bark almost totally absent. We placed traps on two trees for each decaying class.

**Figure 2.**
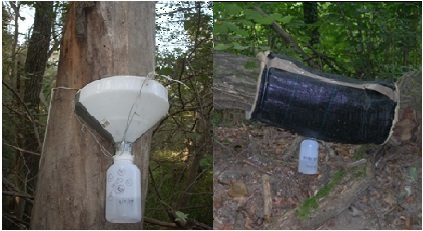
Trunk Window Traps (Kaila 1993) and Eclector Traps (Alinvi et al. 2007).

For collecting beetles, we used two different traps: 1) Eclector Traps (Alinvi et al. 2007) used for the quantitative sampling of insects emerging from logs in a moderately advanced stage of decomposition and 2) Trunk Window Traps (Kaila 1993) used to sample the total assemblage of flying saproxylic beetles (Fig. 2).

All traps were checked every two weeks from April 2010 to September 2010 for a total of 12 collections from each forest and 864 samples collected during the study. Of the 68 families identified, 48 were found to be saproxylic and we considered obligate saproxyilics to be those beetles which depend on dead wood in at least part of their lifecycle (Gibb et al. 2006). Among the 48 saproxilic families, we choose only 21 families on the basis of there being a significant number of saproxylic species in the family and the availability of a specialist to identify the specimens.

The families of beetles determined to the species level were: Anthribidae (Billberg, 1820), Bothrideridae (Erichson, 1845), Cerambycidae (Latreille, 1802), Cerylonidae (Billberg, 1820), Cetoniidae (Leach, 1815), Curculionidae (Latreille, 1802), Dryophthoridae (Schönherr, 1825), Elateridae (Leach, 1815), Erotylidae (Latreille, 1802), Eucnemidae (Eschscholtz, 1829), Histeridae (Gyllenhal, 1808), Laemophloeidae (Ganglbauer, 1899), Latridiidae (Erichson, 1842), Lissomidae (Castelnau, 1840), Lucanidae (Latreille, 1804), Mycetophagidae (Leach, 1815), Monotomidae (Laporte, 1840), Nitidulidae (Latreille, 1802), Silvanidae (Kirby, 1837), Tenebrionidae (Latreille, 1802), and Zopheridae (Solier, 1834).

### Data analysis

In the analysis, we used the pooled sample of 72 traps (36 windows traps and 36 eclector traps). As a measure of species richness (α-diversity) (Whittaker 1972) we used the number of species caught in each plot because the sampling effort was the same at all the sites. The number of species was log-transformed to approach a normal distribution. We compared species richness among the tree habitat types using a one-way ANOVA and between managed and unmanaged forests using a T-test.

## Checklists

### Saproxylic beetles of the Ticino Valley Regional Park

#### Anthribus nebulosus

(Forster, 1770)

Brachytarsus nebulosus Kuster, 1859 – Fauna Europaea (2013)
Bruchus varius Fabricius, 1787 – Fauna Europaea (2013)
Bruchus clathratus Herbst, 1786 – Fauna Europaea (2013)
Anthribus variegatus Geoffroy, 1785 – Fauna Europaea (2013)

##### Materials

- **Type status:** Other material. **Occurrence:** recordedBy: Silvia Stefanelli; individualCount: 1; lifeStage: adult; **Taxon:** taxonID: urn:lsid:faunaeur.org:taxname:186820; scientificName: Anthribus nebulosus; order: Coleoptera; family: Anthribidae; genus: Anthribus; scientificNameAuthorship: Forster 1770; **Location:** country: Italy; stateProvince: Pavia; locality: SIC “Boschi Siro Negri e Moriano" - BN5; verbatimElevation: 69 m; verbatimCoordinates: 32T 502886E 5008393N; verbatimCoordinateSystem: UTM WGS 84; decimalLatitude: 45.229029; decimalLongitude: 9.036770; georeferencedBy: Silvia Stefanelli; georeferenceProtocol: GPS; **Identification:** identifiedBy: Paolo Cornacchia; dateIdentified: 2011

##### Distribution

Austria, Belarus, Belgium, Bosnia and Herzegovina, Britain I., Bulgaria, Corsica, Czech Republic, Danish mainland, Estonia, Finland, French mainland, Germany, Greek mainland, Hungary, Italian mainland, Latvia, Lithuania, Luxembourg, Norwegian mainland, Poland, Portuguese mainland, Romania, Russia North, Sardinia, Slovakia, Spanish mainland, Sweden, Switzerland, The Netherlands, Ukraine, Yugoslavia, East Palaearctic, Near East (Asian Turkey, Caucasian Russian republics, Georgia, Armenia, Azerbaidjan, Lebanon, Syria, Israel, Jordan, Sinai Peninsula (Egypt), Arabian peninsula, Iran, Iraq) (Fauna Europaea 2013).

##### Notes

The species lives in conifer, broadleaves, mixed, and floodplain forests. The larva is a predator of Coccidae and develops mainly on pine and fir, while the adult is found on larch, willow, oak, and beech (Hoebeke and Wheeler 1991, Holuša and Trýzna 2007).

#### Eusphyrus vasconicus

(Hoffmann, 1954)

##### Materials

- **Type status:** Other material. **Occurrence:** recordedBy: Silvia Stefanelli; individualCount: 12; lifeStage: adult; **Taxon:** taxonID: urn:lsid:faunaeur.org:taxname:186799; scientificName: Eusphyrus vasconicus; order: Coleoptera; family: Anthribidae; genus: Eusphyrus; scientificNameAuthorship: Hoffmann 1954; **Location:** country: Italy; stateProvince: Pavia; locality: SIC “Boschi di Vaccarizza" - V1; verbatimElevation: 62 m; verbatimCoordinates: 32T 519272E 4999526N; verbatimCoordinateSystem: UTM WGS 84; decimalLatitude: 45.148947; decimalLongitude: 9.245157; georeferencedBy: Silvia Stefanelli; georeferenceProtocol: GPS; **Identification:** identifiedBy: Paolo Cornacchia; dateIdentified: 2011
- **Type status:** Other material. **Occurrence:** recordedBy: Silvia Stefanelli; individualCount: 5; lifeStage: adult; **Taxon:** taxonID: urn:lsid:faunaeur.org:taxname:186799; scientificName: Eusphyrus vasconicus; order: Coleoptera; family: Anthribidae; genus: Eusphyrus; scientificNameAuthorship: Hoffmann 1954; **Location:** country: Italy; stateProvince: Pavia; locality: SIC “Boschi di Vaccarizza" - V2; verbatimElevation: 65 m; verbatimCoordinates: 32T 519868E 4999488N; verbatimCoordinateSystem: UTM WGS 84; decimalLatitude: 45.148589; decimalLongitude: 9.252737; georeferencedBy: Silvia Stefanelli; georeferenceProtocol: GPS; **Identification:** identifiedBy: Paolo Cornacchia; dateIdentified: 2011

##### Distribution

French mainland and Italian mainland (Fauna Europaea 2013).

#### Phaenotherion fasciculatum

(Reitter, 1891)

##### Materials

- **Type status:** Other material. **Occurrence:** recordedBy: Silvia Stefanelli; individualCount: 2; lifeStage: adult; **Taxon:** taxonID: urn:lsid:faunaeur.org:taxname:186775; scientificName: Phaenotherion fasciculatum; order: Coleoptera; family: Anthribidae; genus: Phaenotherion; scientificNameAuthorship: Reitter 1891; **Location:** country: Italy; stateProvince: Pavia; locality: SIC "Boschi Siro Negri e Moriano" - BN10; verbatimElevation: 76 m; verbatimCoordinates: 32T 504479E 5006332N; verbatimCoordinateSystem: UTM WGS 84; decimalLatitude: 45.210461; decimalLongitude: 9.057038; georeferencedBy: Silvia Stefanelli; georeferenceProtocol: GPS; **Identification:** identifiedBy: Paolo Cornacchia; dateIdentified: 2011

##### Distribution

Croatia, Italian mainland (Fauna Europaea 2013).

##### Notes

The species is polyphagous. The larva develops and lives in the dead branches of different wood species; the adult is sometimes found in soil and roots (Abbazzi et al. 1999).

#### Platystomos albinus

(Linneaus, 1758)

##### Materials

- **Type status:** Other material. **Occurrence:** recordedBy: Silvia Stefanelli; individualCount: 1; lifeStage: adult; **Taxon:** taxonID: urn:lsid:faunaeur.org:taxname:186761; scientificName: Platystomos albinus; order: Coleoptera; family: Anthribidae; genus: Platystomos; scientificNameAuthorship: Linnaeus 1758; **Location:** country: Italy; stateProvince: Pavia; locality: SIC "Boschi Siro Negri e Moriano" - BN1; verbatimElevation: 68 m; verbatimCoordinates: 32T 503258E 5007870N; verbatimCoordinateSystem: UTM WGS 84; decimalLatitude: 45.224312; decimalLongitude: 9.041499; georeferencedBy: Silvia Stefanelli; georeferenceProtocol: GPS; **Identification:** identifiedBy: Paolo Cornacchia; dateIdentified: 2011
- **Type status:** Other material. **Occurrence:** recordedBy: Silvia Stefanelli; individualCount: 1; lifeStage: adult; **Taxon:** taxonID: urn:lsid:faunaeur.org:taxname:186761; scientificName: Platystomos albinus; order: Coleoptera; family: Anthribidae; genus: Platystomos; scientificNameAuthorship: Linnaeus 1758; **Location:** country: Italy; stateProvince: Pavia; locality: SIC "Boschi Siro Negri e Moriano" - BN5; verbatimElevation: 62 m; verbatimCoordinates: 32T 502886E 5008393N; verbatimCoordinateSystem: UTM WGS 84; decimalLatitude: 45.229029; decimalLongitude: 9.036770; georeferencedBy: Silvia Stefanelli; georeferenceProtocol: GPS; **Identification:** identifiedBy: Paolo Cornacchia; dateIdentified: 2011
- **Type status:** Other material. **Occurrence:** recordedBy: Silvia Stefanelli; individualCount: 3; lifeStage: adult; **Taxon:** taxonID: urn:lsid:faunaeur.org:taxname:186761; scientificName: Platystomos albinus; order: Coleoptera; family: Anthribidae; genus: Platystomos; scientificNameAuthorship: Linnaeus 1758; **Location:** country: Italy; stateProvince: Pavia; locality: SIC "Boschi Siro Negri e Moriano" - BN10; verbatimElevation: 76 m; verbatimCoordinates: 32T 504479E 5006332N; verbatimCoordinateSystem: UTM WGS 84; decimalLatitude: 45.210461; decimalLongitude: 9.057038; georeferencedBy: Silvia Stefanelli; georeferenceProtocol: GPS; **Identification:** identifiedBy: Paolo Cornacchia; dateIdentified: 2011
- **Type status:** Other material. **Occurrence:** recordedBy: Silvia Stefanelli; individualCount: 3; lifeStage: adult; **Taxon:** taxonID: urn:lsid:faunaeur.org:taxname:186761; scientificName: Platystomos albinus; order: Coleoptera; family: Anthribidae; genus: Platystomos; scientificNameAuthorship: Linnaeus 1758; **Location:** country: Italy; stateProvince: Pavia; locality: SIC "Boschi di Vaccarizza" - V1; verbatimElevation: 62 m; verbatimCoordinates: 32T 519272E 4999526N; verbatimCoordinateSystem: UTM WGS 84; decimalLatitude: 45.148947; decimalLongitude: 9.245157; georeferencedBy: Silvia Stefanelli; georeferenceProtocol: GPS; **Identification:** identifiedBy: Paolo Cornacchia; dateIdentified: 2011
- **Type status:** Other material. **Occurrence:** recordedBy: Silvia Stefanelli; individualCount: 2; lifeStage: adult; **Taxon:** taxonID: urn:lsid:faunaeur.org:taxname:186761; scientificName: Platystomos albinus; order: Coleoptera; family: Anthribidae; genus: Platystomos; scientificNameAuthorship: Linnaeus 1758; **Location:** country: Italy; stateProvince: Pavia; locality: SIC "Boschi di Vaccarizza" - V2; verbatimElevation: 65 m; verbatimCoordinates: 32T 519868E 4999488N; verbatimCoordinateSystem: UTM WGS 84; decimalLatitude: 45.148589; decimalLongitude: 9.252737; georeferencedBy: Silvia Stefanelli; georeferenceProtocol: GPS; **Identification:** identifiedBy: Paolo Cornacchia; dateIdentified: 2011

##### Distribution

Albania, Austria, Belarus, Belgium, Bosnia and Herzegovina, Britain I., Bulgaria, Croatia, Czech Republic, Danish mainland, Estonia, Finland, French mainland, Germany, Hungary, Italian mainland, Latvia, Lithuania, Luxembourg, Macedonia, Norwegian mainland, Poland, Romania, Russia North, Sardinia, Slovakia, Slovenia, Sweden, Switzerland, The Netherlands, Ukraine, Yugoslavia, East Palaearctic, Near East (Asian Turkey, Caucasian Russian republics, Georgia, Armenia, Azerbaidjan, Lebanon, Syria, Israel, Jordan, Sinai Peninsula (Egypt), Arabian peninsula, Iran, Iraq) (Fauna Europaea 2013).

##### Notes

The larva develops in dead and dying trees usually in the forest; it is often associated with the fungus *Daldinia* sp. (Alexander 2002).

#### Bothrideres bipunctatus

(Gmelin, 1790)

##### Materials

- **Type status:** Other material. **Occurrence:** recordedBy: Silvia Stefanelli; individualCount: 11; lifeStage: adult; **Taxon:** taxonID: urn:lsid:faunaeur.org:taxname:101122; scientificName: Bothrideres bipunctatus; order: Coleoptera; family: Bothrideridae; genus: Bothrideres; scientificNameAuthorship: Gmelin 1790; **Location:** country: Italy; stateProvince: Pavia; locality: SIC "Boschi Siro Negri e Moriano" - BN1; verbatimElevation: 68 m; verbatimCoordinates: 32T 503258E 5007870N; verbatimCoordinateSystem: UTM WGS 84; decimalLatitude: 45.224312; decimalLongitude: 9.041499; georeferencedBy: Silvia Stefanelli; georeferenceProtocol: GPS; **Identification:** identifiedBy: Paolo Cornacchia; dateIdentified: 2011
- **Type status:** Other material. **Occurrence:** recordedBy: Silvia Stefanelli; individualCount: 2; lifeStage: adult; **Taxon:** taxonID: urn:lsid:faunaeur.org:taxname:101122; scientificName: Bothrideres bipunctatus; order: Coleoptera; family: Bothrideridae; genus: Bothrideres; scientificNameAuthorship: Gmelin 1790; **Location:** country: Italy; stateProvince: Pavia; locality: SIC "Boschi Siro Negri e Moriano" - BN21; verbatimElevation: 66 m; verbatimCoordinates: 32T 506342E 5005026N; verbatimCoordinateSystem: UTM WGS 84; decimalLatitude: 45.198691; decimalLongitude: 9.080746; georeferencedBy: Silvia Stefanelli; georeferenceProtocol: GPS; **Identification:** identifiedBy: Paolo Cornacchia; dateIdentified: 2011
- **Type status:** Other material. **Occurrence:** recordedBy: Silvia Stefanelli; individualCount: 4; lifeStage: adult; **Taxon:** taxonID: urn:lsid:faunaeur.org:taxname:101122; scientificName: Bothrideres bipunctatus; order: Coleoptera; family: Bothrideridae; genus: Bothrideres; scientificNameAuthorship: Gmelin 1790; **Location:** country: Italy; stateProvince: Pavia; locality: SIC "Boschi Siro Negri e Moriano" - BN5; verbatimElevation: 62 m; verbatimCoordinates: 32T 502886E 5008393N; verbatimCoordinateSystem: UTM WGS 84; decimalLatitude: 45.229029; decimalLongitude: 9.036770; georeferencedBy: Silvia Stefanelli; georeferenceProtocol: GPS; **Identification:** identifiedBy: Paolo Cornacchia; dateIdentified: 2011
- **Type status:** Other material. **Occurrence:** recordedBy: Silvia Stefanelli; individualCount: 6; lifeStage: adult; **Taxon:** taxonID: urn:lsid:faunaeur.org:taxname:101122; scientificName: Bothrideres bipunctatus; order: Coleoptera; family: Bothrideridae; genus: Bothrideres; scientificNameAuthorship: Gmelin 1790; **Location:** country: Italy; stateProvince: Pavia; locality: SIC "Boschi Siro Negri e Moriano" - BN10; verbatimElevation: 76 m; verbatimCoordinates: 32T 504479E 5006332N; verbatimCoordinateSystem: UTM WGS 84; decimalLatitude: 45.210461; decimalLongitude: 9.057038; georeferencedBy: Silvia Stefanelli; georeferenceProtocol: GPS; **Identification:** identifiedBy: Paolo Cornacchia; dateIdentified: 2011
- **Type status:** Other material. **Occurrence:** recordedBy: Silvia Stefanelli; individualCount: 3; lifeStage: adult; **Taxon:** taxonID: urn:lsid:faunaeur.org:taxname:101122; scientificName: Bothrideres bipunctatus; order: Coleoptera; family: Bothrideridae; genus: Bothrideres; scientificNameAuthorship: Gmelin 1790; **Location:** country: Italy; stateProvince: Pavia; locality: SIC "Boschi di Vaccarizza" - V1; verbatimElevation: 62 m; verbatimCoordinates: 32T 519272E 4999526N; verbatimCoordinateSystem: UTM WGS 84; decimalLatitude: 45.148947; decimalLongitude: 9.245157; georeferencedBy: Silvia Stefanelli; georeferenceProtocol: GPS; **Identification:** identifiedBy: Paolo Cornacchia; dateIdentified: 2011
- **Type status:** Other material. **Occurrence:** recordedBy: Silvia Stefanelli; individualCount: 2; lifeStage: adult; **Taxon:** taxonID: urn:lsid:faunaeur.org:taxname:101122; scientificName: Bothrideres bipunctatus; order: Coleoptera; family: Bothrideridae; genus: Bothrideres; scientificNameAuthorship: Gmelin 1790; **Location:** country: Italy; stateProvince: Pavia; locality: SIC "Boschi di Vaccarizza" - V2; verbatimElevation: 65 m; verbatimCoordinates: 32T 519868E 4999488N; verbatimCoordinateSystem: UTM WGS 84; decimalLatitude: 45.148589; decimalLongitude: 9.252737; georeferencedBy: Silvia Stefanelli; georeferenceProtocol: GPS; **Identification:** identifiedBy: Paolo Cornacchia; dateIdentified: 2011

##### Distribution

Austria, Azores, Balearic Is., Belarus, Belgium, Bosnia and Herzegovina, Britain I., Bulgaria, Canary Is., Channel Is., Corsica, Crete, Croatia, Cyclades Is., Cyprus, Czech Republic, Danish mainland, Dodecanese Is., Estonia, European Turkey, Faroe Is., Finland, Franz Josef Land, French mainland, Germany, Gibraltar, Greek mainland, Hungary, Iceland, Ireland, Italian mainland, Kaliningrad Region, Latvia, Liechtenstein, Lithuania, Luxembourg, Macedonia, Madeira, Malta, Moldova Republic of, Monaco, North Aegean Is., Northern Ireland, Norwegian mainland, Novaya Zemlya, Poland, Portuguese mainland, Romania, Russia Central, Russia East, Russia North, Russia Northwest, Russia South, San Marino, Sardinia, Selvagens Is., Sicily, Slovakia, Slovenia, Spanish mainland, Svalbard & Jan Mayen, Sweden, Switzerland, The Netherlands, Ukraine, Vatican City, Yugoslavia (Fauna Europaea 2013).

##### Notes

The species lives in relict virgin forests (Bussler et al. 2005). It is found in the larval galleries of other beetles in the decaying woods of broadleaves, mainly willow and poplar. The larva is polymetabolous ectoparasitoids of the longhorn and jewel beetle larvae (Hůrka 2005).

#### Oxylaemus cylindricus

(Panzer, 1796)

##### Materials

- **Type status:** Other material. **Occurrence:** recordedBy: Silvia Stefanelli; individualCount: 2; lifeStage: adult; **Taxon:** taxonID: urn:lsid:faunaeur.org:taxname:101233; scientificName: Oxylaemus cylindricus; order: Coleoptera; family: Bothrideridae; genus: Oxylaemus; scientificNameAuthorship: Panzer 1796; **Location:** country: Italy; stateProvince: Pavia; locality: SIC "Boschi Siro Negri e Moriano" - BN5; verbatimElevation: 62 m; verbatimCoordinates: 32T 502886E 5008393N; verbatimCoordinateSystem: UTM WGS 84; decimalLatitude: 45.229029; decimalLongitude: 9.036770; georeferencedBy: Silvia Stefanelli; georeferenceProtocol: GPS; **Identification:** identifiedBy: Paolo Cornacchia; dateIdentified: 2011
- **Type status:** Other material. **Occurrence:** recordedBy: Silvia Stefanelli; individualCount: 9; lifeStage: adult; **Taxon:** taxonID: urn:lsid:faunaeur.org:taxname:101233; scientificName: Oxylaemus cylindricus; order: Coleoptera; family: Bothrideridae; genus: Oxylaemus; scientificNameAuthorship: Panzer 1796; **Location:** country: Italy; stateProvince: Pavia; locality: SIC "Boschi Siro Negri e Moriano" - BN10; verbatimElevation: 76 m; verbatimCoordinates: 32T 504479E 5006332N; verbatimCoordinateSystem: UTM WGS 84; decimalLatitude: 45.210461; decimalLongitude: 9.057038; georeferencedBy: Silvia Stefanelli; georeferenceProtocol: GPS; **Identification:** identifiedBy: Paolo Cornacchia; dateIdentified: 2011

##### Distribution

Austria, Azores, Balearic Is., Belarus, Belgium, Bosnia and Herzegovina, Britain I., Bulgaria, Canary Is., Channel Is., Corsica, Crete, Croatia, Cyclades Is., Cyprus, Czech Republic, Danish mainland, Dodecanese Is., Estonia, European Turkey, Faroe Is., Finland, Franz Josef Land, French mainland, Germany, Gibraltar, Greek mainland, Hungary, Iceland, Ireland, Italian mainland, Kaliningrad Region, Latvia, Liechtenstein, Lithuania, Luxembourg, Macedonia, Madeira, Malta, Moldova, Republic of Monaco, North Aegean Is., Northern Ireland, Norwegian mainland, Novaya Zemlya, Poland, Portuguese mainland, Romania, Russia Central, Russia East, Russia North, Russia Northwest, Russia South, San Marino, Sardinia, Selvagens Is., Sicily, Slovakia, Slovenia, Spanish mainland, Svalbard & Jan Mayen, Sweden, Switzerland, The Netherlands, Ukraine, Vatican City, Yugoslavia (Fauna Europaea 2013).

##### Notes

The species lives infrequently in the bark of old oaks as a commensal in the galleries of the bark beetles which grow ambrosia fungi (Hůrka 2005).

#### Aegomorphus clavipes

(Schrank, 1781)

Cerambyx varius Fabricius, 1787 – Fauna Europaea (2013)

##### Materials

- **Type status:** Other material. **Occurrence:** recordedBy: Silvia Stefanelli; individualCount: 5; lifeStage: adult; **Taxon:** taxonID: urn:lsid:faunaeur.org:taxname:114017; scientificName: Aegomorphus clavipes; order: Coleoptera; family: Cerambycidae; genus: Aegomorphus; scientificNameAuthorship: Schrank 1781; **Location:** country: Italy; stateProvince: Pavia; locality: SIC "Boschi Siro Negri e Moriano" - BN21; verbatimElevation: 66 m; verbatimCoordinates: 32T 506342E 5005026N; verbatimCoordinateSystem: UTM WGS 84; decimalLatitude: 45.198691; decimalLongitude: 9.080746; georeferenceProtocol: GPS; **Identification:** identifiedBy: Carlo Pesarini; dateIdentified: 2011

##### Distribution

Albania, Andorra, Austria, Azores, Balearic Is., Belarus, Belgium, Bosnia and Herzegovina, Britain I., Bulgaria, Canary Is., Channel Is., Corsica, Crete, Croatia, Cyclades Is., Cyprus, Czech Republic, Danish mainland, Dodecanese Is., Estonia, European Turkey, Faroe Is., Finland, Franz Josef Land, French mainland, Germany, Gibraltar, Greek mainland, Hungary, Iceland, Ireland, Italian mainland, Kaliningrad Region, Latvia, Liechtenstein, Lithuania, Luxembourg, Macedonia, Madeira, Malta, Moldova Republic of, Monaco, North Aegean Is., Northern Ireland, Norwegian mainland, Novaya Zemlya, Poland, Portuguese mainland, Romania, Russia Central, Russia East, Russia North, Russia Northwest, Russia South, San Marino, Sardinia, Selvagens Is., Sicily, Slovakia, Slovenia, Spanish mainland, Svalbard & Jan Mayen, Sweden, Switzerland, The Netherlands, Ukraine, Vatican City, Yugoslavia, Afro-tropical region, Australian region, East Palaearctic, Near East, Nearctic region, Neotropical region, North Africa (Fauna Europaea 2013).

#### Aegosoma scabricorne

(Scopoli, 1763)

##### Materials

- **Type status:** Other material. **Occurrence:** recordedBy: Silvia Stefanelli; individualCount: 7; lifeStage: adult; **Taxon:** taxonID: urn:lsid:faunaeur.org:taxname:115112; scientificName: Aegosoma scabricorne; order: Coleoptera; family: Cerambycidae; genus: Aegosoma; scientificNameAuthorship: Scopoli 1763; **Location:** country: Italy; stateProvince: Pavia; locality: SIC "Boschi Siro Negri e Moriano" - BN1; verbatimElevation: 68 m; verbatimCoordinates: 32T 503258E 5007870N; verbatimCoordinateSystem: UTM WGS 84; decimalLatitude: 45.224312; decimalLongitude: 9.041499; georeferencedBy: Silvia Stefanelli; georeferenceProtocol: GPS; **Identification:** identifiedBy: Carlo Pesarini; dateIdentified: 2011
- **Type status:** Other material. **Occurrence:** recordedBy: Silvia Stefanelli; individualCount: 1; lifeStage: adult; **Taxon:** taxonID: urn:lsid:faunaeur.org:taxname:115112; scientificName: Aegosoma scabricorne; order: Coleoptera; family: Cerambycidae; genus: Aegosoma; scientificNameAuthorship: Scopoli 1763; **Location:** country: Italy; stateProvince: Pavia; locality: SIC "Boschi Siro Negri e Moriano" - BN21; verbatimElevation: 66 m; verbatimCoordinates: 32T 506342E 5005026N; verbatimCoordinateSystem: UTM WGS 84; decimalLatitude: 45.198691; decimalLongitude: 9.080746; georeferencedBy: Silvia Stefanelli; georeferenceProtocol: GPS; **Identification:** identifiedBy: Carlo Pesarini; dateIdentified: 2011
- **Type status:** Other material. **Occurrence:** recordedBy: Silvia Stefanelli; individualCount: 3; lifeStage: adult; **Taxon:** taxonID: urn:lsid:faunaeur.org:taxname:115112; scientificName: Aegosoma scabricorne; order: Coleoptera; family: Cerambycidae; genus: Aegosoma; scientificNameAuthorship: Scopoli 1763; **Location:** country: Italy; stateProvince: Pavia; locality: SIC "Boschi Siro Negri e Moriano" - BN5; verbatimElevation: 62 m; verbatimCoordinates: 32T 502886E 5008393N; verbatimCoordinateSystem: UTM WGS 84; decimalLatitude: 45.229029; decimalLongitude: 9.036770; georeferencedBy: Silvia Stefanelli; georeferenceProtocol: GPS; **Identification:** identifiedBy: Carlo Pesarini; dateIdentified: 2011
- **Type status:** Other material. **Occurrence:** recordedBy: Silvia Stefanelli; individualCount: 7; lifeStage: adult; **Taxon:** taxonID: urn:lsid:faunaeur.org:taxname:115112; scientificName: Aegosoma scabricorne; order: Coleoptera; family: Cerambycidae; genus: Aegosoma; scientificNameAuthorship: Scopoli 1763; **Location:** country: Italy; stateProvince: Pavia; locality: SIC "Boschi Siro Negri e Moriano" - BN10; verbatimElevation: 76 m; verbatimCoordinates: 32T 504479E 5006332N; verbatimCoordinateSystem: UTM WGS 84; decimalLatitude: 45.210461; decimalLongitude: 9.057038; georeferencedBy: Silvia Stefanelli; georeferenceProtocol: GPS; **Identification:** identifiedBy: Carlo Pesarini; dateIdentified: 2011
- **Type status:** Other material. **Occurrence:** recordedBy: Silvia Stefanelli; individualCount: 3; lifeStage: adult; **Taxon:** taxonID: urn:lsid:faunaeur.org:taxname:115112; scientificName: Aegosoma scabricorne; order: Coleoptera; family: Cerambycidae; genus: Aegosoma; scientificNameAuthorship: Scopoli 1763; **Location:** country: Italy; stateProvince: Pavia; locality: SIC "Boschi di Vaccarizza" - V1; verbatimElevation: 62 m; verbatimCoordinates: 32T 519272E 4999526N; verbatimCoordinateSystem: UTM WGS 84; decimalLatitude: 45.148947; decimalLongitude: 9.245157; georeferencedBy: Silvia Stefanelli; georeferenceProtocol: GPS; **Identification:** identifiedBy: Carlo Pesarini; dateIdentified: 2011
- **Type status:** Other material. **Occurrence:** recordedBy: Silvia Stefanelli; individualCount: 4; lifeStage: adult; **Taxon:** taxonID: urn:lsid:faunaeur.org:taxname:115112; scientificName: Aegosoma scabricorne; order: Coleoptera; family: Cerambycidae; genus: Aegosoma; scientificNameAuthorship: Scopoli 1763; **Location:** country: Italy; stateProvince: Pavia; locality: SIC "Boschi di Vaccarizza" - V2; verbatimElevation: 65 m; verbatimCoordinates: 32T 519868E 4999488N; verbatimCoordinateSystem: UTM WGS 84; decimalLatitude: 45.148589; decimalLongitude: 9.252737; georeferencedBy: Silvia Stefanelli; georeferenceProtocol: GPS; **Identification:** identifiedBy: Carlo Pesarini; dateIdentified: 2011

##### Ecological interactions

###### Conservation status

Least Concern (European Environment Agency 2013).

##### Distribution

Albania, Austria, Belarus, Bosnia and Herzegovina, Bulgaria, Corsica, Crete, Croatia, Czech Republic, European Turkey, French mainland, Germany, Greek mainland, Hungary, Italian mainland, Macedonia, Romania, Russia South, Sardinia, Sicily, Slovakia, Slovenia, Spanish mainland, Switzerland, Ukraine, Yugoslavia, Near East (Fauna Europaea 2013).

##### Notes

The species appears in early summer and has nocturnal habits. The larva lives in all species of broadleaves, but mainly in large dead trees (Pesarini and Sabbadini 1994).

#### Cerambyx scopolii

(Fuessly, 1775)

##### Materials

- **Type status:** Other material. **Occurrence:** recordedBy: Silvia Stefanelli; individualCount: 1; lifeStage: adult; **Taxon:** taxonID: urn:lsid:faunaeur.org:taxname:114746; scientificName: Cerambyx scopolii; order: Coleoptera; family: Cerambycidae; genus: Cerambyx; scientificNameAuthorship: Fuessly 1775; **Location:** country: Italy; stateProvince: Pavia; locality: SIC "Boschi di Vaccarizza" - V2; verbatimElevation: 65 m; verbatimCoordinates: 32T 519868E 4999488N; verbatimCoordinateSystem: UTM WGS 84; decimalLatitude: 45.148589; decimalLongitude: 9.252737; georeferencedBy: Silvia Stefanelli; georeferenceProtocol: GPS; **Identification:** identificationID: Carlo Pesarini; identifiedBy: 2011

##### Ecological interactions

###### Conservation status

Least Concern (European Environment Agency 2013).

##### Distribution

Albania, Austria, Balearic Is., Belarus, Belgium, Bosnia and Herzegovina, Bulgaria, Corsica, Crete, Croatia, Czech Republic, Danish mainland, Estonia, European Turkey, French mainland, Germany, Greek mainland, Hungary, Italian mainland, Latvia, Lithuania, Luxembourg, Macedonia, Moldova Republic of, Norwegian mainland, Poland, Portuguese mainland, Romania, Russia Central, Russia East, Russia North, Russia Northwest, Russia South, Sardinia, Sicily, Slovakia, Slovenia, Spanish mainland, Sweden, Switzerland, The Netherlands, Ukraine, Yugoslavia, East Palaearctic, Near East (Fauna Europaea 2013).

##### Notes

This is the smallest European species of the genus. The larva is polyphagous and develops for two years in dead wood, but it also often lives in the wood of various damaged or diseased broadleaves. The adult is active for most of the spring and summer and appears in the flowers of shrubs and herbs and on old wood (Hůrka 2005, Pesarini and Sabbadini 1994).

#### Chlorophorus varius

(Muller, 1766)

##### Materials

- **Type status:** Other material. **Occurrence:** recordedBy: Silvia Stefanelli; individualCount: 3; lifeStage: adult; **Taxon:** taxonID: urn:lsid:faunaeur.org:taxname:114474; scientificName: Chlorophorus varius; order: Coleoptera; family: Cerambycidae; genus: Chlorophorus; scientificNameAuthorship: Muller 1766; **Location:** country: Italy; stateProvince: Pavia; locality: SIC "Boschi Siro Negri e Moriano" - BN1; verbatimElevation: 68 m; verbatimCoordinates: 32T 503258E 5007870N; verbatimCoordinateSystem: UTM WGS 84; decimalLatitude: 45.224312; decimalLongitude: 9.041499; georeferencedBy: Silvia Stefanelli; georeferenceProtocol: GPS; **Identification:** identifiedBy: Carlo Pesarini; dateIdentified: 2011
- **Type status:** Other material. **Occurrence:** recordedBy: Silvia Stefanelli; individualCount: 1; lifeStage: adult; **Taxon:** taxonID: urn:lsid:faunaeur.org:taxname:114474; scientificName: Chlorophorus varius; order: Coleoptera; family: Cerambycidae; genus: Chlorophorus; scientificNameAuthorship: Muller 1766; **Location:** country: Italy; stateProvince: Pavia; locality: SIC "Boschi Siro Negri e Moriano" - BN21; verbatimElevation: 66 m; verbatimCoordinates: 32T 506342E 5005026N; verbatimCoordinateSystem: UTM WGS 84; decimalLatitude: 45.198691; decimalLongitude: 9.080746; georeferencedBy: Silvia Stefanelli; georeferenceProtocol: GPS; **Identification:** identifiedBy: Carlo Pesarini; dateIdentified: 2011

##### Ecological interactions

###### Conservation status

Least Concern (European Environment Agency 2013).

##### Distribution

Albania, Austria, Belgium, Bosnia and Herzegovina, Bulgaria, Corsica, Croatia, Cyprus, Czech Republic, European Turkey, French mainland, Germany, Greek mainland, Hungary, Italian mainland, Liechtenstein, Lithuania, Macedonia, Malta, Moldova Republic of, Poland, Romania, Russia Central, Russia East, Russia South, Sardinia, Sicily, Slovakia, Slovenia, Spanish mainland, Switzerland, The Netherlands, Ukraine, Yugoslavia, East Palaearctic, Near East (Fauna Europaea 2013).

##### Notes

The species develops in the wood and dead branches of various broadleaves and occasionally in conifers; adults occur on flowers (Alexander 2002, Hůrka 2005).

#### Clytus arietis

(Linneaus, 1758)

##### Materials

- **Type status:** Other material. **Occurrence:** recordedBy: Silvia Stefanelli; individualCount: 1; lifeStage: adult; **Taxon:** taxonID: urn:lsid:faunaeur.org:taxname:114513; scientificName: Clytus arietis; order: Coleoptera; family: Cerambycidae; genus: Clytus; scientificNameAuthorship: Linnaeus 1758; **Location:** country: Italy; stateProvince: Pavia; locality: SIC "Boschi Siro Negri e Moriano" - BN1; verbatimElevation: 68 m; verbatimCoordinates: 32T 503258E 5007870N; verbatimCoordinateSystem: UTM WGS 84; decimalLatitude: 45.224312; decimalLongitude: 9.041499; georeferencedBy: Silvia Stefanelli; georeferenceProtocol: GPS; **Identification:** identifiedBy: Carlo Pesarini; dateIdentified: 2011
- **Type status:** Other material. **Occurrence:** recordedBy: Silvia Stefanelli; individualCount: 1; lifeStage: adult; **Taxon:** taxonID: urn:lsid:faunaeur.org:taxname:114513; scientificName: Clytus arietis; order: Coleoptera; family: Cerambycidae; genus: Clytus; scientificNameAuthorship: Linnaeus 1758; **Location:** country: Italy; stateProvince: Pavia; locality: SIC "Boschi Siro Negri e Moriano" - BN10; verbatimElevation: 76 m; verbatimCoordinates: 32T 504479E 5006332N; verbatimCoordinateSystem: UTM WGS 84; decimalLatitude: 45.210461; decimalLongitude: 9.057038; georeferencedBy: Silvia Stefanelli; georeferenceProtocol: GPS; **Identification:** identifiedBy: Carlo Pesarini; dateIdentified: 2011

##### Ecological interactions

###### Conservation status

Least Concern (European Environment Agency 2013).

##### Distribution

Albania, Austria, Belgium, Bosnia and Herzegovina, Bulgaria, Corsica, Croatia, Cyprus, Czech Republic, European Turkey, French mainland, Germany, Greek mainland, Hungary, Italian mainland, Liechtenstein, Lithuania, Macedonia, Malta, Moldova Republic of, Poland, Romania, Russia Central, Russia East, Russia South, Sardinia, Sicily, Slovakia, Slovenia, Spanish mainland, Switzerland, The Netherlands, Ukraine, Yugoslavia, East Palaearctic, Near East (Fauna Europaea 2013).

##### Notes

The species is active for most of the spring and summer. Larva develops under bark and in the wood of many broadleaves, and is often found on oaks, beech, and fruit trees (Hůrka 2005). The adults occur on flowers, but can also be found on the trunks of host plants (Pesarini and Sabbadini 1994).

#### Grammoptera ruficornis

(Fabricius, 1781)

Leptura ruficornis Fabricius, 1781 – Fauna Europaea (2013)
Leptura atra Fabricius, 1775 – Fauna Europaea (2013)

##### Materials

- **Type status:** Other material. **Occurrence:** recordedBy: Silvia Stefanelli; individualCount: 1; lifeStage: adult; **Taxon:** taxonID: urn:lsid:faunaeur.org:taxname:114987; scientificName: Grammoptera ruficornis; order: Coleoptera; family: Cerambycidae; genus: Grammoptera; scientificNameAuthorship: Fabricius 1781; **Location:** country: Italy; stateProvince: Pavia; locality: SIC "Boschi Siro Negri e Moriano" - BN5; verbatimElevation: 62 m; verbatimCoordinates: 32T 502886E 5008393N; verbatimCoordinateSystem: UTM WGS 84; decimalLatitude: 45.229029; decimalLongitude: 9.036770; georeferencedBy: Silvia Stefanelli; georeferenceProtocol: GPS; **Identification:** identifiedBy: Carlo Pesarini; dateIdentified: 2011
- **Type status:** Other material. **Occurrence:** recordedBy: Silvia Stefanelli; individualCount: 2; lifeStage: adult; **Taxon:** taxonID: urn:lsid:faunaeur.org:taxname:114987; scientificName: Grammoptera ruficornis; order: Coleoptera; family: Cerambycidae; genus: Grammoptera; scientificNameAuthorship: Fabricius 1781; **Location:** country: Italy; stateProvince: Pavia; locality: SIC "Boschi di Vaccarizza" - V2; verbatimElevation: 65 m; verbatimCoordinates: 32T 519868E 4999488N; verbatimCoordinateSystem: UTM WGS 84; decimalLatitude: 45.148589; decimalLongitude: 9.252737; georeferencedBy: Silvia Stefanelli; georeferenceProtocol: GPS; **Identification:** identifiedBy: Carlo Pesarini; dateIdentified: 2011

##### Distribution

Albania, Austria, Belarus, Belgium, Bosnia and Herzegovina, Britain I., Bulgaria, Croatia, Czech Republic, Danish mainland, Estonia, French mainland, Germany, Greek mainland, Hungary, Ireland, Italian mainland, Latvia, Liechtenstein, Lithuania, Luxembourg, Macedonia, Moldova Republic of, Northern Ireland, Norwegian mainland, Poland, Portuguese mainland, Romania, Russia East, Russia South, Sicily, Slovakia, Slovenia, Spanish mainland, Sweden, Switzerland, The Netherlands, Ukraine, Yugoslavia, Near East (Fauna Europaea 2013).

##### Notes

The species is the most common of the four Central European species of the genus. The larva is polyphagous and develops under bark on dry branch wood. The adult appears in May and June on flowers of various plants especially hawthorns and umbrellifers (Hůrka 2005).

#### Leiopus nebulosus

(Linneaus, 1758)

##### Materials

- **Type status:** Other material. **Occurrence:** recordedBy: Silvia Stefanelli; individualCount: 3; lifeStage: adult; **Taxon:** taxonID: urn:lsid:faunaeur.org:taxname:114028; scientificName: Leiopus nebulosus; order: Coleoptera; family: Cerambycidae; genus: Leiopus; scientificNameAuthorship: Linnaeus 1758; **Location:** country: Italy; stateProvince: Pavia; locality: SIC "Boschi Siro Negri e Moriano" - BN1; verbatimElevation: 68 m; verbatimCoordinates: 32T 503258E 5007870N; verbatimCoordinateSystem: UTM WGS 84; decimalLatitude: 45.224312; decimalLongitude: 9.041499; georeferencedBy: Silvia Stefanelli; georeferenceProtocol: GPS; **Identification:** identifiedBy: Carlo Pesarini; dateIdentified: 2011
- **Type status:** Other material. **Occurrence:** recordedBy: Silvia Stefanelli; individualCount: 1; lifeStage: adult; **Taxon:** taxonID: urn:lsid:faunaeur.org:taxname:114028; scientificName: Leiopus nebulosus; order: Coleoptera; family: Cerambycidae; genus: Leiopus; scientificNameAuthorship: Linnaeus 1758; **Location:** country: Italy; stateProvince: Pavia; locality: SIC "Boschi Siro Negri e Moriano" - BN5; verbatimElevation: 62 m; verbatimCoordinates: 32T 502886E 5008393N; verbatimCoordinateSystem: UTM WGS 84; decimalLatitude: 45.229029; decimalLongitude: 9.036770; georeferencedBy: Silvia Stefanelli; georeferenceProtocol: GPS; **Identification:** identifiedBy: Carlo Pesarini; dateIdentified: 2011
- **Type status:** Other material. **Occurrence:** recordedBy: Silvia Stefanelli; individualCount: 1; lifeStage: adult; **Taxon:** taxonID: urn:lsid:faunaeur.org:taxname:114028; scientificName: Leiopus nebulosus; order: Coleoptera; family: Cerambycidae; genus: Leiopus; scientificNameAuthorship: Linnaeus 1758; **Location:** country: Italy; stateProvince: Pavia; locality: SIC "Boschi Siro Negri e Moriano" - BN10; verbatimElevation: 76 m; verbatimCoordinates: 32T 504479E 5006332N; verbatimCoordinateSystem: UTM WGS 84; decimalLatitude: 45.210461; decimalLongitude: 9.057038; georeferencedBy: Silvia Stefanelli; georeferenceProtocol: GPS; **Identification:** identifiedBy: Carlo Pesarini; dateIdentified: 2011
- **Type status:** Other material. **Occurrence:** recordedBy: Silvia Stefanelli; individualCount: 3; lifeStage: adult; **Taxon:** taxonID: urn:lsid:faunaeur.org:taxname:114028; scientificName: Leiopus nebulosus; order: Coleoptera; family: Cerambycidae; genus: Leiopus; scientificNameAuthorship: Linnaeus 1758; **Location:** country: Italy; stateProvince: Pavia; locality: SIC "Boschi di Vaccarizza" - V2; verbatimElevation: 65 m; verbatimCoordinates: 32T 519868E 4999488N; verbatimCoordinateSystem: UTM WGS 84; decimalLatitude: 45.148589; decimalLongitude: 9.252737; georeferencedBy: Silvia Stefanelli; georeferenceProtocol: GPS; **Identification:** identifiedBy: Carlo Pesarini; dateIdentified: 2011

##### Distribution

Albania, Austria, Belarus, Belgium, Bosnia and Herzegovina, Britain I., Bulgaria, Corsica, Croatia, Czech Republic, Danish mainland, Estonia, European Turkey, Finland, French mainland, Germany, Greek mainland, Hungary, Italian mainland, Latvia, Liechtenstein, Lithuania, Luxembourg, Moldova, Republic of, Norwegian mainland, Poland, Portuguese mainland, Romania, Russia Central, Russia East, Russia North, Russia Northwest, Russia South, Sicily, Slovakia, Slovenia, Spanish mainland, Sweden, Switzerland, The Netherlands, Ukraine, Yugoslavia, East Palaearctic, Near East (Fauna Europaea 2013).

##### Notes

The larva is polyphagous and develops under bark on branches and trunks of various dead broadleaves, mainly oak, and also rarely in conifers. The adult is found from May to August on wood in broadleaves woodland ranging from lowlands to mountains (Alexander 2002, Hůrka 2005).

#### Leptura aurulenta

(Fabricius, 1792)

##### Materials

- **Type status:** Other material. **Occurrence:** recordedBy: Silvia Stefanelli; individualCount: 1; lifeStage: adult; **Taxon:** taxonID: urn:lsid:faunaeur.org:taxname:114959; scientificName: Leptura aurulenta; order: Coleoptera; family: Cerambycidae; genus: Leptura; scientificNameAuthorship: Fabricius 1792; **Location:** country: Italy; stateProvince: Pavia; locality: SIC "Boschi di Vaccarizza" - V1; verbatimElevation: 62 m; verbatimCoordinates: 32T 519272E 4999526N; verbatimCoordinateSystem: UTM WGS 84; decimalLatitude: 45.148947; decimalLongitude: 9.245157; georeferencedBy: Silvia Stefanelli; georeferenceProtocol: GPS; **Identification:** identifiedBy: Carlo Pesarini; dateIdentified: 2011

##### Distribution

Albania, Andorra, Austria, Belgium, Bosnia and Herzegovina, Britain I., Bulgaria, Corsica, Croatia, Czech Republic, European Turkey, French mainland, Germany, Greek mainland, Hungary, Ireland, Italian mainland, Luxembourg, Macedonia, Poland, Portuguese mainland, Romania, Slovakia, Slovenia, Spanish mainland, Switzerland, Ukraine, Yugoslavia, Near East, North Africa (Fauna Europaea 2013).

##### Notes

The species is widespread from the plains to the mountains. The larva develops in the cambial layer of large sections of freshly dead broadleaves wood. The adult is usually found on oaks, and rarely occur on flowers. (Alexander and Anderson 2012, Pesarini and Sabbadini 1994).

#### Mesosa nebulosa

(Fabricius, 1781)

##### Materials

- **Type status:** Other material. **Occurrence:** recordedBy: Silvia Stefanelli; individualCount: 1; lifeStage: adult; **Taxon:** taxonID: urn:lsid:faunaeur.org:taxname:114197; scientificName: Mesosa nebulosa; order: Coleoptera; family: Cerambycidae; genus: Mesosa; scientificNameAuthorship: Fabricius 1781; **Location:** country: Italy; stateProvince: Pavia; locality: SIC "Boschi Siro Negri e Moriano" - BN5; verbatimElevation: 62 m; verbatimCoordinates: 32T 502886E 5008393N; verbatimCoordinateSystem: UTM WGS 84; decimalLatitude: 45.229029; decimalLongitude: 9.036770; georeferencedBy: Silvia Stefanelli; georeferenceProtocol: GPS; **Identification:** identifiedBy: Carlo Pesarini; dateIdentified: 2011

##### Distribution

Albania, Austria, Belarus, Belgium, Bosnia and Herzegovina, Britain I., Bulgaria, Corsica, Crete, Croatia, Czech Republic, Danish mainland, European Turkey, French mainland, Germany, Greek mainland, Hungary, Italian mainland, Luxembourg, Moldova Republic of, Norwegian mainland, Poland, Portuguese mainland, Romania, Russia Central, Russia South, Sardinia, Sicily, Slovakia, Slovenia, Spanish mainland, Sweden, Switzerland, The Netherlands, Ukraine, Yugoslavia, Near East, North Africa (Fauna Europaea 2013).

##### Notes

The species is polyphagous. The larva develops in the wood of different species of broadleaves. The adult appears in early spring and remains active for a short period (Pesarini and Sabbadini 1994).

#### Morimus asper

(Sulzer, 1776)

Cerambyx asper Sulzer, 1776 – Fauna Europaea (2013)
Morimus ganglbaueri   Reitter, 1894 – Fauna Europaea (2013)

##### Materials

- **Type status:** Other material. **Occurrence:** recordedBy: Silvia Stefanelli; individualCount: 4; lifeStage: adult; **Taxon:** taxonID: urn:lsid:faunaeur.org:taxname:114117; scientificName: Morimus asper; order: Coleoptera; family: Cerambycidae; genus: Morimus; scientificNameAuthorship: Sulzer 1776; **Location:** country: Italy; stateProvince: Pavia; locality: SIC "Boschi di Vaccarizza" - V1; verbatimElevation: 62 m; verbatimCoordinates: 32T 519272E 4999526N; verbatimCoordinateSystem: UTM WGS 84; decimalLatitude: 45.148947; decimalLongitude: 9.245157; georeferencedBy: Silvia Stefanelli; georeferenceProtocol: GPS; **Identification:** identifiedBy: Carlo Pesarini; dateIdentified: 2011

##### Distribution

Albania, Austria, Bosnia and Herzegovina, Bulgaria, Corsica, Croatia, European Turkey, French mainland, Greek mainland, Italian mainland, Romania, San Marino, Sardinia, Sicily, Spanish mainland, Switzerland (Fauna Europaea 2013).

##### Notes

The species has a strong sexual dimorphism. The larva is polyphagous and develops in both deciduous and coniferous trees, but prefers the first. The adult can be found on the trunks of the same host plants (Pesarini and Sabbadini 1994).

#### Neoclytus acuminatus

(Fabricius, 1775)

##### Materials

- **Type status:** Other material. **Occurrence:** recordedBy: Silvia Stefanelli; individualCount: 3; lifeStage: adult; **Taxon:** taxonID: urn:lsid:faunaeur.org:taxname:114492; scientificName: Neoclytus acuminatus; order: Coleoptera; family: Cerambycidae; genus: Neoclytus; scientificNameAuthorship: Fabricius 1775; **Location:** country: Italy; stateProvince: Pavia; locality: SIC "Boschi Siro Negri e Moriano" - BN21; verbatimElevation: 66 m; verbatimCoordinates: 32T 506342E 5005026N; verbatimCoordinateSystem: UTM WGS 84; decimalLatitude: 45.198691; decimalLongitude: 9.080746; georeferencedBy: Silvia Stefanelli; georeferenceProtocol: GPS; **Identification:** identificationID: Carlo Pesarini; identifiedBy: 2011
- **Type status:** Other material. **Occurrence:** recordedBy: Silvia Stefanelli; individualCount: 1; lifeStage: adult; **Taxon:** taxonID: urn:lsid:faunaeur.org:taxname:114492; scientificName: Neoclytus acuminatus; order: Coleoptera; family: Cerambycidae; genus: Neoclytus; scientificNameAuthorship: Fabricius 1775; **Location:** country: Italy; stateProvince: Pavia; locality: SIC "Boschi Siro Negri e Moriano" - BN5; verbatimElevation: 62 m; verbatimCoordinates: 32T 502886E 5008393N; verbatimCoordinateSystem: UTM WGS 84; decimalLatitude: 45.229029; decimalLongitude: 9.036770; georeferencedBy: Silvia Stefanelli; georeferenceProtocol: GPS; **Identification:** identificationID: Carlo Pesarini; identifiedBy: 2011

##### Distribution

Croatia, Hungary, Italian mainland, Slovenia, Switzerland, Nearctic region (Fauna Europaea 2013).

##### Notes

The species is thermophilic. It is found on many hardwoods as rosa, oak, lime, ash, grape, cercis, chestnut, willow, hornbeam, evonymus, birch, robinia, and hibiscus trees, but is also more rarely found in conifers such as fir trees (Pesarini and Sabbadini 1994).

#### Phymatodes testaceus

(Linneaus, 1958)

Callidium violaceum Rossi, 1790 – Fauna Europaea (2013)
Callidium italicus Gmelin, 1790 – Fauna Europaea (2013)
Callidium ruficollis Fabricius, 1781 – Fauna Europaea (2013)

##### Materials

- **Type status:** Other material. **Occurrence:** recordedBy: Silvia Stefanelli; individualCount: 1; lifeStage: adult; **Taxon:** taxonID: urn:lsid:faunaeur.org:taxname:114557; scientificName: Phymatodes testaceus; order: Coleoptera; family: Cerambycidae; genus: Phymatodes; scientificNameAuthorship: Linnaeus 1758; **Location:** country: Italy; stateProvince: Pavia; locality: SIC "Boschi Siro Negri e Moriano" - BN1; verbatimElevation: 68 m; verbatimCoordinates: 32T 503258E 5007870N; verbatimCoordinateSystem: UTM WGS 84; decimalLatitude: 45.224312; decimalLongitude: 9.041499; georeferencedBy: Silvia Stefanelli; georeferenceProtocol: GPS; **Identification:** identifiedBy: Carlo Pesarini; dateIdentified: 2011
- **Type status:** Other material. **Occurrence:** recordedBy: Silvia Stefanelli; individualCount: 1; lifeStage: adult; **Taxon:** taxonID: urn:lsid:faunaeur.org:taxname:114557; scientificName: Phymatodes testaceus; order: Coleoptera; family: Cerambycidae; genus: Phymatodes; scientificNameAuthorship: Linnaeus 1758; **Location:** country: Italy; stateProvince: Pavia; locality: SIC "Boschi Siro Negri e Moriano" - BN10; verbatimElevation: 76 m; verbatimCoordinates: 32T 504479E 5006332N; verbatimCoordinateSystem: UTM WGS 84; decimalLatitude: 45.210461; decimalLongitude: 9.057038; georeferencedBy: Silvia Stefanelli; georeferenceProtocol: GPS; **Identification:** identifiedBy: Carlo Pesarini; dateIdentified: 2011

##### Ecological interactions

###### Conservation status

Least Concern (European Environment Agency 2013).

##### Distribution

Albania, Austria, Belarus, Belgium, Bosnia and Herzegovina, Britain I., Bulgaria, Corsica, Crete, Croatia, Cyprus, Czech Republic, Danish mainland, Estonia, European Turkey, Finland, French mainland, Germany, Greek mainland, Hungary, Italian mainland, Latvia, Liechtenstein, Lithuania, Luxembourg, Macedonia, Moldova Republic of, Norwegian mainland, Poland, Portuguese mainland, Romania, Russia Central, Russia East, Russia North, Russia Northwest, Russia South, Sardinia, Sicily, Slovakia, Slovenia, Spanish mainland, Sweden, Switzerland, The Netherlands, Ukraine, Yugoslavia, East Palaearctic, Near East, Nearctic region, North Africa, Oriental region (Fauna Europaea 2013).

##### Notes

The species develops in the dead branches and dead logs of various broadleaves, mainly oak, and also in conifers. The larva makes characteristic borings in the bark and sapwood. The adult is crepuscular and attracted to light and sweet secretions (Alexander 2002).

#### Pogonocherus hispidus

(Linneaus, 1958)

##### Materials

- **Type status:** Other material. **Occurrence:** recordedBy: Silvia Stefanelli; individualCount: 2; lifeStage: adult; **Taxon:** taxonID: urn:lsid:faunaeur.org:taxname:114053; scientificName: Pogonocherus hispidus; order: Coleoptera; family: Cerambycidae; genus: Pogonocherus; **Location:** country: Italy; stateProvince: Pavia; locality: SIC "Boschi Siro Negri e Moriano" - BN1; verbatimElevation: 68 m; verbatimCoordinates: 32T 503258E 5007870N; verbatimCoordinateSystem: UTM WGS 84; decimalLatitude: 45.224312; decimalLongitude: 9.041499; georeferencedBy: Silvia Stefanelli; georeferenceProtocol: GPS; **Identification:** identifiedBy: Carlo Pesarini; dateIdentified: 2011
- **Type status:** Other material. **Occurrence:** recordedBy: Silvia Stefanelli; individualCount: 4; lifeStage: adult; **Taxon:** taxonID: urn:lsid:faunaeur.org:taxname:114053; scientificName: Pogonocherus hispidus; order: Coleoptera; family: Cerambycidae; genus: Pogonocherus; **Location:** country: Italy; stateProvince: Pavia; locality: SIC "Boschi Siro Negri e Moriano" - BN5; verbatimElevation: 62 m; verbatimCoordinates: 32T 502886E 5008393N; verbatimCoordinateSystem: UTM WGS 84; decimalLatitude: 45.229029; decimalLongitude: 9.036770; georeferencedBy: Silvia Stefanelli; georeferenceProtocol: GPS; **Identification:** identifiedBy: Carlo Pesarini; dateIdentified: 2011
- **Type status:** Other material. **Occurrence:** recordedBy: Silvia Stefanelli; individualCount: 2; lifeStage: adult; **Taxon:** taxonID: urn:lsid:faunaeur.org:taxname:114053; scientificName: Pogonocherus hispidus; order: Coleoptera; family: Cerambycidae; genus: Pogonocherus; **Location:** country: Italy; stateProvince: Pavia; locality: SIC "Boschi di Vaccarizza" - V1; verbatimElevation: 62 m; verbatimCoordinates: 32T 519272E 4999526N; verbatimCoordinateSystem: UTM WGS 84; decimalLatitude: 45.148947; decimalLongitude: 9.245157; georeferencedBy: Silvia Stefanelli; georeferenceProtocol: GPS; **Identification:** identifiedBy: Carlo Pesarini; dateIdentified: 2011

##### Distribution

Albania, Austria, Belarus, Belgium, Bosnia and Herzegovina, Britain I., Bulgaria, Corsica, Croatia, Czech Republic, Danish mainland, Estonia, Finland, French mainland, Germany, Greek mainland, Hungary, Ireland, Italian mainland, Latvia, Liechtenstein, Lithuania, Luxembourg, Moldova Republic of, Norwegian mainland, Poland, Portuguese mainland, Romania, Russia Central, Russia East, Russia North, Russia Northwest, Russia South, Sicily, Slovakia, Slovenia, Spanish mainland, Sweden, Switzerland, The Netherlands, Ukraine, Yugoslavia, North Africa (Fauna Europaea 2013).

##### Notes

The species develops in the thin dead branches of a variety of broadleaves especially in old hedgerows (Alexander 2002).

#### Prionus (Prionus) coriarius

(Linneaus, 1958)

##### Materials

- **Type status:** Other material. **Occurrence:** recordedBy: Silvia Stefanelli; individualCount: 1; lifeStage: adult; **Taxon:** taxonID: urn:lsid:faunaeur.org:taxname:115097; scientificName: Prionus coriarius; order: Coleoptera; family: Cerambycidae; genus: Prionus; scientificNameAuthorship: Linnaeus 1758; **Location:** country: Italy; stateProvince: Pavia; locality: SIC "Boschi Siro Negri e Moriano" - BN1; verbatimElevation: 68 m; verbatimCoordinates: 32T 503258E 5007870N; verbatimCoordinateSystem: UTM WGS 84; decimalLatitude: 45.224312; decimalLongitude: 9.041499; georeferencedBy: Silvia Stefanelli; georeferenceProtocol: GPS; **Identification:** identifiedBy: Carlo Pesarini; dateIdentified: 2011
- **Type status:** Other material. **Occurrence:** recordedBy: Silvia Stefanelli; individualCount: 3; lifeStage: adult; **Taxon:** taxonID: urn:lsid:faunaeur.org:taxname:115097; scientificName: Prionus coriarius; order: Coleoptera; family: Cerambycidae; genus: Prionus; scientificNameAuthorship: Linnaeus 1758; **Location:** country: Italy; stateProvince: Pavia; locality: SIC "Boschi Siro Negri e Moriano" - BN5; verbatimElevation: 62 m; verbatimCoordinates: 32T 502886E 5008393N; verbatimCoordinateSystem: UTM WGS 84; decimalLatitude: 45.229029; decimalLongitude: 9.036770; georeferencedBy: Silvia Stefanelli; georeferenceProtocol: GPS; **Identification:** identifiedBy: Carlo Pesarini; dateIdentified: 2011
- **Type status:** Other material. **Occurrence:** recordedBy: Silvia Stefanelli; individualCount: 4; lifeStage: adult; **Taxon:** taxonID: urn:lsid:faunaeur.org:taxname:115097; scientificName: Prionus coriarius; order: Coleoptera; family: Cerambycidae; genus: Prionus; scientificNameAuthorship: Linnaeus 1758; **Location:** country: Italy; stateProvince: Pavia; locality: SIC "Boschi Siro Negri e Moriano" - BN10; verbatimElevation: 76 m; verbatimCoordinates: 32T 504479E 5006332N; verbatimCoordinateSystem: UTM WGS 84; decimalLatitude: 45.210461; decimalLongitude: 9.057038; georeferencedBy: Silvia Stefanelli; georeferenceProtocol: GPS; **Identification:** identifiedBy: Carlo Pesarini; dateIdentified: 2011

##### Ecological interactions

###### Conservation status

Least Concern (European Environment Agency 2013).

##### Distribution

Albania, Austria, Belarus, Belgium, Bosnia and Herzegovina, Britain I., Bulgaria, Croatia, Czech Republic, Danish mainland, Estonia, Finland, French mainland, Germany, Greek mainland, Hungary, Italian mainland, Latvia, Liechtenstein, Lithuania, Luxembourg, Macedonia, Moldova Republic of, Norwegian mainland, Poland, Portuguese mainland, Romania, Russia Central, Russia East, Russia North, Russia Northwest, Russia South, Sicily, Slovakia, Slovenia, Spanish mainland, Sweden, Switzerland, The Netherlands, Ukraine, Yugoslavia, Near East, North Africa (Fauna Europaea 2013).

##### Notes

The species is widespread in both the plains and the mountains. The larva develops for three years in dead wood, mainly in stumps and their roots, and reaches up to 70mm in size. The adult appears in the summer in broadleaves and mixed forests, and also rarely in conifers. It flies at dusk and during the night. It is a species with a declining population (Hůrka 2005, Pesarini and Sabbadini 1994).

#### Pseudovadonia livida

(Fabricius, 1776)

##### Materials

- **Type status:** Other material. **Occurrence:** recordedBy: Silvia Stefanelli; individualCount: 2; lifeStage: adult; **Taxon:** taxonID: urn:lsid:faunaeur.org:taxname:114867; scientificName: Pseudovadonia livida; order: Coleoptera; family: Cerambycidae; genus: Pseudovadonia; scientificNameAuthorship: Fabricius 1776; **Location:** country: Italy; stateProvince: Pavia; locality: SIC "Boschi Siro Negri e Moriano" - BN1; verbatimElevation: 68 m; verbatimCoordinates: 32T 503258E 5007870N; verbatimCoordinateSystem: UTM WGS 84; decimalLatitude: 45.224312; decimalLongitude: 9.041499; georeferencedBy: Silvia Stefanelli; georeferenceProtocol: GPS; **Identification:** identifiedBy: Carlo Pesarini; dateIdentified: 2011
- **Type status:** Other material. **Occurrence:** recordedBy: Silvia Stefanelli; individualCount: 2; lifeStage: adult; **Taxon:** taxonID: urn:lsid:faunaeur.org:taxname:114867; scientificName: Pseudovadonia livida; order: Coleoptera; family: Cerambycidae; genus: Pseudovadonia; scientificNameAuthorship: Fabricius 1776; **Location:** country: Italy; stateProvince: Pavia; locality: SIC "Boschi Siro Negri e Moriano" - BN5; verbatimElevation: 62 m; verbatimCoordinates: 32T 502886E 5008393N; verbatimCoordinateSystem: UTM WGS 84; decimalLatitude: 45.229029; decimalLongitude: 9.036770; georeferencedBy: Silvia Stefanelli; georeferenceProtocol: GPS; **Identification:** identifiedBy: Carlo Pesarini; dateIdentified: 2011
- **Type status:** Other material. **Occurrence:** recordedBy: Silvia Stefanelli; individualCount: 3; lifeStage: adult; **Taxon:** taxonID: urn:lsid:faunaeur.org:taxname:114867; scientificName: Pseudovadonia livida; order: Coleoptera; family: Cerambycidae; genus: Pseudovadonia; scientificNameAuthorship: Fabricius 1776; **Location:** country: Italy; stateProvince: Pavia; locality: SIC "Boschi Siro Negri e Moriano" - BN10; verbatimElevation: 76 m; verbatimCoordinates: 32T 504479E 5006332N; verbatimCoordinateSystem: UTM WGS 84; decimalLatitude: 45.210461; decimalLongitude: 9.057038; georeferencedBy: Silvia Stefanelli; georeferenceProtocol: GPS; **Identification:** identifiedBy: Carlo Pesarini; dateIdentified: 2011

##### Distribution

Albania, Andorra, Austria, Belarus, Belgium, Bosnia and Herzegovina, Britain I., Bulgaria, Croatia, Czech Republic, Danish mainland, Estonia, European Turkey, French mainland, Germany, Greek mainland, Hungary, Ireland, Italian mainland, Latvia, Lithuania, Luxembourg, Macedonia, Moldova Republic of, Norwegian mainland, Poland, Portuguese mainland, Romania, Russia Central, Russia East, Russia North, Russia Northwest, Russia South, Sicily, Slovakia, Slovenia, Spanish mainland, Sweden, Switzerland, The Netherlands, Ukraine, Yugoslavia, East Palaearctic, Near East (Fauna Europaea (2013)).

##### Notes

The larva lives in the soil and feeds on the mycelia of fairy ring champignons. The adult appears from June to August on various flowers, mainly in meadows (Hůrka 2005).

#### Stenurella melanura

(Linneaus, 1958)

##### Materials

- **Type status:** Other material. **Occurrence:** recordedBy: Silvia Stefanelli; individualCount: 3; lifeStage: adult; **Taxon:** taxonID: urn:lsid:faunaeur.org:taxname:114843; scientificName: Stenurella melanura; order: Coleoptera; family: Cerambycidae; genus: Stenurella; scientificNameAuthorship: Linnaeus 1758; **Location:** country: Italy; stateProvince: Pavia; locality: SIC "Boschi Siro Negri e Moriano" - BN1; verbatimElevation: 68 m; verbatimCoordinates: 32T 503258E 5007870N; verbatimCoordinateSystem: UTM WGS 84; decimalLatitude: 45.224312; decimalLongitude: 9.041499; georeferencedBy: Silvia Stefanelli; georeferenceProtocol: GPS; **Identification:** identifiedBy: Carlo Pesarini; dateIdentified: 2011
- **Type status:** Other material. **Occurrence:** recordedBy: Silvia Stefanelli; individualCount: 1; lifeStage: adult; **Taxon:** taxonID: urn:lsid:faunaeur.org:taxname:114843; scientificName: Stenurella melanura; order: Coleoptera; family: Cerambycidae; genus: Stenurella; scientificNameAuthorship: Linnaeus 1758; **Location:** country: Italy; stateProvince: Pavia; locality: SIC "Boschi Siro Negri e Moriano" - BN21; verbatimElevation: 66 m; verbatimCoordinates: 32T 506342E 5005026N; verbatimCoordinateSystem: UTM WGS 84; decimalLatitude: 45.198691; decimalLongitude: 9.080746; georeferencedBy: Silvia Stefanelli; georeferenceProtocol: GPS; **Identification:** identifiedBy: Carlo Pesarini; dateIdentified: 2011
- **Type status:** Other material. **Occurrence:** recordedBy: Silvia Stefanelli; individualCount: 4; lifeStage: adult; **Taxon:** taxonID: urn:lsid:faunaeur.org:taxname:114843; scientificName: Stenurella melanura; order: Coleoptera; family: Cerambycidae; genus: Stenurella; scientificNameAuthorship: Linnaeus 1758; **Location:** country: Italy; stateProvince: Pavia; locality: SIC "Boschi Siro Negri e Moriano" - BN5; verbatimElevation: 62 m; verbatimCoordinates: 32T 502886E 5008393N; verbatimCoordinateSystem: UTM WGS 84; decimalLatitude: 45.229029; decimalLongitude: 9.036770; georeferencedBy: Silvia Stefanelli; georeferenceProtocol: GPS; **Identification:** identifiedBy: Carlo Pesarini; dateIdentified: 2011
- **Type status:** Other material. **Occurrence:** recordedBy: Silvia Stefanelli; individualCount: 1; lifeStage: adult; **Taxon:** taxonID: urn:lsid:faunaeur.org:taxname:114843; scientificName: Stenurella melanura; order: Coleoptera; family: Cerambycidae; genus: Stenurella; scientificNameAuthorship: Linnaeus 1758; **Location:** country: Italy; stateProvince: Pavia; locality: SIC "Boschi Siro Negri e Moriano" - BN10; verbatimElevation: 76 m; verbatimCoordinates: 32T 504479E 5006332N; verbatimCoordinateSystem: UTM WGS 84; decimalLatitude: 45.210461; decimalLongitude: 9.057038; georeferencedBy: Silvia Stefanelli; georeferenceProtocol: GPS; **Identification:** identifiedBy: Carlo Pesarini; dateIdentified: 2011
- **Type status:** Other material. **Occurrence:** recordedBy: Silvia Stefanelli; individualCount: 1; lifeStage: adult; **Taxon:** taxonID: urn:lsid:faunaeur.org:taxname:114843; scientificName: Stenurella melanura; order: Coleoptera; family: Cerambycidae; genus: Stenurella; scientificNameAuthorship: Linnaeus 1758; **Location:** country: Italy; stateProvince: Pavia; locality: SIC "Boschi di Vaccarizza" - V1; verbatimElevation: 62 m; verbatimCoordinates: 32T 519272E 4999526N; verbatimCoordinateSystem: UTM WGS 84; decimalLatitude: 45.148947; decimalLongitude: 9.245157; georeferencedBy: Silvia Stefanelli; georeferenceProtocol: GPS; **Identification:** identifiedBy: Carlo Pesarini; dateIdentified: 2011

##### Distribution

Albania, Austria, Belarus, Belgium, Bosnia and Herzegovina, Britain I., Bulgaria, Croatia, Czech Republic, Danish mainland, Estonia, European Turkey, Finland, French mainland, Germany, Greek mainland, Hungary, Italian mainland, Latvia, Liechtenstein, Lithuania, Luxembourg, Macedonia, Moldova Republic of, Norwegian mainland, Poland, Portuguese mainland, Romania, Russia Central, Russia East, Russia North, Russia Northwest, Russia South, Slovakia, Slovenia,Spanish mainland, Sweden, Switzerland, The Netherlands, Ukraine, Yugoslavia, East Palaearctic, Near East, Oriental region (Fauna Europaea 2013).

##### Notes

The species is very common and lives in forests, meadows, and glades from lowlands to mountains. The larva develops in the rotting wood of broadleaves and conifers. Adults in flowers (Fauna Europaea 2013).

#### Stictoleptura cordigera

(Fuessly, 1775)

Leptura cordigera Fuessly, 1775 – Fauna Europaea (2013)

##### Materials

- **Type status:** Other material. **Occurrence:** recordedBy: Silvia Stefanelli; individualCount: 1; lifeStage: adult; **Taxon:** taxonID: urn:lsid:faunaeur.org:taxname:114936; scientificName: Stictoleptura cordigera; order: Coleoptera; family: Cerambycidae; genus: Stictoleptura; scientificNameAuthorship: Fuessly 1775; **Location:** country: Italy; stateProvince: Pavia; locality: SIC "Boschi di Vaccarizza" - V1; verbatimElevation: 62 m; verbatimCoordinates: 32T 519272E 4999526N; verbatimCoordinateSystem: UTM WGS 84; decimalLatitude: 45.148947; decimalLongitude: 9.245157; georeferencedBy: Silvia Stefanelli; georeferenceProtocol: GPS; **Identification:** identifiedBy: Carlo Pesarini; dateIdentified: 2011

##### Distribution

Central and Southern Europe, Asia Minor, Syria and Caucaso (Fauna Europaea 2013).

##### Notes

The larva develops in the wood of different species of broadleaves. The adult occur on flowers. (Pesarini and Sabbadini 1994).

#### Strangalia attenuata

(Linneaus, 1958)

##### Materials

- **Type status:** Other material. **Occurrence:** recordedBy: Silvia Stefanelli; individualCount: 4; lifeStage: adult; **Taxon:** taxonID: urn:lsid:faunaeur.org:taxname:114856; scientificName: Strangalia attenuata; order: Coleoptera; family: Cerambycidae; genus: Strangalia; scientificNameAuthorship: Linnaeus 1758; **Location:** country: Italy; stateProvince: Pavia; locality: SIC "Boschi Siro Negri e Moriano" - BN1; verbatimElevation: 68 m; verbatimCoordinates: 32T 503258E 5007870N; verbatimCoordinateSystem: UTM WGS 84; decimalLatitude: 45.224312; decimalLongitude: 9.041499; georeferencedBy: Silvia Stefanelli; georeferenceProtocol: GPS; **Identification:** identifiedBy: Carlo Pesarini; dateIdentified: 2011

##### Distribution

Albania, Austria, Belarus, Belgium, Bosnia and Herzegovina, Bulgaria, Corsica, Croatia, Czech Republic, Danish mainland, Estonia, European Turkey, Finland, French mainland, Germany, Greek mainland, Hungary, Italian mainland, Latvia, Liechtenstein, Lithuania, Luxembourg, Macedonia, Moldova Republic of, Norwegian mainland, Poland, Romania, Russia Central, Russia East, Russia North, Russia Northwest, Russia South, Slovakia, Slovenia, Spanish mainland, Sweden, Switzerland, The Netherlands, Ukraine, Yugoslavia, East Palaearctic, Near East, Oriental region (Fauna Europaea 2013).

##### Notes

The species is more common in the plains than in the mountains. The larva develops in different species of broadleaves. The adult occur on flowers and appears during late spring and summer (Pesarini and Sabbadini 1994).

#### Tetrops praeustus

(Linneaus, 1958)

##### Materials

- **Type status:** Other material. **Occurrence:** recordedBy: Silvia Stefanelli; individualCount: 1; lifeStage: adult; **Taxon:** taxonID: urn:lsid:faunaeur.org:taxname:113893; scientificName: Tetrops praeustus; order: Coleoptera; family: Cerambycidae; genus: Tetrops; scientificNameAuthorship: Linnaeus 1758; **Location:** country: Italy; stateProvince: Pavia; locality: SIC "Boschi Siro Negri e Moriano" - BN5; verbatimElevation: 62 m; verbatimCoordinates: 32T 502886E 5008393N; verbatimCoordinateSystem: UTM WGS 84; decimalLatitude: 45.229029; decimalLongitude: 9.036770; georeferencedBy: Silvia Stefanelli; georeferenceProtocol: GPS; **Identification:** identifiedBy: Carlo Pesarini; dateIdentified: 2011

##### Distribution

Albania, Austria, Belarus, Belgium, Bosnia and Herzegovina, Britain I., Bulgaria, Croatia, Czech Republic, Danish mainland, Estonia, European Turkey, Finland, French mainland, Germany, Greek mainland, Hungary, Italian mainland, Latvia, Liechtenstein, Lithuania, Luxembourg, Macedonia, Moldova Republic of, North Aegean Is., Norwegian mainland, Poland, Portuguese mainland, Romania, Russia Central, Russia East, Russia North, Russia Northwest, Russia South, Sardinia, Sicily, Slovakia, Slovenia, Spanish mainland, Sweden, Switzerland, The Netherlands, Ukraine, Yugoslavia, East Palaearctic, Near East, North Africa (Fauna Europaea 2013).

##### Notes

The larva develops under the bark of the dry branch wood of roses and hawthorns, as well as, blackthorns and other fruit trees. The adult appears beginning in April on the twigs, leaves, and flowers of orchards and forest edges (Hůrka 2005).

#### Xylotrechus antilope

(Schonherr, 1817)

##### Materials

- **Type status:** Other material. **Occurrence:** recordedBy: Silvia Stefanelli; individualCount: 1; lifeStage: adult; **Taxon:** taxonID: urn:lsid:faunaeur.org:taxname:114531; scientificName: Xylotrechus antilope; order: Coleoptera; family: Cerambycidae; genus: Xylotrechus; scientificNameAuthorship: Schonherr 1817; **Location:** country: Italy; stateProvince: Pavia; locality: SIC "Boschi Siro Negri e Moriano" - BN5; verbatimElevation: 62 m; verbatimCoordinates: 32T 502886E 5008393N; verbatimCoordinateSystem: UTM WGS 84; decimalLatitude: 45.229029; decimalLongitude: 9.036770; georeferencedBy: Silvia Stefanelli; georeferenceProtocol: GPS; **Identification:** identifiedBy: Carlo Pesarini; dateIdentified: 2011

##### Ecological interactions

###### Conservation status

Least Concern (European Environment Agency 2013).

##### Distribution

Albania, Austria, Belarus, Belgium, Bosnia and Herzegovina, Britain I., Bulgaria, Corsica, Croatia, Cyprus, Czech Republic, European Turkey, French mainland, Germany, Greek mainland, Hungary, Italian mainland, Moldova Republic of, Norwegian mainland, Poland, Portuguese mainland, Romania, Russia Central, Russia South, Sicily, Slovakia, Slovenia, Spanish mainland, Sweden, Switzerland, Ukraine, Yugoslavia, Near East, North Africa (Fauna Europaea 2013).

##### Notes

The species is more widespread in the Mediterranean area than in continental regions. The larva develops only in oaks. The adult appears during spring and summer on the logs of the host plants (Pesarini and Sabbadini 1994).

#### Xylotrechus rusticus

(Linneaus, 1958)

##### Materials

- **Type status:** Other material. **Occurrence:** recordedBy: Silvia Stefanelli; individualCount: 19; lifeStage: adult; **Taxon:** taxonID: urn:lsid:faunaeur.org:taxname:114524; scientificName: Xylotrechus rusticus; order: Coleoptera; family: Cerambycidae; genus: Xylotrechus; scientificNameAuthorship: Linnaeus 1758; **Location:** country: Italy; stateProvince: Pavia; locality: SIC "Boschi Siro Negri e Moriano" - BN1; verbatimElevation: 68 m; verbatimCoordinates: 32T 503258E 5007870N; verbatimCoordinateSystem: UTM WGS 84; decimalLatitude: 45.224312; decimalLongitude: 9.041499; georeferencedBy: Silvia Stefanelli; georeferenceProtocol: GPS; **Identification:** identifiedBy: Carlo Pesarini; dateIdentified: 2011
- **Type status:** Other material. **Occurrence:** recordedBy: Silvia Stefanelli; individualCount: 2; lifeStage: adult; **Taxon:** taxonID: urn:lsid:faunaeur.org:taxname:114524; scientificName: Xylotrechus rusticus; order: Coleoptera; family: Cerambycidae; genus: Xylotrechus; scientificNameAuthorship: Linnaeus 1758; **Location:** country: Italy; stateProvince: Pavia; locality: SIC "Boschi Siro Negri e Moriano" - BN21; verbatimElevation: 66 m; verbatimCoordinates: 32T 506342E 5005026N; verbatimCoordinateSystem: UTM WGS 84; decimalLatitude: 45.198691; decimalLongitude: 9.080746; georeferencedBy: Silvia Stefanelli; georeferenceProtocol: GPS; **Identification:** identifiedBy: Carlo Pesarini; dateIdentified: 2011

##### Ecological interactions

###### Conservation status

Least Concern (European Environment Agency 2013).

##### Distribution

Albania, Austria, Belarus, Belgium, Bosnia and Herzegovina, Bulgaria, Croatia, Czech Republic, Danish mainland, Estonia, Finland, French mainland, Germany, Greek mainland, Hungary, Italian mainland, Latvia, Lithuania, Macedonia, Moldova Republic of, Norwegian mainland, Poland, Romania, Russia Central, Russia East, Russia North, Russia Northwest, Russia South, Sardinia, Slovakia, Slovenia, Spanish mainland, Sweden, Switzerland, Ukraine, Yugoslavia, East Palaearctic, Near East, North Africa, Oriental region (Fauna Europaea 2013).

##### Notes

The species is rare but widespread as are the other species of the genus. The larva develops under the bark of various broadleaves, mainly birch, poplar, willow, and aspen. The adults mostly occur on stacks of dry branches or logs of host plants and fly and run around in sunshine (Hůrka 2005).

#### Xylotrechus stebbingi

(Gahan, 1906)

##### Materials

- **Type status:** Other material. **Occurrence:** recordedBy: Silvia Stefanelli; individualCount: 1; lifeStage: adult; **Taxon:** taxonID: urn:lsid:faunaeur.org:taxname:114526; scientificName: Xylotrechus stebbingi; order: Coleoptera; family: Cerambycidae; genus: Xylotrechus; scientificNameAuthorship: Gahan 1906; **Location:** country: Italy; stateProvince: Pavia; locality: SIC "Boschi Siro Negri e Moriano" - BN1; verbatimElevation: 68 m; verbatimCoordinates: 32T 503258E 5007870N; verbatimCoordinateSystem: UTM WGS 84; decimalLatitude: 45.224312; decimalLongitude: 9.041499; georeferencedBy: Silvia Stefanelli; georeferenceProtocol: GPS; **Identification:** identifiedBy: Carlo Pesarini; dateIdentified: 2011
- **Type status:** Other material. **Occurrence:** recordedBy: Silvia Stefanelli; individualCount: 3; lifeStage: adult; **Taxon:** taxonID: urn:lsid:faunaeur.org:taxname:114526; scientificName: Xylotrechus stebbingi; order: Coleoptera; family: Cerambycidae; genus: Xylotrechus; scientificNameAuthorship: Gahan 1906; **Location:** country: Italy; stateProvince: Pavia; locality: SIC "Boschi Siro Negri e Moriano" - BN21; verbatimElevation: 66 m; verbatimCoordinates: 32T 506342E 5005026N; verbatimCoordinateSystem: UTM WGS 84; decimalLatitude: 45.198691; decimalLongitude: 9.080746; georeferencedBy: Silvia Stefanelli; georeferenceProtocol: GPS; **Identification:** identifiedBy: Carlo Pesarini; dateIdentified: 2011
- **Type status:** Other material. **Occurrence:** recordedBy: Silvia Stefanelli; individualCount: 1; lifeStage: adult; **Taxon:** taxonID: urn:lsid:faunaeur.org:taxname:114526; scientificName: Xylotrechus stebbingi; order: Coleoptera; family: Cerambycidae; genus: Xylotrechus; scientificNameAuthorship: Gahan 1906; **Location:** country: Italy; stateProvince: Pavia; locality: SIC "Boschi Siro Negri e Moriano" - BN5; verbatimElevation: 62 m; verbatimCoordinates: 32T 502886E 5008393N; verbatimCoordinateSystem: UTM WGS 84; decimalLatitude: 45.229029; decimalLongitude: 9.036770; georeferencedBy: Silvia Stefanelli; georeferenceProtocol: GPS; **Identification:** identifiedBy: Carlo Pesarini; dateIdentified: 2011
- **Type status:** Other material. **Occurrence:** recordedBy: Silvia Stefanelli; individualCount: 1; lifeStage: adult; **Taxon:** taxonID: urn:lsid:faunaeur.org:taxname:114526; scientificName: Xylotrechus stebbingi; order: Coleoptera; family: Cerambycidae; genus: Xylotrechus; scientificNameAuthorship: Gahan 1906; **Location:** country: Italy; stateProvince: Pavia; locality: SIC "Boschi Siro Negri e Moriano" - BN10; verbatimElevation: 76 m; verbatimCoordinates: 32T 504479E 5006332N; verbatimCoordinateSystem: UTM WGS 84; decimalLatitude: 45.210461; decimalLongitude: 9.057038; georeferencedBy: Silvia Stefanelli; georeferenceProtocol: GPS; **Identification:** identifiedBy: Carlo Pesarini; dateIdentified: 2011
- **Type status:** Other material. **Occurrence:** recordedBy: Silvia Stefanelli; individualCount: 1; lifeStage: adult; **Taxon:** taxonID: urn:lsid:faunaeur.org:taxname:114526; scientificName: Xylotrechus stebbingi; order: Coleoptera; family: Cerambycidae; genus: Xylotrechus; scientificNameAuthorship: Gahan 1906; **Location:** country: Italy; stateProvince: Pavia; locality: SIC "Boschi di Vaccarizza" - V1; verbatimElevation: 62 m; verbatimCoordinates: 32T 519272E 4999526N; verbatimCoordinateSystem: UTM WGS 84; decimalLatitude: 45.148947; decimalLongitude: 9.245157; georeferencedBy: Silvia Stefanelli; georeferenceProtocol: GPS; **Identification:** identifiedBy: Carlo Pesarini; dateIdentified: 2011

##### Distribution

Crete, French mainland, Greek mainland, Italian mainland, Sardinia, Switzerland, Afro-tropical region, Australian region, Near East, North Africa, Oriental region (Fauna Europaea 2013).

##### Notes

It is an allochthonous species which probably arrived in Europe infesting the wood of mulberry trees. Little is known about its biology (Dioli and Viganò 1990).

#### Cerylon ferrugineum

(Stephens, 1830)

##### Materials

- **Type status:** Other material. **Occurrence:** recordedBy: Silvia Stefanelli; individualCount: 3; lifeStage: adult; **Taxon:** taxonID: urn:lsid:faunaeur.org:taxname:115176; scientificName: Cerylon ferrugineum; order: Coleoptera; family: Cerylonidae; genus: Cerylon; scientificNameAuthorship: Stephens 1830; **Location:** country: Italy; stateProvince: Pavia; locality: SIC "Boschi Siro Negri e Moriano" - BN1; verbatimElevation: 68 m; verbatimCoordinates: 32T 503258E 5007870N; verbatimCoordinateSystem: UTM WGS 84; decimalLatitude: 45.224312; decimalLongitude: 9.041499; georeferencedBy: Silvia Stefanelli; georeferenceProtocol: GPS; **Identification:** identifiedBy: Claudio Canepari; dateIdentified: 2011
- **Type status:** Other material. **Occurrence:** recordedBy: Silvia Stefanelli; individualCount: 2; lifeStage: adult; **Taxon:** taxonID: urn:lsid:faunaeur.org:taxname:115176; scientificName: Cerylon ferrugineum; order: Coleoptera; family: Cerylonidae; genus: Cerylon; scientificNameAuthorship: Stephens 1830; **Location:** country: Italy; stateProvince: Pavia; locality: SIC "Boschi Siro Negri e Moriano" - BN21; verbatimElevation: 66 m; verbatimCoordinates: 32T 506342E 5005026N; verbatimCoordinateSystem: UTM WGS 84; decimalLatitude: 45.198691; decimalLongitude: 9.080746; georeferencedBy: Silvia Stefanelli; georeferenceProtocol: GPS; **Identification:** identifiedBy: Claudio Canepari; dateIdentified: 2011
- **Type status:** Other material. **Occurrence:** recordedBy: Silvia Stefanelli; individualCount: 4; lifeStage: adult; **Taxon:** taxonID: urn:lsid:faunaeur.org:taxname:115176; scientificName: Cerylon ferrugineum; order: Coleoptera; family: Cerylonidae; genus: Cerylon; scientificNameAuthorship: Stephens 1830; **Location:** country: Italy; stateProvince: Pavia; locality: SIC "Boschi Siro Negri e Moriano" - BN5; verbatimElevation: 62 m; verbatimCoordinates: 32T 502886E 5008393N; verbatimCoordinateSystem: UTM WGS 84; decimalLatitude: 45.229029; decimalLongitude: 9.036770; georeferencedBy: Silvia Stefanelli; georeferenceProtocol: GPS; **Identification:** identifiedBy: Claudio Canepari; dateIdentified: 2011

##### Distribution

Austria, Belarus, Belgium, Bosnia and Herzegovina, Britain I., Bulgaria, Corsica, Croatia, Czech Republic, Danish mainland, Estonia, European Turkey, Finland, French mainland, Germany, Greek mainland, Hungary, Ireland, Italian mainland, Latvia, Lithuania, Macedonia, Norwegian mainland, Poland, Romania, Russia Central, Russia East, Russia North, Russia Northwest, Russia South, Slovakia, Slovenia, Spanish mainland, Sweden, Switzerland, The Netherlands, Ukraine, Yugoslavia (Fauna Europaea 2013).

##### Notes

The species lives in ancient forests. The larva develops in the rotten wood of different species of recently dead hardwoods and feeds on decomposing fungi and spores (Alexander and Anderson 2012).

#### Cetonia aurata

(Linneaus, 1761)

Scarabaeus aurata Linneaus, 1761 – Fauna Europaea (2013)

##### Materials

- **Type status:** Other material. **Occurrence:** recordedBy: Silvia Stefanelli; individualCount: 13; lifeStage: adult; **Taxon:** taxonID: urn:lsid:faunaeur.org:taxname:247076; scientificName: Cetonia aurata; order: Coleoptera; family: Cetoniidae; genus: Cetonia; scientificNameAuthorship: Linnaeus 1761; **Location:** country: Italy; stateProvince: Pavia; locality: SIC "Boschi Siro Negri e Moriano" - BN1; verbatimElevation: 68 m; verbatimCoordinates: 32T 503258E 5007870N; verbatimCoordinateSystem: UTM WGS 84; decimalLatitude: 45.224312; decimalLongitude: 9.041499; georeferencedBy: Silvia Stefanelli; georeferenceProtocol: GPS; **Identification:** identifiedBy: Giuseppe Carpaneto; dateIdentified: 2011
- **Type status:** Other material. **Occurrence:** recordedBy: Silvia Stefanelli; individualCount: 2; lifeStage: adult; **Taxon:** taxonID: urn:lsid:faunaeur.org:taxname:247076; scientificName: Cetonia aurata; order: Coleoptera; family: Cetoniidae; genus: Cetonia; scientificNameAuthorship: Linnaeus 1761; **Location:** country: Italy; stateProvince: Pavia; locality: SIC "Boschi Siro Negri e Moriano" - BN21; verbatimElevation: 66 m; verbatimCoordinates: 32T 506342E 5005026N; verbatimCoordinateSystem: UTM WGS 84; decimalLatitude: 45.198691; decimalLongitude: 9.080746; georeferencedBy: Silvia Stefanelli; georeferenceProtocol: GPS; **Identification:** identifiedBy: Giuseppe Carpaneto; dateIdentified: 2011
- **Type status:** Other material. **Occurrence:** recordedBy: Silvia Stefanelli; individualCount: 5; lifeStage: adult; **Taxon:** taxonID: urn:lsid:faunaeur.org:taxname:247076; scientificName: Cetonia aurata; order: Coleoptera; family: Cetoniidae; genus: Cetonia; scientificNameAuthorship: Linnaeus 1761; **Location:** country: Italy; stateProvince: Pavia; locality: SIC "Boschi Siro Negri e Moriano" - BN5; verbatimElevation: 62 m; verbatimCoordinates: 32T 502886E 5008393N; verbatimCoordinateSystem: UTM WGS 84; decimalLatitude: 45.229029; decimalLongitude: 9.036770; georeferencedBy: Silvia Stefanelli; georeferenceProtocol: GPS; **Identification:** identifiedBy: Giuseppe Carpaneto; dateIdentified: 2011
- **Type status:** Other material. **Occurrence:** recordedBy: Silvia Stefanelli; individualCount: 2; lifeStage: adult; **Taxon:** taxonID: urn:lsid:faunaeur.org:taxname:247076; scientificName: Cetonia aurata; order: Coleoptera; family: Cetoniidae; genus: Cetonia; scientificNameAuthorship: Linnaeus 1761; **Location:** country: Italy; stateProvince: Pavia; locality: SIC "Boschi Siro Negri e Moriano" - BN10; verbatimElevation: 76 m; verbatimCoordinates: 32T 504479E 5006332N; verbatimCoordinateSystem: UTM WGS 84; decimalLatitude: 45.210461; decimalLongitude: 9.057038; georeferencedBy: Silvia Stefanelli; georeferenceProtocol: GPS; **Identification:** identifiedBy: Giuseppe Carpaneto; dateIdentified: 2011
- **Type status:** Other material. **Occurrence:** recordedBy: Silvia Stefanelli; individualCount: 1; lifeStage: adult; **Taxon:** taxonID: urn:lsid:faunaeur.org:taxname:247076; scientificName: Cetonia aurata; order: Coleoptera; family: Cetoniidae; genus: Cetonia; scientificNameAuthorship: Linnaeus 1761; **Location:** country: Italy; stateProvince: Pavia; locality: SIC "Boschi di Vaccarizza" - V1; verbatimElevation: 62 m; verbatimCoordinates: 32T 519272E 4999526N; verbatimCoordinateSystem: UTM WGS 84; decimalLatitude: 45.148947; decimalLongitude: 9.245157; georeferencedBy: Silvia Stefanelli; georeferenceProtocol: GPS; **Identification:** identifiedBy: Giuseppe Carpaneto; dateIdentified: 2011
- **Type status:** Other material. **Occurrence:** recordedBy: Silvia Stefanelli; individualCount: 3; lifeStage: adult; **Taxon:** taxonID: urn:lsid:faunaeur.org:taxname:247076; scientificName: Cetonia aurata; order: Coleoptera; family: Cetoniidae; genus: Cetonia; scientificNameAuthorship: Linnaeus 1761; **Location:** country: Italy; stateProvince: Pavia; locality: SIC "Boschi di Vaccarizza" - V2; verbatimElevation: 65 m; verbatimCoordinates: 32T 519868E 4999488N; verbatimCoordinateSystem: UTM WGS 84; decimalLatitude: 45.148589; decimalLongitude: 9.252737; georeferencedBy: Silvia Stefanelli; georeferenceProtocol: GPS; **Identification:** identifiedBy: Giuseppe Carpaneto; dateIdentified: 2011

##### Distribution

Albania, Austria, Belarus, Belgium, Bosnia and Herzegovina, Britain I., Bulgaria, Channel Is., Croatia, Czech Republic, Danish mainland, Estonia, European Turkey, Finland, French mainland, Germany, Greek mainland, Hungary, Ireland, Italian mainland, Kaliningrad Region, Latvia, Liechtenstein, Lithuania, Luxembourg, Macedonia, Malta, Norwegian mainland, Poland, Romania, Russia Central, Russia East, Russia North, Russia Northwest, Russia South, Slovakia, Slovenia, Spanish mainland, Sweden, Switzerland, The Netherlands, Ukraine, Yugoslavia, East Palaearctic, Near East (Fauna Europaea 2013).

##### Notes

The species is very common. The larva develops in the rotten wood of old broadleaves with humus-rich soil. It pupates in late summer and autumn in a cocoon in which the beetle overwinters. The adult appears from April to October on the flowers of various herbs, shrubs, trees, and also sometimes at oozing sap (Hůrka 2005).

#### Oxythyrea funesta

(Poda, 1761)

##### Materials

- **Type status:** Other material. **Occurrence:** recordedBy: Silvia Stefanelli; individualCount: 16; lifeStage: adult; **Taxon:** taxonID: urn:lsid:faunaeur.org:taxname:247005; scientificName: Oxythyrea funesta; order: Coleoptera; family: Cetoniidae; genus: Oxythyrea; scientificNameAuthorship: Poda 1761; **Location:** country: Italy; stateProvince: Pavia; locality: SIC "Boschi Siro Negri e Moriano" - BN1; verbatimElevation: 68 m; verbatimCoordinates: 32T 503258E 5007870N; verbatimCoordinateSystem: UTM WGS 84; decimalLatitude: 45.224312; decimalLongitude: 9.041499; georeferencedBy: Silvia Stefanelli; georeferenceProtocol: GPS; **Identification:** identifiedBy: Giuseppe Carpaneto; dateIdentified: 2011
- **Type status:** Other material. **Occurrence:** recordedBy: Silvia Stefanelli; individualCount: 2; lifeStage: adult; **Taxon:** taxonID: urn:lsid:faunaeur.org:taxname:247005; scientificName: Oxythyrea funesta; order: Coleoptera; family: Cetoniidae; genus: Oxythyrea; scientificNameAuthorship: Poda 1761; **Location:** country: Italy; stateProvince: Pavia; locality: SIC "Boschi Siro Negri e Moriano" - BN21; verbatimElevation: 66 m; verbatimCoordinates: 32T 506342E 5005026N; verbatimCoordinateSystem: UTM WGS 84; decimalLatitude: 45.198691; decimalLongitude: 9.080746; georeferencedBy: Silvia Stefanelli; georeferenceProtocol: GPS; **Identification:** identifiedBy: Giuseppe Carpaneto; dateIdentified: 2011
- **Type status:** Other material. **Occurrence:** recordedBy: Silvia Stefanelli; individualCount: 4; lifeStage: adult; **Taxon:** taxonID: urn:lsid:faunaeur.org:taxname:247005; scientificName: Oxythyrea funesta; order: Coleoptera; family: Cetoniidae; genus: Oxythyrea; scientificNameAuthorship: Poda 1761; **Location:** country: Italy; stateProvince: Pavia; locality: SIC "Boschi Siro Negri e Moriano" - BN5; verbatimElevation: 62 m; verbatimCoordinates: 32T 502886E 5008393N; verbatimCoordinateSystem: UTM WGS 84; decimalLatitude: 45.229029; decimalLongitude: 9.036770; georeferencedBy: Silvia Stefanelli; georeferenceProtocol: GPS; **Identification:** identifiedBy: Giuseppe Carpaneto; dateIdentified: 2011

##### Distribution

Albania, Andorra, Austria, Balearic Is., Belarus, Belgium, Bosnia and Herzegovina, Bulgaria, Canary Is., Channel Is., Corsica, Croatia, Czech Republic, European Turkey, French mainland, Germany, Greek mainland, Hungary, Italian mainland, Latvia, Lithuania, Luxembourg, Macedonia, Malta, North Aegean Is., Poland, Portuguese mainland, Romania, Russia Central, Russia East, Russia South, Sardinia, Sicily, Slovakia, Slovenia, Spanish mainland, Switzerland, The Netherlands, Ukraine, Yugoslavia, Near East, North Africa (Fauna Europaea 2013).

##### Notes

It is a thermophilous species. The adult appears from May to July on the flowers of various herbs and shrubs (Hůrka 2005).

#### Potosia cuprea

(Fabricius, 1775)

##### Materials

- **Type status:** Other material. **Occurrence:** recordedBy: Silvia Stefanelli; individualCount: 1; lifeStage: adult; **Taxon:** scientificName: Potosia cuprea; order: Coleoptera; family: Cetoniidae; genus: Potosia; scientificNameAuthorship: Fabricius 1775; **Location:** country: Italy; stateProvince: Pavia; locality: SIC "Boschi Siro Negri e Moriano" - BN1; verbatimElevation: 68 m; verbatimCoordinates: 32T 503258E 5007870N; verbatimCoordinateSystem: UTM WGS 84; decimalLatitude: 45.224312; decimalLongitude: 9.041499; georeferencedBy: Silvia Stefanelli; georeferenceProtocol: GPS; **Identification:** identifiedBy: Giuseppe Carpaneto; dateIdentified: 2011
- **Type status:** Other material. **Occurrence:** recordedBy: Silvia Stefanelli; individualCount: 3; lifeStage: adult; **Taxon:** scientificName: Potosia cuprea; order: Coleoptera; family: Cetoniidae; genus: Potosia; scientificNameAuthorship: Fabricius 1775; **Location:** country: Italy; stateProvince: Pavia; locality: SIC "Boschi Siro Negri e Moriano" - BN21; verbatimElevation: 66 m; verbatimCoordinates: 32T 506342E 5005026N; verbatimCoordinateSystem: UTM WGS 84; decimalLatitude: 45.198691; decimalLongitude: 9.080746; georeferencedBy: Silvia Stefanelli; georeferenceProtocol: GPS; **Identification:** identifiedBy: Giuseppe Carpaneto; dateIdentified: 2011

##### Notes

It is a common, hardy species with variable coloration. The larva which often occurs at the edges of ant hills of *Formica rufa* group feeds on nest material and various wood debris. Females lay eggs near the anthills and larval development takes two years. The adult appears from May to July on flowers and ripe fruits (Hůrka 2005).

#### Tropinota (Epicometis) hirta

(Poda, 1761)

##### Materials

- **Type status:** Other material. **Occurrence:** recordedBy: Silvia Stefanelli; individualCount: 1; lifeStage: adult; **Taxon:** taxonID: urn:lsid:faunaeur.org:taxname:247090; scientificName: Tropinota hirta; order: Coleoptera; family: Cetoniidae; genus: Tropinota; scientificNameAuthorship: Poda 1761; **Location:** country: Italy; stateProvince: Pavia; locality: SIC "Boschi Siro Negri e Moriano" - BN1; verbatimElevation: 68 m; verbatimCoordinates: 32T 503258E 5007870N; verbatimCoordinateSystem: UTM WGS 84; decimalLatitude: 45.224312; decimalLongitude: 9.041499; georeferencedBy: Silvia Stefanelli; georeferenceProtocol: GPS; **Identification:** identifiedBy: Giuseppe Carpaneto; dateIdentified: 2011
- **Type status:** Other material. **Occurrence:** recordedBy: Silvia Stefanelli; individualCount: 1; lifeStage: adult; **Taxon:** taxonID: urn:lsid:faunaeur.org:taxname:247090; scientificName: Tropinota hirta; order: Coleoptera; family: Cetoniidae; genus: Tropinota; scientificNameAuthorship: Poda 1761; **Location:** country: Italy; stateProvince: Pavia; locality: SIC "Boschi Siro Negri e Moriano" - BN21; verbatimElevation: 66 m; verbatimCoordinates: 32T 506342E 5005026N; verbatimCoordinateSystem: UTM WGS 84; decimalLatitude: 45.198691; decimalLongitude: 9.080746; georeferencedBy: Silvia Stefanelli; georeferenceProtocol: GPS; **Identification:** identifiedBy: Giuseppe Carpaneto; dateIdentified: 2011

##### Distribution

Albania, Andorra, Austria, Balearic Is., Belarus, Belgium, Bosnia and Herzegovina, Bulgaria, Channel Is., Corsica, Crete, Croatia, Cyclades Is., Cyprus, Czech Republic, Dodecanese Is., European Turkey, French mainland, Germany, Greek mainland, Hungary, Italian mainland, Kaliningrad Region, Latvia, Lithuania, Luxembourg, Macedonia, Moldova Republic of, Poland, Romania, Russia East, Russia South, Sardinia, Sicily, Slovakia, Slovenia, Spanish mainland, Switzerland, The Netherlands, Ukraine, Yugoslavia, Near East, North Africa (Fauna Europaea 2013).

##### Notes

This is an univoltine species. The larva feeds on decaying plant matter and roots in the soil. The adult appears at the end of March mostly on yellow flowers and feeds on pollen (Hůrka 2005).

#### Valgus hemipterus

(Linneaus, 1758)

##### Materials

- **Type status:** Other material. **Occurrence:** recordedBy: Silvia Stefanelli; individualCount: 64; lifeStage: adult; **Taxon:** taxonID: urn:lsid:faunaeur.org:taxname:246966; scientificName: Valgus hemipterus; order: Coleoptera; family: Cetoniidae; genus: Valgus; scientificNameAuthorship: Linnaeus 1758; **Location:** country: Italy; stateProvince: Pavia; locality: SIC "Boschi Siro Negri e Moriano" - BN1; verbatimElevation: 68 m; verbatimCoordinates: 32T 503258E 5007870N; verbatimCoordinateSystem: UTM WGS 84; decimalLatitude: 45.224312; decimalLongitude: 9.041499; georeferencedBy: Silvia Stefanelli; georeferenceProtocol: GPS; **Identification:** identifiedBy: Giuseppe Carpaneto; dateIdentified: 2011
- **Type status:** Other material. **Occurrence:** recordedBy: Silvia Stefanelli; individualCount: 108; lifeStage: adult; **Taxon:** taxonID: urn:lsid:faunaeur.org:taxname:246966; scientificName: Valgus hemipterus; order: Coleoptera; family: Cetoniidae; genus: Valgus; scientificNameAuthorship: Linnaeus 1758; **Location:** country: Italy; stateProvince: Pavia; locality: SIC "Boschi Siro Negri e Moriano" - BN21; verbatimElevation: 66 m; verbatimCoordinates: 32T 506342E 5005026N; verbatimCoordinateSystem: UTM WGS 84; decimalLatitude: 45.198691; decimalLongitude: 9.080746; georeferencedBy: Silvia Stefanelli; georeferenceProtocol: GPS; **Identification:** identifiedBy: Giuseppe Carpaneto; dateIdentified: 2011
- **Type status:** Other material. **Occurrence:** recordedBy: Silvia Stefanelli; individualCount: 22; lifeStage: adult; **Taxon:** taxonID: urn:lsid:faunaeur.org:taxname:246966; scientificName: Valgus hemipterus; order: Coleoptera; family: Cetoniidae; genus: Valgus; scientificNameAuthorship: Linnaeus 1758; **Location:** country: Italy; stateProvince: Pavia; locality: SIC "Boschi Siro Negri e Moriano" - BN5; verbatimElevation: 62 m; verbatimCoordinates: 32T 502886E 5008393N; verbatimCoordinateSystem: UTM WGS 84; decimalLatitude: 45.229029; decimalLongitude: 9.036770; georeferencedBy: Silvia Stefanelli; georeferenceProtocol: GPS; **Identification:** identifiedBy: Giuseppe Carpaneto; dateIdentified: 2011
- **Type status:** Other material. **Occurrence:** recordedBy: Silvia Stefanelli; individualCount: 30; lifeStage: adult; **Taxon:** taxonID: urn:lsid:faunaeur.org:taxname:246966; scientificName: Valgus hemipterus; order: Coleoptera; family: Cetoniidae; genus: Valgus; scientificNameAuthorship: Linnaeus 1758; **Location:** country: Italy; stateProvince: Pavia; locality: SIC "Boschi Siro Negri e Moriano" - BN10; verbatimElevation: 76 m; verbatimCoordinates: 32T 504479E 5006332N; verbatimCoordinateSystem: UTM WGS 84; decimalLatitude: 45.210461; decimalLongitude: 9.057038; georeferencedBy: Silvia Stefanelli; georeferenceProtocol: GPS; **Identification:** identifiedBy: Giuseppe Carpaneto; dateIdentified: 2011
- **Type status:** Other material. **Occurrence:** recordedBy: Silvia Stefanelli; individualCount: 22; lifeStage: adult; **Taxon:** taxonID: urn:lsid:faunaeur.org:taxname:246966; scientificName: Valgus hemipterus; order: Coleoptera; family: Cetoniidae; genus: Valgus; scientificNameAuthorship: Linnaeus 1758; **Location:** country: Italy; stateProvince: Pavia; locality: SIC "Boschi di Vaccarizza" - V1; verbatimElevation: 62 m; verbatimCoordinates: 32T 519272E 4999526N; verbatimCoordinateSystem: UTM WGS 84; decimalLatitude: 45.148947; decimalLongitude: 9.245157; georeferencedBy: Silvia Stefanelli; georeferenceProtocol: GPS; **Identification:** identifiedBy: Giuseppe Carpaneto; dateIdentified: 2011
- **Type status:** Other material. **Occurrence:** recordedBy: Silvia Stefanelli; individualCount: 15; lifeStage: adult; **Taxon:** taxonID: urn:lsid:faunaeur.org:taxname:246966; scientificName: Valgus hemipterus; order: Coleoptera; family: Cetoniidae; genus: Valgus; scientificNameAuthorship: Linnaeus 1758; **Location:** country: Italy; stateProvince: Pavia; locality: SIC "Boschi di Vaccarizza" - V2; verbatimElevation: 65 m; verbatimCoordinates: 32T 519868E 4999488N; verbatimCoordinateSystem: UTM WGS 84; decimalLatitude: 45.148589; decimalLongitude: 9.252737; georeferencedBy: Silvia Stefanelli; georeferenceProtocol: GPS; **Identification:** identifiedBy: Giuseppe Carpaneto; dateIdentified: 2011

##### Ecological interactions

###### Conservation status

Least Concern (European Environment Agency 2013).

##### Distribution

Albania, Austria, Balearic Is., Belarus, Belgium, Bosnia and Herzegovina, Bulgaria, Corsica, Crete, Croatia, Cyprus, Czech Republic, Danish mainland, European Turkey, French mainland, Germany, Greek mainland, Hungary, Italian mainland, Latvia, Lithuania, Luxembourg, Macedonia, Poland, Portuguese mainland, Romania, Russia Central, Russia Northwest, Russia South, Sardinia, Sicily, Slovakia, Slovenia, Spanish mainland, Switzerland, The Netherlands, Ukraine, Yugoslavia, Near East, Nearctic region, North Africa (Fauna Europaea 2013).

##### Notes

The larva develops in the decaying wood of dead broadleaves for one year. The adult appears from the end of April to the end of June on flowers in warmer areas (Hůrka 2005).

#### Phloeophagus lignarius

(Marsham, 1802)

Rhyncolus sulcirostris Thomson, 1894 – Fauna Europaea (2013)
Cossonus cylindrirostris Olivier, 1807 – Fauna Europaea (2013)
Rhyncolus latirostris Thomson, 1886 – Fauna Europaea (2013)

##### Materials

- **Type status:** Other material. **Occurrence:** recordedBy: Silvia Stefanelli; individualCount: 1; lifeStage: adult; **Taxon:** taxonID: urn:lsid:faunaeur.org:taxname:248036; scientificName: Phloeophagus lignarius; order: Coleoptera; family: Curculionidae; genus: Phloeophagus; scientificNameAuthorship: Marsham 1802; **Location:** country: Italy; stateProvince: Pavia; locality: SIC "Boschi Siro Negri e Moriano" - BN21; verbatimElevation: 66 m; verbatimCoordinates: 32T 506342E 5005026N; verbatimCoordinateSystem: UTM WGS 84; decimalLatitude: 45.198691; decimalLongitude: 9.080746; georeferencedBy: Silvia Stefanelli; georeferenceProtocol: GPS; **Identification:** identifiedBy: Enzo Colonnelli; dateIdentified: 2011
- **Type status:** Other material. **Occurrence:** recordedBy: Silvia Stefanelli; individualCount: 1; lifeStage: adult; **Taxon:** taxonID: urn:lsid:faunaeur.org:taxname:248036; scientificName: Phloeophagus lignarius; order: Coleoptera; family: Curculionidae; genus: Phloeophagus; scientificNameAuthorship: Marsham 1802; **Location:** country: Italy; stateProvince: Pavia; locality: SIC "Boschi Siro Negri e Moriano" - BN10; verbatimElevation: 76 m; verbatimCoordinates: 32T 504479E 5006332N; verbatimCoordinateSystem: UTM WGS 84; decimalLatitude: 45.210461; decimalLongitude: 9.057038; georeferencedBy: Silvia Stefanelli; georeferenceProtocol: GPS; **Identification:** identifiedBy: Enzo Colonnelli; dateIdentified: 2011

##### Distribution

Albania, Andorra, Austria, Balearic Is., Bosnia and Herzegovina, Britain I., Bulgaria, Czech Republic, Danish mainland, Estonia, Finland, French mainland, Germany, Gibraltar, Hungary, Italian mainland, Latvia, Norwegian mainland, Poland, Portuguese mainland, Romania, Russia South, Slovakia, Spanish mainland, Sweden, Switzerland, The Netherlands, Ukraine (Fauna Europaea 2013).

##### Notes

The species develops in the decayed heartwood of beech, hawthorn, and ash (Alexander 2002).

#### Dryophthorus corticalis

(Paykull, 1792)

Curculio corticalis (Paykull, 1792) – Fauna Europaea (2013)

##### Materials

- **Type status:** Other material. **Occurrence:** recordedBy: Silvia Stefanelli; individualCount: 2; lifeStage: adult; **Taxon:** taxonID: urn:lsid:faunaeur.org:taxname:257317; scientificName: Dryophthorus corticalis; order: Coleoptera; family: Dryophthoridae; genus: Dryophthorus; scientificNameAuthorship: Paykull 1792; **Location:** country: Italy; stateProvince: Pavia; locality: SIC "Boschi Siro Negri e Moriano" - BN21; verbatimElevation: 66 m; verbatimCoordinates: 32T 506342E 5005026N; verbatimCoordinateSystem: UTM WGS 84; decimalLatitude: 45.198691; decimalLongitude: 9.080746; georeferencedBy: Silvia Stefanelli; georeferenceProtocol: GPS; **Identification:** identifiedBy: Enzo Colonnelli; dateIdentified: 2011
- **Type status:** Other material. **Occurrence:** recordedBy: Silvia Stefanelli; individualCount: 1; lifeStage: adult; **Taxon:** taxonID: urn:lsid:faunaeur.org:taxname:257317; scientificName: Dryophthorus corticalis; order: Coleoptera; family: Dryophthoridae; genus: Dryophthorus; scientificNameAuthorship: Paykull 1792; **Location:** country: Italy; stateProvince: Pavia; locality: SIC "Boschi Siro Negri e Moriano" - BN5; verbatimElevation: 62 m; verbatimCoordinates: 32T 502886E 5008393N; verbatimCoordinateSystem: UTM WGS 84; decimalLatitude: 45.229029; decimalLongitude: 9.036770; georeferencedBy: Silvia Stefanelli; georeferenceProtocol: GPS; **Identification:** identifiedBy: Enzo Colonnelli; dateIdentified: 2011
- **Type status:** Other material. **Occurrence:** recordedBy: Silvia Stefanelli; individualCount: 28; lifeStage: adult; **Taxon:** taxonID: urn:lsid:faunaeur.org:taxname:257317; scientificName: Dryophthorus corticalis; order: Coleoptera; family: Dryophthoridae; genus: Dryophthorus; scientificNameAuthorship: Paykull 1792; **Location:** country: Italy; stateProvince: Pavia; locality: SIC "Boschi Siro Negri e Moriano" - BN10; verbatimElevation: 76 m; verbatimCoordinates: 32T 504479E 5006332N; verbatimCoordinateSystem: UTM WGS 84; decimalLatitude: 45.210461; decimalLongitude: 9.057038; georeferencedBy: Silvia Stefanelli; georeferenceProtocol: GPS; **Identification:** identifiedBy: Enzo Colonnelli; dateIdentified: 2011

##### Distribution

Austria, Britain I., Bulgaria, Corsica, Czech Republic, Danish mainland, Estonia, Finland, French mainland, Germany, Hungary, Italian mainland, Kaliningrad Region, Latvia, Lithuania, Norwegian mainland, Poland, Russia Northwest, Sardinia, Sicily, Slovakia, Sweden, Switzerland, The Netherlands, Ukraine (Fauna Europaea 2013).

##### Notes

The species inhabits the old relict forests of broadleaves and conifers throughout Europe. Despite their wide distribution, this species is pretty rare probably because of its hidden lifestyle and difficulty to collect. The larva develops in the hard wood of oaks and also in beeches and is often associated with the ant *Lasius brunneus* (Alexander 2002, Pešić 2011).

#### Ampedus (Ampedus) cinnaberinus

(Eschscholtz, 1829)

Elater angusticollis Heyden, 1886 – Fauna Europaea (2013)
Ampedus lythropterus Germar, 1844 – Fauna Europaea (2013)

##### Materials

- **Type status:** Other material. **Occurrence:** recordedBy: Silvia Stefanelli; individualCount: 2; lifeStage: adult; **Taxon:** taxonID: urn:lsid:faunaeur.org:taxname:235494; scientificName: Ampedus cinnaberinus; order: Coleoptera; family: Elateridae; genus: Ampedus; scientificNameAuthorship: Eschscholtz 1829; **Location:** country: Italy; stateProvince: Pavia; locality: SIC "Boschi Siro Negri e Moriano" - BN5; verbatimElevation: 62 m; verbatimCoordinates: 32T 502886E 5008393N; verbatimCoordinateSystem: UTM WGS 84; decimalLatitude: 45.229029; decimalLongitude: 9.036770; georeferencedBy: Silvia Stefanelli; georeferenceProtocol: GPS; **Identification:** identifiedBy: Giuseppe Platia; dateIdentified: 2011

##### Ecological interactions

###### Conservation status

Least Concern (European Environment Agency 2013).

##### Distribution

Austria, Balearic Is., Belarus, Belgium, Bosnia and Herzegovina, Britain I., Bulgaria, Croatia, Czech Republic, Danish mainland, Estonia, Finland, French mainland, Germany, Greek mainland, Hungary, Italian mainland, Kaliningrad Region, Latvia, Lithuania, Luxembourg, Moldova Republic of, Norwegian mainland, Poland, Portuguese mainland, Romania, Russia Central, Russia East, Russia North, Russia Northwest, Russia South, Sicily, Slovakia, Slovenia, Spanish mainland, Sweden, Switzerland, The Netherlands, Ukraine, East Palaearctic, Near East, North Africa (Fauna Europaea 2013).

##### Notes

The species is principally associated with ancient oak forests. The larva develops in the dead timber of various broadleaves mainly in heart-rot, but also under the bark of rotten limbs. It is a predator of other beetle larvae, often *Dorcus* sp. (Alexander 2002).

#### Ampedus (Ampedus) pomonae

(Stephens, 1830)

##### Materials

- **Type status:** Other material. **Occurrence:** recordedBy: Silvia Stefanelli; individualCount: 2; lifeStage: adult; **Taxon:** taxonID: urn:lsid:faunaeur.org:taxname:235531; scientificName: Ampedus pomonae; order: Coleoptera; family: Elateridae; genus: Ampedus; scientificNameAuthorship: Stephens 1830; **Location:** country: Italy; stateProvince: Pavia; locality: SIC "Boschi Siro Negri e Moriano" - BN1; verbatimElevation: 68 m; verbatimCoordinates: 32T 503258E 5007870N; verbatimCoordinateSystem: UTM WGS 84; decimalLatitude: 45.224312; decimalLongitude: 9.041499; georeferencedBy: Silvia Stefanelli; georeferenceProtocol: GPS; **Identification:** identifiedBy: Giuseppe Platia; dateIdentified: 2011
- **Type status:** Other material. **Occurrence:** recordedBy: Silvia Stefanelli; individualCount: 1; lifeStage: adult; **Taxon:** taxonID: urn:lsid:faunaeur.org:taxname:235531; scientificName: Ampedus pomonae; order: Coleoptera; family: Elateridae; genus: Ampedus; scientificNameAuthorship: Stephens 1830; **Location:** country: Italy; stateProvince: Pavia; locality: SIC "Boschi di Vaccarizza" - V1; verbatimElevation: 62 m; verbatimCoordinates: 32T 519272E 4999526N; verbatimCoordinateSystem: UTM WGS 84; decimalLatitude: 45.148947; decimalLongitude: 9.245157; georeferencedBy: Silvia Stefanelli; georeferenceProtocol: GPS; **Identification:** identifiedBy: Giuseppe Platia; dateIdentified: 2011

##### Ecological interactions

###### Conservation status

Least Concern (European Environment Agency 2013).

##### Distribution

Austria, Belarus, Belgium, Bosnia and Herzegovina, Bulgaria, Croatia, Czech Republic, Danish mainland, Estonia, Finland, French mainland, Germany, Hungary, Ireland, Italian mainland, Kaliningrad Region, Latvia, Lithuania, Luxembourg, Moldova Republic of, Norwegian mainland, Poland, Portuguese mainland, Romania, Russia Central, Russia East, Russia North, Russia Northwest, Russia South, Slovakia, Slovenia, Spanish mainland, Sweden, The Netherlands, Ukraine, Yugoslavia, East Palaearctic, Near East (Fauna Europaea 2013).

##### Notes

This species is typical of wetlands, particularly the riparian forests of the Lombardy plains. The larva develops in the stumps of different species including both broadleaves and conifers (Platia 1994).

#### Ampedus (Ampedus) pomorum

(Herbst, 1784)

Ampedus brigittae Bouwer, 1980 – Fauna Europaea (2013)
Ampedus robustus Bouwer, 1980 – Fauna Europaea (2013)

##### Materials

- **Type status:** Other material. **Occurrence:** recordedBy: Silvia Stefanelli; individualCount: 3; lifeStage: adult; **Taxon:** taxonID: urn:lsid:faunaeur.org:taxname:235532; scientificName: Ampedus pomorum; order: Coleoptera; family: Elateridae; genus: Ampedus; scientificNameAuthorship: Herbst 1784; **Location:** country: Italy; stateProvince: Pavia; locality: SIC "Boschi Siro Negri e Moriano" - BN1; verbatimElevation: 68 m; verbatimCoordinates: 32T 503258E 5007870N; verbatimCoordinateSystem: UTM WGS 84; decimalLatitude: 45.224312; decimalLongitude: 9.041499; georeferencedBy: Silvia Stefanelli; georeferenceProtocol: GPS; **Identification:** identifiedBy: Giuseppe Platia; dateIdentified: 2011
- **Type status:** Other material. **Occurrence:** recordedBy: Silvia Stefanelli; individualCount: 3; lifeStage: adult; **Taxon:** taxonID: urn:lsid:faunaeur.org:taxname:235532; scientificName: Ampedus pomorum; order: Coleoptera; family: Elateridae; genus: Ampedus; scientificNameAuthorship: Herbst 1784; **Location:** country: Italy; stateProvince: Pavia; locality: SIC "Boschi Siro Negri e Moriano" - BN10; verbatimElevation: 76 m; verbatimCoordinates: 32T 504479E 5006332N; verbatimCoordinateSystem: UTM WGS 84; decimalLatitude: 45.210461; decimalLongitude: 9.057038; georeferencedBy: Silvia Stefanelli; georeferenceProtocol: GPS; **Identification:** identifiedBy: Giuseppe Platia; dateIdentified: 2011
- **Type status:** Other material. **Occurrence:** recordedBy: Silvia Stefanelli; individualCount: 2; lifeStage: adult; **Taxon:** taxonID: urn:lsid:faunaeur.org:taxname:235532; scientificName: Ampedus pomorum; order: Coleoptera; family: Elateridae; genus: Ampedus; scientificNameAuthorship: Herbst 1784; **Location:** country: Italy; stateProvince: Pavia; locality: SIC "Boschi di Vaccarizza" - V1; verbatimElevation: 62 m; verbatimCoordinates: 32T 519272E 4999526N; verbatimCoordinateSystem: UTM WGS 84; decimalLatitude: 45.148947; decimalLongitude: 9.245157; georeferencedBy: Silvia Stefanelli; georeferenceProtocol: GPS; **Identification:** identifiedBy: Giuseppe Platia; dateIdentified: 2011
- **Type status:** Other material. **Occurrence:** recordedBy: Silvia Stefanelli; individualCount: 2; lifeStage: adult; **Taxon:** taxonID: urn:lsid:faunaeur.org:taxname:235532; scientificName: Ampedus pomorum; order: Coleoptera; family: Elateridae; genus: Ampedus; scientificNameAuthorship: Herbst 1784; **Location:** country: Italy; stateProvince: Pavia; locality: SIC "Boschi di Vaccarizza" - V2; verbatimElevation: 65 m; verbatimCoordinates: 32T 519868E 4999488N; verbatimCoordinateSystem: UTM WGS 84; decimalLatitude: 45.148589; decimalLongitude: 9.252737; georeferencedBy: Silvia Stefanelli; georeferenceProtocol: GPS; **Identification:** identifiedBy: Giuseppe Platia; dateIdentified: 2011

##### Ecological interactions

###### Conservation status

Least Concern (European Environment Agency 2013).

##### Distribution

Albania, Austria, Belarus, Belgium, Bosnia and Herzegovina, Britain I., Bulgaria, Croatia, Czech Republic, Danish mainland, Estonia, Finland, French mainland, Germany, Greek mainland, Hungary, Ireland, Italian mainland, Kaliningrad Region, Latvia, Liechtenstein, Lithuania, Luxembourg, Macedonia, Moldova Republic of, Northern Ireland, Norwegian mainland, Poland, Romania, Russia Central, Russia East, Russia North, Russia Northwest, Russia South, Slovakia, Slovenia, Spanish mainland, Sweden, Switzerland, The Netherlands, Ukraine, Yugoslavia, East Palaearctic, Near East (Fauna Europaea 2013).

##### Notes

The species is primarily associated with ancient wood pastures. Larva develops in the decayed timber of oak, birch, pine, and probably other trees. It pupates at the end of the season and hibernates as adult. The adult is active from May to June (Alexander 2002).

#### Ampedus (Ampedus) sanguinolentus

(Schrank, 1776)

Elater sanguinolentus Schrank, 1776

##### Materials

- **Type status:** Other material. **Occurrence:** recordedBy: Silvia Stefanelli; individualCount: 11; lifeStage: adult; **Taxon:** taxonID: urn:lsid:faunaeur.org:taxname:235544; scientificName: Ampedus sanguinolentus; order: Coleoptera; family: Elateridae; genus: Ampedus; scientificNameAuthorship: Schrank 1776; **Location:** country: Italy; stateProvince: Pavia; locality: SIC "Boschi Siro Negri e Moriano" - BN1; verbatimElevation: 68 m; verbatimCoordinates: 32T 503258E 5007870N; verbatimCoordinateSystem: UTM WGS 84; decimalLatitude: 45.224312; decimalLongitude: 9.041499; georeferencedBy: Silvia Stefanelli; georeferenceProtocol: GPS; **Identification:** identifiedBy: Giuseppe Platia; dateIdentified: 2011
- **Type status:** Other material. **Occurrence:** recordedBy: Silvia Stefanelli; individualCount: 1; lifeStage: adult; **Taxon:** taxonID: urn:lsid:faunaeur.org:taxname:235544; scientificName: Ampedus sanguinolentus; order: Coleoptera; family: Elateridae; genus: Ampedus; scientificNameAuthorship: Schrank 1776; **Location:** country: Italy; stateProvince: Pavia; locality: SIC "Boschi Siro Negri e Moriano" - BN5; verbatimElevation: 62 m; verbatimCoordinates: 32T 502886E 5008393N; verbatimCoordinateSystem: UTM WGS 84; decimalLatitude: 45.229029; decimalLongitude: 9.036770; georeferencedBy: Silvia Stefanelli; georeferenceProtocol: GPS; **Identification:** identifiedBy: Giuseppe Platia; dateIdentified: 2011
- **Type status:** Other material. **Occurrence:** recordedBy: Silvia Stefanelli; individualCount: 1; lifeStage: adult; **Taxon:** taxonID: urn:lsid:faunaeur.org:taxname:235544; scientificName: Ampedus sanguinolentus; order: Coleoptera; family: Elateridae; genus: Ampedus; scientificNameAuthorship: Schrank 1776; **Location:** country: Italy; stateProvince: Pavia; locality: SIC "Boschi di Vaccarizza" - V2; verbatimElevation: 65 m; verbatimCoordinates: 32T 519868E 4999488N; verbatimCoordinateSystem: UTM WGS 84; decimalLatitude: 45.148589; decimalLongitude: 9.252737; georeferencedBy: Silvia Stefanelli; georeferenceProtocol: GPS; **Identification:** identifiedBy: Giuseppe Platia; dateIdentified: 2011

##### Ecological interactions

###### Conservation status

Least Concern (European Environment Agency 2013).

##### Distribution

Albania, Austria, Belarus, Belgium, Bosnia and Herzegovina, Britain I., Bulgaria, Crete, Croatia, Czech Republic, Danish mainland, Estonia, Finland, French mainland, Germany, Greek mainland, Hungary, Italian mainland, Kaliningrad Region, Latvia, Liechtenstein, Lithuania, Luxembourg, Macedonia, Moldova Republic of, Norwegian mainland, Poland, Portuguese mainland, Romania, Russia Central, Russia East, Russia North, Russia Northwest, Russia South, Sicily, Slovakia, Slovenia, Spanish mainland, Sweden, Switzerland, The Netherlands, Ukraine, Yugoslavia, Near East (Fauna Europaea 2013).

##### Notes

It is one of the most common species associated with ancient forests and wetlands. The species lives on the banks of rivers and swamps where the larva develops in many deciduous species and in the decay of logs, stumps, and branches. It pupates at end of the season and hibernates as adult (Alexander 2002, Platia 1994).

#### Calambus bipustulatus

(Linneaus, 1767)

##### Materials

- **Type status:** Other material. **Occurrence:** recordedBy: Silvia Stefanelli; individualCount: 1; lifeStage: adult; **Taxon:** taxonID: urn:lsid:faunaeur.org:taxname:235800; scientificName: Calambus bipustulatus; order: Coleoptera; family: Elateridae; genus: Calambus; nomenclaturalCode: Linnaeus 1767; **Location:** country: Italy; stateProvince: Pavia; locality: SIC "Boschi Siro Negri e Moriano" - BN10; verbatimElevation: 76 m; verbatimCoordinates: 32T 504479E 5006332N; verbatimCoordinateSystem: UTM WGS 84; decimalLatitude: 45.210461; decimalLongitude: 9.057038; georeferencedBy: Silvia Stefanelli; georeferenceProtocol: GPS; **Identification:** identifiedBy: Giuseppe Platia; dateIdentified: 2011

##### Ecological interactions

###### Conservation status

Least Concern (European Environment Agency 2013).

##### Distribution

Austria, Belgium, Bosnia and Herzegovina, Britain I., Bulgaria, Czech Republic, Danish mainland, French mainland, Germany, Hungary, Ireland, Italian mainland, Kaliningrad Region, Latvia, Lithuania, Luxembourg, Norwegian mainland, Poland, Romania, Russia Central, Russia East, Russia Northwest, Slovakia, Slovenia, Spanish mainland, Sweden, Switzerland, The Netherlands, Ukraine, Yugoslavia, East Palaearctic (Fauna Europaea 2013).

##### Notes

The species is found in the winter under the bark of old oak trees such as lindens, elms, poplars, and mulberry trees. The larva is a predator living in the soft rotten wood of stumps and trunks on the same plant as the adult, especially oaks (Platia 1994, Alexander 2002).

#### Cardiophorus (Cardiophorus) anticus

(Erichson, 1840)

##### Materials

- **Type status:** Other material. **Occurrence:** recordedBy: Silvia Stefanelli; individualCount: 2; lifeStage: adult; **Taxon:** taxonID: urn:lsid:faunaeur.org:taxname:235810; scientificName: Cardiophorus anticus; order: Coleoptera; family: Elateridae; genus: Cardiophorus; scientificNameAuthorship: Erichson 1840; **Location:** country: Italy; stateProvince: Pavia; locality: SIC "Boschi Siro Negri e Moriano" - BN1; verbatimElevation: 68 m; verbatimCoordinates: 32T 503258E 5007870N; verbatimCoordinateSystem: UTM WGS 84; decimalLatitude: 45.224312; decimalLongitude: 9.041499; georeferencedBy: Silvia Stefanelli; georeferenceProtocol: GPS; **Identification:** identifiedBy: Giuseppe Platia; dateIdentified: 2011
- **Type status:** Other material. **Occurrence:** recordedBy: Silvia Stefanelli; individualCount: 1; lifeStage: adult; **Taxon:** taxonID: urn:lsid:faunaeur.org:taxname:235810; scientificName: Cardiophorus anticus; order: Coleoptera; family: Elateridae; genus: Cardiophorus; scientificNameAuthorship: Erichson 1840; **Location:** country: Italy; stateProvince: Pavia; locality: SIC "Boschi Siro Negri e Moriano" - BN5; verbatimElevation: 62 m; verbatimCoordinates: 32T 502886E 5008393N; verbatimCoordinateSystem: UTM WGS 84; decimalLatitude: 45.229029; decimalLongitude: 9.036770; georeferencedBy: Silvia Stefanelli; georeferenceProtocol: GPS; **Identification:** identifiedBy: Giuseppe Platia; dateIdentified: 2011
- **Type status:** Other material. **Occurrence:** recordedBy: Silvia Stefanelli; individualCount: 3; lifeStage: adult; **Taxon:** taxonID: urn:lsid:faunaeur.org:taxname:235810; scientificName: Cardiophorus anticus; order: Coleoptera; family: Elateridae; genus: Cardiophorus; scientificNameAuthorship: Erichson 1840; **Location:** country: Italy; stateProvince: Pavia; locality: SIC "Boschi Siro Negri e Moriano" - BN10; verbatimElevation: 76 m; verbatimCoordinates: 32T 504479E 5006332N; verbatimCoordinateSystem: UTM WGS 84; decimalLatitude: 45.210461; decimalLongitude: 9.057038; georeferencedBy: Silvia Stefanelli; georeferenceProtocol: GPS; **Identification:** identifiedBy: Giuseppe Platia; dateIdentified: 2011
- **Type status:** Other material. **Occurrence:** recordedBy: Silvia Stefanelli; individualCount: 3; lifeStage: adult; **Taxon:** taxonID: urn:lsid:faunaeur.org:taxname:235810; scientificName: Cardiophorus anticus; order: Coleoptera; family: Elateridae; genus: Cardiophorus; scientificNameAuthorship: Erichson 1840; **Location:** country: Italy; stateProvince: Pavia; locality: SIC "Boschi di Vaccarizza" - V1; verbatimElevation: 62 m; verbatimCoordinates: 32T 519272E 4999526N; verbatimCoordinateSystem: UTM WGS 84; decimalLatitude: 45.148947; decimalLongitude: 9.245157; georeferencedBy: Silvia Stefanelli; georeferenceProtocol: GPS; **Identification:** identifiedBy: Giuseppe Platia; dateIdentified: 2011

##### Distribution

Bulgaria, French mainland, Greek mainland, Italian mainland, Sardinia, Sicily, Slovakia, Near East (Fauna Europaea 2013).

#### Lacon punctatus

(Herbst, 1779)

##### Materials

- **Type status:** Other material. **Occurrence:** recordedBy: Silvia Stefanelli; individualCount: 2; lifeStage: adult; **Taxon:** taxonID: urn:lsid:faunaeur.org:taxname:236113; scientificName: Lacon punctatus; order: Coleoptera; family: Elateridae; genus: Lacon; scientificNameAuthorship: Herbst 1779; **Location:** country: Italy; stateProvince: Pavia; locality: SIC "Boschi Siro Negri e Moriano" - BN5; verbatimElevation: 62 m; verbatimCoordinates: 32T 502886E 5008393N; verbatimCoordinateSystem: UTM WGS 84; decimalLatitude: 45.229029; decimalLongitude: 9.036770; georeferencedBy: Silvia Stefanelli; georeferenceProtocol: GPS; **Identification:** identifiedBy: Giuseppe Platia; dateIdentified: 2011
- **Type status:** Other material. **Occurrence:** recordedBy: Silvia Stefanelli; individualCount: 2; lifeStage: adult; **Taxon:** taxonID: urn:lsid:faunaeur.org:taxname:236113; scientificName: Lacon punctatus; order: Coleoptera; family: Elateridae; genus: Lacon; scientificNameAuthorship: Herbst 1779; **Location:** country: Italy; stateProvince: Pavia; locality: SIC "Boschi Siro Negri e Moriano" - BN10; verbatimElevation: 76 m; verbatimCoordinates: 32T 504479E 5006332N; verbatimCoordinateSystem: UTM WGS 84; decimalLatitude: 45.210461; decimalLongitude: 9.057038; georeferencedBy: Silvia Stefanelli; georeferenceProtocol: GPS; **Identification:** identifiedBy: Giuseppe Platia; dateIdentified: 2011
- **Type status:** Other material. **Occurrence:** recordedBy: Silvia Stefanelli; individualCount: 4; lifeStage: adult; **Taxon:** taxonID: urn:lsid:faunaeur.org:taxname:236113; scientificName: Lacon punctatus; order: Coleoptera; family: Elateridae; genus: Lacon; scientificNameAuthorship: Herbst 1779; **Location:** country: Italy; stateProvince: Pavia; locality: SIC "Boschi di Vaccarizza" - V1; verbatimElevation: 62 m; verbatimCoordinates: 32T 519272E 4999526N; verbatimCoordinateSystem: UTM WGS 84; decimalLatitude: 45.148947; decimalLongitude: 9.245157; georeferencedBy: Silvia Stefanelli; georeferenceProtocol: GPS; **Identification:** identifiedBy: Giuseppe Platia; dateIdentified: 2011
- **Type status:** Other material. **Occurrence:** recordedBy: Silvia Stefanelli; individualCount: 3; lifeStage: adult; **Taxon:** taxonID: urn:lsid:faunaeur.org:taxname:236113; scientificName: Lacon punctatus; order: Coleoptera; family: Elateridae; genus: Lacon; scientificNameAuthorship: Herbst 1779; **Location:** country: Italy; stateProvince: Pavia; locality: SIC "Boschi di Vaccarizza" - V2; verbatimElevation: 65 m; verbatimCoordinates: 32T 519868E 4999488N; verbatimCoordinateSystem: UTM WGS 84; decimalLatitude: 45.148589; decimalLongitude: 9.252737; georeferencedBy: Silvia Stefanelli; georeferenceProtocol: GPS; **Identification:** identifiedBy: Giuseppe Platia; dateIdentified: 2011

##### Ecological interactions

###### Conservation status

Least Concern (European Environment Agency 2013).

##### Distribution

Balearic Is., Bosnia and Herzegovina, Bulgaria, Corsica, Crete, Croatia, Cyprus, Czech Republic, European Turkey, French mainland, Greek mainland, Hungary, Italian mainland, Malta, Portuguese mainland, Romania, Sardinia, Sicily, Slovakia, Spanish mainland, Sweden, Switzerland, Ukraine, Yugoslavia, East Palaearctic, Near East, North Africa (Fauna Europaea 2013).

##### Notes

The species lives in conifers, mainly pine, and in broadleaves like oak, beech, chestnut, willow, poplar, pear, and lime. The larva develops under the bark of standing and fallen dead trees and in stumps and trunks invaded by ants of the genus *Camponotus*. The adult has crepuscular and nocturnal habits and is often found together with the larvae (Platia 1994).

#### Melanotus (Melanotus) villosus

(Fourcroy, 1785)

Elater erythropus Gmelin, 1789 – Fauna Europaea (2013)
Elater rufipes Herbst, 1784 – Fauna Europaea (2013)

##### Materials

- **Type status:** Other material. **Occurrence:** recordedBy: Silvia Stefanelli; individualCount: 13; lifeStage: adult; **Taxon:** taxonID: urn:lsid:faunaeur.org:taxname:236167; scientificName: Melanotus villosus; order: Coleoptera; family: Elateridae; genus: Melanotus; **Location:** country: Italy; stateProvince: Pavia; locality: SIC "Boschi Siro Negri e Moriano" - BN10; verbatimElevation: 76 m; verbatimCoordinates: 32T 504479E 5006332N; verbatimCoordinateSystem: UTM WGS 84; decimalLatitude: 45.210461; decimalLongitude: 9.057038; georeferencedBy: Silvia Stefanelli; georeferenceProtocol: GPS; **Identification:** identifiedBy: Giuseppe Platia; dateIdentified: 2011

##### Ecological interactions

###### Conservation status

Least Concern (European Environment Agency 2013).

##### Distribution

Albania, Austria, Balearic Is., Belarus, Belgium, Bosnia and Herzegovina, Britain I., Bulgaria, Croatia, Czech Republic, Danish mainland, Estonia, European Turkey, French mainland, Germany, Greek mainland, Hungary, Ireland, Italian mainland, Kaliningrad Region, Latvia, Liechtenstein, Lithuania, Luxembourg, Macedonia, Moldova Republic of, Northern Ireland, Norwegian mainland, Poland, Portuguese mainland, Romania, Russia Central, Russia East, Russia North, Russia Northwest, Sicily, Slovakia, Slovenia, Spanish mainland, Sweden, Switzerland, The Netherlands, Ukraine, Yugoslavia, East Palaearctic, Near East, North Africa (Fauna Europaea 2013).

##### Notes

The species is common and widespread. The larva develops mainly in rotted timber, but also in decaying wood. The adult flies after dark and is attracted to light (Alexander 2002).

#### Dacne (Dacne) bipustulata

(Thunberg, 1781)

Cnecosophagus jekeli   Reitter, 1875 – Fauna Europaea (2013)
Dacne rugosa Jakowlev, 1910 – Fauna Europaea (2013)
Dermestes scanica Fabricius, 1775 – Fauna Europaea (2013)
Ips humeralis Fabricius, 1787 – Fauna Europaea (2013)
Dacne opaca Trella, 1929 – Fauna Europaea (2013)

##### Materials

- **Type status:** Other material. **Occurrence:** recordedBy: Silvia Stefanelli; individualCount: 9; lifeStage: adult; **Taxon:** taxonID: urn:lsid:faunaeur.org:taxname:188350; scientificName: Dacne bipustulata; order: Coleoptera; family: Erotylidae; genus: Dacne; scientificNameAuthorship: Thunberg 1781; **Location:** country: Italy; stateProvince: Pavia; locality: SIC "Boschi Siro Negri e Moriano" - BN1; verbatimElevation: 68 m; verbatimCoordinates: 32T 503258E 5007870N; verbatimCoordinateSystem: UTM WGS 84; decimalLatitude: 45.224312; decimalLongitude: 9.041499; georeferencedBy: Silvia Stefanelli; georeferenceProtocol: GPS; **Identification:** identifiedBy: Paolo Audisio; dateIdentified: 2011
- **Type status:** Other material. **Occurrence:** recordedBy: Silvia Stefanelli; individualCount: 23; lifeStage: adult; **Taxon:** taxonID: urn:lsid:faunaeur.org:taxname:188350; scientificName: Dacne bipustulata; order: Coleoptera; family: Erotylidae; genus: Dacne; scientificNameAuthorship: Thunberg 1781; **Location:** country: Italy; stateProvince: Pavia; locality: SIC "Boschi Siro Negri e Moriano" - BN21; verbatimElevation: 66 m; verbatimCoordinates: 32T 506342E 5005026N; verbatimCoordinateSystem: UTM WGS 84; decimalLatitude: 45.198691; decimalLongitude: 9.080746; georeferencedBy: Silvia Stefanelli; georeferenceProtocol: GPS; **Identification:** identifiedBy: Paolo Audisio; dateIdentified: 2011
- **Type status:** Other material. **Occurrence:** recordedBy: Silvia Stefanelli; individualCount: 39; lifeStage: adult; **Taxon:** taxonID: urn:lsid:faunaeur.org:taxname:188350; scientificName: Dacne bipustulata; order: Coleoptera; family: Erotylidae; genus: Dacne; scientificNameAuthorship: Thunberg 1781; **Location:** country: Italy; stateProvince: Pavia; locality: SIC "Boschi Siro Negri e Moriano" - BN5; verbatimElevation: 62 m; verbatimCoordinates: 32T 502886E 5008393N; verbatimCoordinateSystem: UTM WGS 84; decimalLatitude: 45.229029; decimalLongitude: 9.036770; georeferencedBy: Silvia Stefanelli; georeferenceProtocol: GPS; **Identification:** identifiedBy: Paolo Audisio; dateIdentified: 2011
- **Type status:** Other material. **Occurrence:** recordedBy: Silvia Stefanelli; individualCount: 4; lifeStage: adult; **Taxon:** taxonID: urn:lsid:faunaeur.org:taxname:188350; scientificName: Dacne bipustulata; order: Coleoptera; family: Erotylidae; genus: Dacne; scientificNameAuthorship: Thunberg 1781; **Location:** country: Italy; stateProvince: Pavia; locality: SIC "Boschi Siro Negri e Moriano" - BN10; verbatimElevation: 76 m; verbatimCoordinates: 32T 504479E 5006332N; verbatimCoordinateSystem: UTM WGS 84; decimalLatitude: 45.210461; decimalLongitude: 9.057038; georeferencedBy: Silvia Stefanelli; georeferenceProtocol: GPS; **Identification:** identifiedBy: Paolo Audisio; dateIdentified: 2011
- **Type status:** Other material. **Occurrence:** recordedBy: Silvia Stefanelli; individualCount: 1; lifeStage: adult; **Taxon:** taxonID: urn:lsid:faunaeur.org:taxname:188350; scientificName: Dacne bipustulata; order: Coleoptera; family: Erotylidae; genus: Dacne; scientificNameAuthorship: Thunberg 1781; **Location:** country: Italy; stateProvince: Pavia; locality: SIC "Boschi di Vaccarizza" - V1; verbatimElevation: 62 m; verbatimCoordinates: 32T 519272E 4999526N; verbatimCoordinateSystem: UTM WGS 84; decimalLatitude: 45.148947; decimalLongitude: 9.245157; georeferencedBy: Silvia Stefanelli; georeferenceProtocol: GPS; **Identification:** identifiedBy: Paolo Audisio; dateIdentified: 2011
- **Type status:** Other material. **Occurrence:** recordedBy: Silvia Stefanelli; individualCount: 1; lifeStage: adult; **Taxon:** taxonID: urn:lsid:faunaeur.org:taxname:188350; scientificName: Dacne bipustulata; order: Coleoptera; family: Erotylidae; genus: Dacne; scientificNameAuthorship: Thunberg 1781; **Location:** country: Italy; stateProvince: Pavia; locality: SIC "Boschi di Vaccarizza" - V2; verbatimElevation: 65 m; verbatimCoordinates: 32T 519868E 4999488N; verbatimCoordinateSystem: UTM WGS 84; decimalLatitude: 45.148589; decimalLongitude: 9.252737; georeferencedBy: Silvia Stefanelli; georeferenceProtocol: GPS; **Identification:** identifiedBy: Paolo Audisio; dateIdentified: 2011

##### Ecological interactions

###### Conservation status

Least Concern (European Environment Agency 2013).

##### Distribution

Albania, Austria, Belgium, Bosnia and Herzegovina, Britain I., Croatia, Czech Republic, Danish mainland, Estonia, Finland, French mainland, Germany, Greek mainland, Hungary, Ireland, Italian mainland, Latvia, Lithuania, Norwegian mainland, Poland, Romania, Russia Central, Russia North, Slovakia, Slovenia, Spanish mainland, Sweden, Switzerland, The Netherlands, Ukraine, Yugoslavia (Fauna Europaea 2013).

##### Notes

The species usually lives in softer polypore fungi, like *Letiporus sulphureus*, *Piptoporus betulinus*, and *Pleurotus ostreatus*, on trunks of broadleaves (Alexander and Anderson 2012).

#### Tritoma bipustulata

(Fabricius, 1775)

Cyrtotriplax binotata Reitter, 1887 – Fauna Europaea (2013)
Cyrtotriplax bipunctata Csiki, 1899 – Fauna Europaea (2013)
Tritoma dimidiata Redtenbacher, 1849 – Fauna Europaea (2013)
Dermestes humeralis Marsham, 1802 – Fauna Europaea (2013)
Tritoma incerta Rossi, 1790 – Fauna Europaea (2013)
Tritoma pretiosa Roubal, 1934 – Fauna Europaea (2013)
Cyrtotriplax pulchra Reitter, 1887 – Fauna Europaea (2013)
Sphaeridium bimaculata Herbst, 1783 – Fauna Europaea (2013)
Cyrtotriplax ehmanni   Dietl, 1898 – Fauna Europaea (2013)

##### Materials

- **Type status:** Other material. **Occurrence:** recordedBy: Silvia Stefanelli; individualCount: 1; lifeStage: adult; **Taxon:** taxonID: urn:lsid:faunaeur.org:taxname:188280; scientificName: Tritoma bipustulata; order: Coleoptera; family: Erotylidae; genus: Tritoma; scientificNameAuthorship: Fabricius 1775; **Location:** country: Italy; stateProvince: Pavia; locality: SIC "Boschi Siro Negri e Moriano" - BN1; verbatimElevation: 68 m; verbatimCoordinates: 32T 503258E 5007870N; verbatimCoordinateSystem: UTM WGS 84; decimalLatitude: 45.224312; decimalLongitude: 9.041499; georeferencedBy: Silvia Stefanelli; georeferenceProtocol: GPS; **Identification:** identifiedBy: Paolo Audisio; dateIdentified: 2011
- **Type status:** Other material. **Occurrence:** recordedBy: Silvia Stefanelli; individualCount: 6; lifeStage: adult; **Taxon:** taxonID: urn:lsid:faunaeur.org:taxname:188280; scientificName: Tritoma bipustulata; order: Coleoptera; family: Erotylidae; genus: Tritoma; scientificNameAuthorship: Fabricius 1775; **Location:** country: Italy; stateProvince: Pavia; locality: SIC "Boschi Siro Negri e Moriano" - BN5; verbatimElevation: 62 m; verbatimCoordinates: 32T 502886E 5008393N; verbatimCoordinateSystem: UTM WGS 84; decimalLatitude: 45.229029; decimalLongitude: 9.036770; georeferencedBy: Silvia Stefanelli; georeferenceProtocol: GPS; **Identification:** identifiedBy: Paolo Audisio; dateIdentified: 2011

##### Ecological interactions

###### Conservation status

Least Concern (European Environment Agency 2013).

##### Distribution

Austria, Belgium, Bosnia and Herzegovina, Britain I., Croatia, Czech Republic, Danish mainland, Estonia, Finland, French mainland, Germany, Greek mainland, Hungary, Italian mainland, Latvia, Liechtenstein, Norwegian mainland, Poland, Russia Central, Russia North, Slovakia, Slovenia, Spanish mainland, Sweden, Switzerland, The Netherlands, Ukraine (Fauna Europaea 2013).

##### Notes

The species occurs relatively often and throughout the year on tree fungi, especially on beeches where the larva also develops (Alexander 2002, Hůrka 2005).

#### Melasis buprestoides

(Linneaus, 1761)

##### Materials

- **Type status:** Other material. **Occurrence:** recordedBy: Silvia Stefanelli; individualCount: 1; lifeStage: adult; **Taxon:** taxonID: urn:lsid:faunaeur.org:taxname:188435; scientificName: Melasis buprestoides; order: Coleoptera; family: Eucnemidae; genus: Melasis; scientificNameAuthorship: Linnaeus 1761; **Location:** country: Italy; stateProvince: Pavia; locality: SIC "Boschi Siro Negri e Moriano" - BN1; verbatimElevation: 68 m; verbatimCoordinates: 32T 503258E 5007870N; verbatimCoordinateSystem: UTM WGS 84; decimalLatitude: 45.224312; decimalLongitude: 9.041499; georeferencedBy: Silvia Stefanelli; georeferenceProtocol: GPS; **Identification:** identifiedBy: Giuseppe Platia; dateIdentified: 2011
- **Type status:** Other material. **Occurrence:** recordedBy: Silvia Stefanelli; individualCount: 2; lifeStage: adult; **Taxon:** taxonID: urn:lsid:faunaeur.org:taxname:188435; scientificName: Melasis buprestoides; order: Coleoptera; family: Eucnemidae; genus: Melasis; scientificNameAuthorship: Linnaeus 1761; **Location:** country: Italy; stateProvince: Pavia; locality: SIC "Boschi Siro Negri e Moriano" - BN5; verbatimElevation: 62 m; verbatimCoordinates: 32T 502886E 5008393N; verbatimCoordinateSystem: UTM WGS 84; decimalLatitude: 45.229029; decimalLongitude: 9.036770; georeferencedBy: Silvia Stefanelli; georeferenceProtocol: GPS; **Identification:** identifiedBy: Giuseppe Platia; dateIdentified: 2011
- **Type status:** Other material. **Occurrence:** recordedBy: Silvia Stefanelli; individualCount: 4; lifeStage: adult; **Taxon:** taxonID: urn:lsid:faunaeur.org:taxname:188435; scientificName: Melasis buprestoides; order: Coleoptera; family: Eucnemidae; genus: Melasis; scientificNameAuthorship: Linnaeus 1761; **Location:** country: Italy; stateProvince: Pavia; locality: SIC "Boschi Siro Negri e Moriano" - BN10; verbatimElevation: 76 m; verbatimCoordinates: 32T 504479E 5006332N; verbatimCoordinateSystem: UTM WGS 84; decimalLatitude: 45.210461; decimalLongitude: 9.057038; georeferencedBy: Silvia Stefanelli; georeferenceProtocol: GPS; **Identification:** identifiedBy: Giuseppe Platia; dateIdentified: 2011
- **Type status:** Other material. **Occurrence:** recordedBy: Silvia Stefanelli; individualCount: 1; lifeStage: adult; **Taxon:** taxonID: urn:lsid:faunaeur.org:taxname:188435; scientificName: Melasis buprestoides; order: Coleoptera; family: Eucnemidae; genus: Melasis; scientificNameAuthorship: Linnaeus 1761; **Location:** country: Italy; stateProvince: Pavia; locality: SIC "Boschi di Vaccarizza" - V1; verbatimElevation: 62 m; verbatimCoordinates: 32T 519272E 4999526N; verbatimCoordinateSystem: UTM WGS 84; decimalLatitude: 45.148947; decimalLongitude: 9.245157; georeferencedBy: Silvia Stefanelli; georeferenceProtocol: GPS; **Identification:** identifiedBy: Giuseppe Platia; dateIdentified: 2011
- **Type status:** Other material. **Occurrence:** recordedBy: Silvia Stefanelli; individualCount: 2; lifeStage: adult; **Taxon:** taxonID: urn:lsid:faunaeur.org:taxname:188435; scientificName: Melasis buprestoides; order: Coleoptera; family: Eucnemidae; genus: Melasis; scientificNameAuthorship: Linnaeus 1761; **Location:** country: Italy; stateProvince: Pavia; locality: SIC "Boschi di Vaccarizza" - V2; verbatimElevation: 65 m; verbatimCoordinates: 32T 519868E 4999488N; verbatimCoordinateSystem: UTM WGS 84; decimalLatitude: 45.148589; decimalLongitude: 9.252737; georeferencedBy: Silvia Stefanelli; georeferenceProtocol: GPS; **Identification:** identifiedBy: Giuseppe Platia; dateIdentified: 2011

##### Ecological interactions

###### Conservation status

Least Concern (European Environment Agency 2013).

##### Distribution

Austria, Belarus, Belgium, Bosnia and Herzegovina, Britain I., Bulgaria, Corsica, Croatia, Czech Republic, Danish mainland, Finland, French mainland, Germany, Greek mainland, Hungary, Italian mainland, Kaliningrad Region, Macedonia, Norwegian mainland, Poland, Portuguese mainland, Romania, Russia Central, Russia South, Sardinia, Sicily, Slovakia, Slovenia, Spanish mainland, Sweden, Switzerland, Ukraine, Yugoslavia, Afro-tropical region, East Palaearctic, Near East (Fauna Europaea 2013).

##### Notes

The species is mostly found on beech, hornbeam, and elm trees. The larva develops in rather hard dead timber, especially boughs, of a wide variety of broadleaves (Alexander 2002, Hůrka 2005).

#### Nematodes filum

(Fabricius, 1801)

Elater filum Fabricius, 1801 – Fauna Europaea (2013)

##### Materials

- **Type status:** Other material. **Occurrence:** recordedBy: Silvia Stefanelli; individualCount: 1; lifeStage: adult; **Taxon:** taxonID: urn:lsid:faunaeur.org:taxname:188371; scientificName: Nematodes filum; order: Coleoptera; family: Eucnemidae; genus: Nematodes; scientificNameAuthorship: Fabricius 1801; **Location:** country: Italy; stateProvince: Pavia; locality: SIC "Boschi di Vaccarizza" - V1; verbatimElevation: 62 m; verbatimCoordinates: 32T 519272E 4999526N; verbatimCoordinateSystem: UTM WGS 84; decimalLatitude: 45.148947; decimalLongitude: 9.245157; georeferencedBy: Silvia Stefanelli; georeferenceProtocol: GPS; **Identification:** identifiedBy: Giuseppe Platia; dateIdentified: 2011

##### Distribution

Austria, French mainland, Germany, Hungary, Romania, Selvagens Is., Sicily, Slovakia, Switzerland, Near East (Fauna Europaea 2013).

##### Notes

For this species, only a single relict occurrence is known in Europe (Bussler et al. 2005).

#### Aeletes (Aeletes) atomarius

(Aube, 1843)

Aeletes (Aeletes) atomarius Synonyms: *Abraeus atomarius* Aube, 1843 – Fauna Europaea (2013)

##### Materials

- **Type status:** Other material. **Occurrence:** recordedBy: Silvia Stefanelli; individualCount: 2; lifeStage: adult; **Taxon:** taxonID: urn:lsid:faunaeur.org:taxname:119916; scientificName: Aeletes atomarius; order: Coleoptera; family: Histeridae; genus: Aeletes; scientificNameAuthorship: Aube 1843; **Location:** country: Italy; stateProvince: Pavia; locality: SIC "Boschi Siro Negri e Moriano" - BN1; verbatimElevation: 68 m; verbatimCoordinates: 32T 503258E 5007870N; verbatimCoordinateSystem: UTM WGS 84; decimalLatitude: 45.224312; decimalLongitude: 9.041499; georeferencedBy: Silvia Stefanelli; georeferenceProtocol: GPS; **Identification:** identifiedBy: Fabio Penati; dateIdentified: 2011
- **Type status:** Other material. **Occurrence:** recordedBy: Silvia Stefanelli; individualCount: 10; lifeStage: adult; **Taxon:** taxonID: urn:lsid:faunaeur.org:taxname:119916; scientificName: Aeletes atomarius; order: Coleoptera; family: Histeridae; genus: Aeletes; scientificNameAuthorship: Aube 1843; **Location:** country: Italy; stateProvince: Pavia; locality: SIC "Boschi Siro Negri e Moriano" - BN21; verbatimElevation: 66 m; verbatimCoordinates: 32T 506342E 5005026N; verbatimCoordinateSystem: UTM WGS 84; decimalLatitude: 45.198691; decimalLongitude: 9.080746; georeferencedBy: Silvia Stefanelli; georeferenceProtocol: GPS; **Identification:** identifiedBy: Fabio Penati; dateIdentified: 2011
- **Type status:** Other material. **Occurrence:** recordedBy: Silvia Stefanelli; individualCount: 2; lifeStage: adult; **Taxon:** taxonID: urn:lsid:faunaeur.org:taxname:119916; scientificName: Aeletes atomarius; order: Coleoptera; family: Histeridae; genus: Aeletes; scientificNameAuthorship: Aube 1843; **Location:** country: Italy; stateProvince: Pavia; locality: SIC "Boschi Siro Negri e Moriano" - BN5; verbatimElevation: 62 m; verbatimCoordinates: 32T 502886E 5008393N; verbatimCoordinateSystem: UTM WGS 84; decimalLatitude: 45.229029; decimalLongitude: 9.036770; georeferencedBy: Silvia Stefanelli; georeferenceProtocol: GPS; **Identification:** identifiedBy: Fabio Penati; dateIdentified: 2011
- **Type status:** Other material. **Occurrence:** recordedBy: Silvia Stefanelli; individualCount: 2; lifeStage: adult; **Taxon:** taxonID: urn:lsid:faunaeur.org:taxname:119916; scientificName: Aeletes atomarius; order: Coleoptera; family: Histeridae; genus: Aeletes; scientificNameAuthorship: Aube 1843; **Location:** country: Italy; stateProvince: Pavia; locality: SIC "Boschi Siro Negri e Moriano" - BN10; verbatimElevation: 76 m; verbatimCoordinates: 32T 504479E 5006332N; verbatimCoordinateSystem: UTM WGS 84; decimalLatitude: 45.210461; decimalLongitude: 9.057038; georeferencedBy: Silvia Stefanelli; georeferenceProtocol: GPS; **Identification:** identifiedBy: Fabio Penati; dateIdentified: 2011
- **Type status:** Other material. **Occurrence:** recordedBy: Silvia Stefanelli; individualCount: 1; lifeStage: adult; **Taxon:** taxonID: urn:lsid:faunaeur.org:taxname:119916; scientificName: Aeletes atomarius; order: Coleoptera; family: Histeridae; genus: Aeletes; scientificNameAuthorship: Aube 1843; **Location:** country: Italy; stateProvince: Pavia; locality: SIC "Boschi di Vaccarizza" - V1; verbatimElevation: 62 m; verbatimCoordinates: 32T 519272E 4999526N; verbatimCoordinateSystem: UTM WGS 84; decimalLatitude: 45.148947; decimalLongitude: 9.245157; georeferencedBy: Silvia Stefanelli; georeferenceProtocol: GPS; **Identification:** identifiedBy: Fabio Penati; dateIdentified: 2011

##### Distribution

Austria, Britain I., Bulgaria, Corsica, Czech Republic, Danish mainland, European Turkey, French mainland, Germany, Hungary, Italian mainland, Poland, Sardinia, Slovakia, Sweden, Ukraine, Yugoslavia (Fauna Europaea 2013).

##### Notes

The species lives in ancient wood pastures, in beech, ash, willow, and alder trees. It is usually found in the burrows of stag beetles *Dorcus parallelipipedus* in the moist crumbly decaying heartwood; although it has also been recorded with *Sinodendron cylindricum* and ant species such as *Lasius brunneus* (Alexander 2002).

#### Gnathoncus rotundatus

(Kugelann, 1792)

Saprinus deletus J.E. LeConte, 1844 – Fauna Europaea (2013)
Saprinus ignobilis Wollaston, 1864 – Fauna Europaea (2013)
Hister nanus Scriba, 1790 – Fauna Europaea (2013)
Hister piceus Marsham, 1802 – Fauna Europaea (2013)
Gnathoncus punctulatus Thomson, 1862 – Fauna Europaea (2013)
Hister quadristriatus Thunberg, 1794 – Fauna Europaea (2013)
Tribalus quadristriatus Wollaston, 1869 – Fauna Europaea (2013)
Saprinus wollastoni Marseul, 1864 – Fauna Europaea (2013)
Hister conjugatus Illiger, 1807 – Fauna Europaea (2013)
Hister punctatus Thunberg, 1794 – Fauna Europaea (2013)

##### Materials

- **Type status:** Other material. **Occurrence:** recordedBy: Silvia Stefanelli; individualCount: 1; lifeStage: adult; **Taxon:** taxonID: urn:lsid:faunaeur.org:taxname:119823; scientificName: Gnathoncus rotundatus; order: Coleoptera; family: Histeridae; genus: Gnathoncus; scientificNameAuthorship: Kugelann 1792; **Location:** country: Italy; stateProvince: Pavia; locality: SIC "Boschi Siro Negri e Moriano" - BN1; verbatimElevation: 68 m; verbatimCoordinates: 32T 503258E 5007870N; verbatimCoordinateSystem: UTM WGS 84; decimalLatitude: 45.224312; decimalLongitude: 9.041499; georeferencedBy: Silvia Stefanelli; georeferenceProtocol: GPS; **Identification:** identifiedBy: Fabio Penati; dateIdentified: 2011
- **Type status:** Other material. **Occurrence:** recordedBy: Silvia Stefanelli; individualCount: 1; lifeStage: adult; **Taxon:** taxonID: urn:lsid:faunaeur.org:taxname:119823; scientificName: Gnathoncus rotundatus; order: Coleoptera; family: Histeridae; genus: Gnathoncus; scientificNameAuthorship: Kugelann 1792; **Location:** country: Italy; stateProvince: Pavia; locality: SIC "Boschi Siro Negri e Moriano" - BN21; verbatimElevation: 66 m; verbatimCoordinates: 32T 506342E 5005026N; verbatimCoordinateSystem: UTM WGS 84; decimalLatitude: 45.198691; decimalLongitude: 9.080746; georeferencedBy: Silvia Stefanelli; georeferenceProtocol: GPS; **Identification:** identifiedBy: Fabio Penati; dateIdentified: 2011

##### Distribution

Albania, Austria, Belarus, Belgium, Bosnia and Herzegovina, Britain I., Bulgaria, Canary Is., Corsica, Crete, Croatia, Cyclades Is., Czech Republic, Danish mainland, Dodecanese Is., Estonia, European Turkey, Finland, French mainland, Germany, Greek mainland, Hungary, Italian mainland, Kaliningrad Region, Latvia, Lithuania, Luxembourg, Macedonia, Malta, Moldova Republic of, North Aegean Is., Norwegian mainland, Poland, Portuguese mainland, Romania, Russia Central, Russia East, Russia Northwest, Russia South, Sardinia, Sicily, Slovakia, Slovenia, Spanish mainland, Sweden, Switzerland, The Netherlands, Ukraine, Yugoslavia (Fauna Europaea 2013).

##### Notes

The species lives in all kinds of decaying organic matter, especially bat guano and bird nests. It is found in the nests of *Coloeus monedula spemologus*, *Sturnus vulgaris*, and *Strix aluco* (Vienna 1980).

#### Hololepta (Hololepta) plana

(Sulzer, 1776)

##### Materials

- **Type status:** Other material. **Occurrence:** recordedBy: Silvia Stefanelli; individualCount: 5; lifeStage: adult; **Taxon:** taxonID: urn:lsid:faunaeur.org:taxname:120110; scientificName: Hololepta plana; order: Coleoptera; family: Histeridae; genus: Hololepta; scientificNameAuthorship: Sulzer 1776; **Location:** country: Italy; stateProvince: Pavia; locality: SIC "Boschi Siro Negri e Moriano" - BN21; verbatimElevation: 66 m; verbatimCoordinates: 32T 506342E 5005026N; verbatimCoordinateSystem: UTM WGS 84; decimalLatitude: 45.198691; decimalLongitude: 9.080746; georeferencedBy: Silvia Stefanelli; georeferenceProtocol: GPS; **Identification:** identifiedBy: Fabio Penati; dateIdentified: 2011

##### Distribution

Albania, Andorra, Austria, Belarus, Belgium, Bosnia and Herzegovina, Bulgaria, Croatia, Czech Republic, Danish mainland, Estonia, European Turkey, Finland, French mainland, Germany, Greek mainland, Hungary, Italian mainland, Kaliningrad Region, Latvia, Liechtenstein, Lithuania, Luxembourg, Macedonia, Moldova Republic of, Norwegian mainland, Poland, Portuguese mainland, Romania, Russia Central, Russia Northwest, Russia South, Slovakia, Slovenia, Spanish mainland, Sweden, Switzerland, The Netherlands, Ukraine, Yugoslavia (Fauna Europaea 2013).

##### Notes

The species lives mainly in sub-hill habitats. It lives all stages in the ingrowings of *Populus alba*, *Populus pyramidalis* and *Pinus sylvestris* and often in trunks on the ground (Vienna 1980).

#### Paromalus (Paromalus) flavicornis

(Herbst, 1792)

Hister parvulus Rossi, 1792 – Fauna Europaea (2013)
Hister picipes Fabricius, 1798 – Fauna Europaea (2013)

##### Materials

- **Type status:** Other material. **Occurrence:** recordedBy: Silvia Stefanelli; individualCount: 9; lifeStage: adult; **Taxon:** taxonID: urn:lsid:faunaeur.org:taxname:120019; scientificName: Paromalus flavicornis; order: Coleoptera; family: Histeridae; genus: Paromalus; scientificNameAuthorship: Herbst 1792; **Location:** country: Italy; stateProvince: Pavia; locality: SIC "Boschi Siro Negri e Moriano" - BN1; verbatimElevation: 68 m; verbatimCoordinates: 32T 503258E 5007870N; verbatimCoordinateSystem: UTM WGS 84; decimalLatitude: 45.224312; decimalLongitude: 9.041499; georeferencedBy: Silvia Stefanelli; georeferenceProtocol: GPS; **Identification:** identifiedBy: Fabio Penati; dateIdentified: 2011
- **Type status:** Other material. **Occurrence:** recordedBy: Silvia Stefanelli; individualCount: 24; lifeStage: adult; **Taxon:** taxonID: urn:lsid:faunaeur.org:taxname:120019; scientificName: Paromalus flavicornis; order: Coleoptera; family: Histeridae; genus: Paromalus; scientificNameAuthorship: Herbst 1792; **Location:** country: Italy; stateProvince: Pavia; locality: SIC "Boschi Siro Negri e Moriano" - BN21; verbatimElevation: 66 m; verbatimCoordinates: 32T 506342E 5005026N; verbatimCoordinateSystem: UTM WGS 84; decimalLatitude: 45.198691; decimalLongitude: 9.080746; georeferencedBy: Silvia Stefanelli; georeferenceProtocol: GPS; **Identification:** identifiedBy: Fabio Penati; dateIdentified: 2011
- **Type status:** Other material. **Occurrence:** recordedBy: Silvia Stefanelli; individualCount: 23; lifeStage: adult; **Taxon:** taxonID: urn:lsid:faunaeur.org:taxname:120019; scientificName: Paromalus flavicornis; order: Coleoptera; family: Histeridae; genus: Paromalus; scientificNameAuthorship: Herbst 1792; **Location:** country: Italy; stateProvince: Pavia; locality: SIC "Boschi Siro Negri e Moriano" - BN5; verbatimElevation: 62 m; verbatimCoordinates: 32T 502886E 5008393N; verbatimCoordinateSystem: UTM WGS 84; decimalLatitude: 45.229029; decimalLongitude: 9.036770; georeferencedBy: Silvia Stefanelli; georeferenceProtocol: GPS; **Identification:** identifiedBy: Fabio Penati; dateIdentified: 2011
- **Type status:** Other material. **Occurrence:** recordedBy: Silvia Stefanelli; individualCount: 58; lifeStage: adult; **Taxon:** taxonID: urn:lsid:faunaeur.org:taxname:120019; scientificName: Paromalus flavicornis; order: Coleoptera; family: Histeridae; genus: Paromalus; scientificNameAuthorship: Herbst 1792; **Location:** country: Italy; stateProvince: Pavia; locality: SIC "Boschi Siro Negri e Moriano" - BN10; verbatimElevation: 76 m; verbatimCoordinates: 32T 504479E 5006332N; verbatimCoordinateSystem: UTM WGS 84; decimalLatitude: 45.210461; decimalLongitude: 9.057038; georeferencedBy: Silvia Stefanelli; georeferenceProtocol: GPS; **Identification:** identifiedBy: Fabio Penati; dateIdentified: 2011
- **Type status:** Other material. **Occurrence:** recordedBy: Silvia Stefanelli; individualCount: 58; lifeStage: adult; **Taxon:** taxonID: urn:lsid:faunaeur.org:taxname:120019; scientificName: Paromalus flavicornis; order: Coleoptera; family: Histeridae; genus: Paromalus; scientificNameAuthorship: Herbst 1792; **Location:** country: Italy; stateProvince: Pavia; locality: SIC "Boschi di Vaccarizza" - V1; verbatimElevation: 62 m; verbatimCoordinates: 32T 519272E 4999526N; verbatimCoordinateSystem: UTM WGS 84; decimalLatitude: 45.148947; decimalLongitude: 9.245157; georeferencedBy: Silvia Stefanelli; georeferenceProtocol: GPS; **Identification:** identifiedBy: Fabio Penati; dateIdentified: 2011
- **Type status:** Other material. **Occurrence:** recordedBy: Silvia Stefanelli; individualCount: 93; lifeStage: adult; **Taxon:** taxonID: urn:lsid:faunaeur.org:taxname:120019; scientificName: Paromalus flavicornis; order: Coleoptera; family: Histeridae; genus: Paromalus; scientificNameAuthorship: Herbst 1792; **Location:** country: Italy; stateProvince: Pavia; locality: SIC "Boschi di Vaccarizza" - V2; verbatimElevation: 65 m; verbatimCoordinates: 32T 519868E 4999488N; verbatimCoordinateSystem: UTM WGS 84; decimalLatitude: 45.148589; decimalLongitude: 9.252737; georeferencedBy: Silvia Stefanelli; georeferenceProtocol: GPS; **Identification:** identifiedBy: Fabio Penati; dateIdentified: 2011

##### Distribution

Albania, Andorra, Austria, Balearic Is., Belarus, Belgium, Bosnia and Herzegovina, Britain I., Bulgaria, Corsica, Crete, Croatia, Cyclades Is., Czech Republic, Danish mainland, Dodecanese Is., European Turkey, Finland, French mainland, Germany, Greek mainland, Hungary, Italian mainland, Liechtenstein, Macedonia, Moldova Republic of, North Aegean Is., Norwegian mainland, Poland, Portuguese mainland, Romania, Russia Northwest, Russia South, Sardinia, Sicily, Slovakia, Slovenia, Spanish mainland, Sweden, Switzerland, The Netherlands, Ukraine, Yugoslavia (Fauna Europaea 2013).

##### Notes

The species lives in old parks and ancient woodlands. It is found under the preferably dead bark of trees such as poplar, willow, oak, beech, walnut, chestnut, and maritime pine trees. It sometimes lives associated with ants such as *Lasius fuliginosus* and *Formica cunicularia*. The larva is a predator of other insect larvae and is often found in the burrows of *Bostrychus* sp. and *Crypturgus pusillus*. The adult appears in the spring and at the end of summer (Alexander 2002, Vienna 1980).

#### Platylomalus complanatus

(Panzer, 1796)

Hister nassatus Panzer, 1799 – Fauna Europaea (2013)
Platysoma theryana Reitter, 1890 – Fauna Europaea (2013)

##### Materials

- **Type status:** Other material. **Occurrence:** recordedBy: Silvia Stefanelli; individualCount: 7; lifeStage: adult; **Taxon:** taxonID: urn:lsid:faunaeur.org:taxname:120030; scientificName: Platylomalus complanatus; order: Coleoptera; family: Histeridae; genus: Platylomalus; scientificNameAuthorship: Panzer 1796; **Location:** country: Italy; stateProvince: Pavia; locality: SIC "Boschi di Vaccarizza" - V1; verbatimElevation: 62 m; verbatimCoordinates: 32T 519272E 4999526N; verbatimCoordinateSystem: UTM WGS 84; decimalLatitude: 45.148947; decimalLongitude: 9.245157; georeferencedBy: Silvia Stefanelli; georeferenceProtocol: GPS; **Identification:** identifiedBy: Fabio Penati; dateIdentified: 2011
- **Type status:** Other material. **Occurrence:** recordedBy: Silvia Stefanelli; individualCount: 4; lifeStage: adult; **Taxon:** taxonID: urn:lsid:faunaeur.org:taxname:120030; scientificName: Platylomalus complanatus; order: Coleoptera; family: Histeridae; genus: Platylomalus; scientificNameAuthorship: Panzer 1796; **Location:** country: Italy; stateProvince: Pavia; locality: SIC "Boschi di Vaccarizza" - V2; verbatimElevation: 65 m; verbatimCoordinates: 32T 519868E 4999488N; verbatimCoordinateSystem: UTM WGS 84; decimalLatitude: 45.148589; decimalLongitude: 9.252737; georeferencedBy: Silvia Stefanelli; georeferenceProtocol: GPS; **Identification:** identifiedBy: Fabio Penati; dateIdentified: 2011

##### Distribution

Austria, Belarus, Bosnia and Herzegovina, Bulgaria, Corsica, Croatia, Cyprus, Czech Republic, European Turkey, French mainland, Hungary, Italian mainland, Kaliningrad Region, Latvia, Macedonia, Norwegian mainland, Poland, Portuguese mainland, Romania, Russia South, Sicily, Slovakia, Slovenia, Spanish mainland, Sweden, Switzerland, Ukraine, Yugoslavia (Fauna Europaea 2013).

##### Notes

The species living range is from hills to mountains and in various types of decaying wood from both standing and fallen trees. The larva develops in poplars, beeches, oaks, and rarely in conifers (Vienna 1980).

#### Cryptolestes duplicatus

(Waltl, 1834)

##### Materials

- **Type status:** Other material. **Occurrence:** recordedBy: Silvia Stefanelli; individualCount: 1; lifeStage: adult; **Taxon:** taxonID: urn:lsid:faunaeur.org:taxname:189789; scientificName: Cryptolestes duplicatus; order: Coleoptera; family: Laemophloeidae; genus: Cryptolestes; scientificNameAuthorship: Waltl 1834; **Location:** country: Italy; stateProvince: Pavia; locality: SIC "Boschi Siro Negri e Moriano" - BN5; verbatimElevation: 62 m; verbatimCoordinates: 32T 502886E 5008393N; verbatimCoordinateSystem: UTM WGS 84; decimalLatitude: 45.229029; decimalLongitude: 9.036770; georeferencedBy: Silvia Stefanelli; georeferenceProtocol: GPS; **Identification:** identifiedBy: Gianfranco Salvato; dateIdentified: 2011
- **Type status:** Other material. **Occurrence:** recordedBy: Silvia Stefanelli; individualCount: 5; lifeStage: adult; **Taxon:** taxonID: urn:lsid:faunaeur.org:taxname:189789; scientificName: Cryptolestes duplicatus; order: Coleoptera; family: Laemophloeidae; genus: Cryptolestes; scientificNameAuthorship: Waltl 1834; **Location:** country: Italy; stateProvince: Pavia; locality: SIC "Boschi Siro Negri e Moriano" - BN10; verbatimElevation: 76 m; verbatimCoordinates: 32T 504479E 5006332N; verbatimCoordinateSystem: UTM WGS 84; decimalLatitude: 45.210461; decimalLongitude: 9.057038; georeferencedBy: Silvia Stefanelli; georeferenceProtocol: GPS; **Identification:** identifiedBy: Gianfranco Salvato; dateIdentified: 2011

##### Distribution

Austria, Czech Republic, French mainland, Germany, Hungary, Poland (Fauna Europaea 2013).

##### Notes

The species lives from the plains to the mountains, mainly in primary forests. The larva develops under the bark of Fagacaee, mainly oak and rarely beech trees (Ratti 1999).

#### Laemophloeus monilis

(Fabricius, 1787)

Cucujus monilis (Fabricius, 1787) – Fauna Europaea (2013)

##### Materials

- **Type status:** Other material. **Occurrence:** recordedBy: Silvia Stefanelli; individualCount: 1; lifeStage: adult; **Taxon:** taxonID: urn:lsid:faunaeur.org:taxname:189811; scientificName: Laemophloeus monilis; order: Coleoptera; family: Laemophloeidae; genus: Laemophloeus; scientificNameAuthorship: Fabricius 1787; **Location:** country: Italy; stateProvince: Pavia; locality: SIC "Boschi Siro Negri e Moriano" - BN1; verbatimElevation: 68 m; verbatimCoordinates: 32T 503258E 5007870N; verbatimCoordinateSystem: UTM WGS 84; decimalLatitude: 45.224312; decimalLongitude: 9.041499; georeferencedBy: Silvia Stefanelli; georeferenceProtocol: GPS; **Identification:** identifiedBy: Gianfranco Salvato; dateIdentified: 2011

##### Distribution

Austria, Belgium, Britain I., Corsica, Croatia, Czech Republic, French mainland, Germany, Hungary, Italian mainland, Poland, Russia South (Fauna Europaea 2013).

##### Notes

The species lives from the plains to the mountains, but also is found in urban parks. It is found under the bark of various species of broadleaves, mainly oaks, beeches, limes and maples, especially if fungi or Scolytidae are present (Fauna Europaea 2013).

#### Placonotus testaceus

(Fabricius, 1787)

##### Materials

- **Type status:** Other material. **Occurrence:** recordedBy: Silvia Stefanelli; individualCount: 21; lifeStage: adult; **Taxon:** scientificName: Placonotus testaceus; order: Coleoptera; family: Laemophloeidae; genus: Placonotus; scientificNameAuthorship: Fabricius 1787; **Location:** country: Italy; stateProvince: Pavia; locality: SIC "Boschi Siro Negri e Moriano" - BN1; verbatimElevation: 68 m; verbatimCoordinates: 32T 503258E 5007870N; verbatimCoordinateSystem: UTM WGS 84; decimalLatitude: 45.224312; decimalLongitude: 9.041499; georeferencedBy: Silvia Stefanelli; georeferenceProtocol: GPS; **Identification:** identifiedBy: Gianfranco Salvato; dateIdentified: 2011
- **Type status:** Other material. **Occurrence:** recordedBy: Silvia Stefanelli; individualCount: 16; lifeStage: adult; **Taxon:** scientificName: Placonotus testaceus; order: Coleoptera; family: Laemophloeidae; genus: Placonotus; scientificNameAuthorship: Fabricius 1787; **Location:** country: Italy; stateProvince: Pavia; locality: SIC "Boschi Siro Negri e Moriano" - BN21; verbatimElevation: 66 m; verbatimCoordinates: 32T 506342E 5005026N; verbatimCoordinateSystem: UTM WGS 84; decimalLatitude: 45.198691; decimalLongitude: 9.080746; georeferencedBy: Silvia Stefanelli; georeferenceProtocol: GPS; **Identification:** identifiedBy: Gianfranco Salvato; dateIdentified: 2011
- **Type status:** Other material. **Occurrence:** recordedBy: Silvia Stefanelli; individualCount: 205; lifeStage: adult; **Taxon:** scientificName: Placonotus testaceus; order: Coleoptera; family: Laemophloeidae; genus: Placonotus; scientificNameAuthorship: Fabricius 1787; **Location:** country: Italy; stateProvince: Pavia; locality: SIC "Boschi Siro Negri e Moriano" - BN5; verbatimElevation: 62 m; verbatimCoordinates: 32T 502886E 5008393N; verbatimCoordinateSystem: UTM WGS 84; decimalLatitude: 45.229029; decimalLongitude: 9.036770; georeferencedBy: Silvia Stefanelli; georeferenceProtocol: GPS; **Identification:** identifiedBy: Gianfranco Salvato; dateIdentified: 2011
- **Type status:** Other material. **Occurrence:** recordedBy: Silvia Stefanelli; individualCount: 10; lifeStage: adult; **Taxon:** scientificName: Placonotus testaceus; order: Coleoptera; family: Laemophloeidae; genus: Placonotus; scientificNameAuthorship: Fabricius 1787; **Location:** country: Italy; stateProvince: Pavia; locality: SIC "Boschi Siro Negri e Moriano" - BN10; verbatimElevation: 76 m; verbatimCoordinates: 32T 504479E 5006332N; verbatimCoordinateSystem: UTM WGS 84; decimalLatitude: 45.210461; decimalLongitude: 9.057038; georeferencedBy: Silvia Stefanelli; georeferenceProtocol: GPS; **Identification:** identifiedBy: Gianfranco Salvato; dateIdentified: 2011
- **Type status:** Other material. **Occurrence:** recordedBy: Silvia Stefanelli; individualCount: 20; lifeStage: adult; **Taxon:** scientificName: Placonotus testaceus; order: Coleoptera; family: Laemophloeidae; genus: Placonotus; scientificNameAuthorship: Fabricius 1787; **Location:** country: Italy; stateProvince: Pavia; locality: SIC "Boschi di Vaccarizza" - V1; verbatimElevation: 62 m; verbatimCoordinates: 32T 519272E 4999526N; verbatimCoordinateSystem: UTM WGS 84; decimalLatitude: 45.148947; decimalLongitude: 9.245157; georeferencedBy: Silvia Stefanelli; georeferenceProtocol: GPS; **Identification:** identifiedBy: Gianfranco Salvato; dateIdentified: 2011
- **Type status:** Other material. **Occurrence:** recordedBy: Silvia Stefanelli; individualCount: 21; lifeStage: adult; **Taxon:** scientificName: Placonotus testaceus; order: Coleoptera; family: Laemophloeidae; genus: Placonotus; scientificNameAuthorship: Fabricius 1787; **Location:** country: Italy; stateProvince: Pavia; locality: SIC "Boschi di Vaccarizza" - V2; verbatimElevation: 65 m; verbatimCoordinates: 32T 519868E 4999488N; verbatimCoordinateSystem: UTM WGS 84; decimalLatitude: 45.148589; decimalLongitude: 9.252737; georeferencedBy: Silvia Stefanelli; georeferenceProtocol: GPS; **Identification:** identifiedBy: Gianfranco Salvato; dateIdentified: 2011

##### Distribution

Austria, Belgium, Britain I., Croatia, Czech Republic, Danish mainland, European Turkey, Finland, French mainland, Germany, Greek mainland, Hungary, Italian mainland, Madeira, Poland, Portuguese mainland, Russia South, Sicily, Spanish mainland, Sweden, Ukraine, Yugoslavia (Fauna Europaea 2013).

##### Notes

The species lives from the plains to the hills and rarely in the mountains; they are mainly found in forests but are also found in urban habitats. It lives under the bark of various trees such as beech, oak, chestnut, elm, lime, maple, and pine trees. Sometimes it is found in galleries of Scolytidae (*Scolytus* sp., *Pteleobius* sp., *Taphrorychus* sp., *Tomicus* sp.). It is attracted to both fresh and fermented sap (Ratti 1999).

#### Enicmus rugosus

(Herbst, 1793)

Lathridius ferrugineus Gerhardt, 1912 – Fauna Europaea (2013)
Enicmus frater Weise, 1972 – Fauna Europaea (2013)
Lathridius ruficornis Kugelann, 1794 – Fauna Europaea (2013)
Lathridius rugipennis Mannerheim, 1844 – Fauna Europaea (2013)
Lathridius depressus Grimmer, 1841 – Fauna Europaea (2013)
Lathridius planatus Mannerheim, 1844 – Fauna Europaea (2013)

##### Materials

- **Type status:** Other material. **Occurrence:** recordedBy: Silvia Stefanelli; individualCount: 67; lifeStage: adult; **Taxon:** taxonID: urn:lsid:faunaeur.org:taxname:398120; scientificName: Enicmus rugosus; order: Coleoptera; family: Latridiidae; genus: Enicmus; scientificNameAuthorship: Herbst 1793; **Location:** country: Italy; stateProvince: Pavia; locality: SIC "Boschi Siro Negri e Moriano" - BN1; verbatimElevation: 68 m; verbatimCoordinates: 32T 503258E 5007870N; verbatimCoordinateSystem: UTM WGS 84; decimalLatitude: 45.224312; decimalLongitude: 9.041499; georeferencedBy: Silvia Stefanelli; georeferenceProtocol: GPS; **Identification:** identifiedBy: Wolfgang Rücker; dateIdentified: 2011
- **Type status:** Other material. **Occurrence:** recordedBy: Silvia Stefanelli; individualCount: 57; lifeStage: adult; **Taxon:** taxonID: urn:lsid:faunaeur.org:taxname:398120; scientificName: Enicmus rugosus; order: Coleoptera; family: Latridiidae; genus: Enicmus; scientificNameAuthorship: Herbst 1793; **Location:** country: Italy; stateProvince: Pavia; locality: SIC "Boschi Siro Negri e Moriano" - BN21; verbatimElevation: 66 m; verbatimCoordinates: 32T 506342E 5005026N; verbatimCoordinateSystem: UTM WGS 84; decimalLatitude: 45.198691; decimalLongitude: 9.080746; georeferencedBy: Silvia Stefanelli; georeferenceProtocol: GPS; **Identification:** identifiedBy: Wolfgang Rücker; dateIdentified: 2011
- **Type status:** Other material. **Occurrence:** recordedBy: Silvia Stefanelli; individualCount: 524; lifeStage: adult; **Taxon:** taxonID: urn:lsid:faunaeur.org:taxname:398120; scientificName: Enicmus rugosus; order: Coleoptera; family: Latridiidae; genus: Enicmus; scientificNameAuthorship: Herbst 1793; **Location:** country: Italy; stateProvince: Pavia; locality: SIC "Boschi Siro Negri e Moriano" - BN5; verbatimElevation: 62 m; verbatimCoordinates: 32T 502886E 5008393N; verbatimCoordinateSystem: UTM WGS 84; decimalLatitude: 45.229029; decimalLongitude: 9.036770; georeferencedBy: Silvia Stefanelli; georeferenceProtocol: GPS; **Identification:** identifiedBy: Wolfgang Rücker; dateIdentified: 2011
- **Type status:** Other material. **Occurrence:** recordedBy: Silvia Stefanelli; individualCount: 60; lifeStage: adult; **Taxon:** taxonID: urn:lsid:faunaeur.org:taxname:398120; scientificName: Enicmus rugosus; order: Coleoptera; family: Latridiidae; genus: Enicmus; scientificNameAuthorship: Herbst 1793; **Location:** country: Italy; stateProvince: Pavia; locality: SIC "Boschi Siro Negri e Moriano" - BN10; verbatimElevation: 76 m; verbatimCoordinates: 32T 504479E 5006332N; verbatimCoordinateSystem: UTM WGS 84; decimalLatitude: 45.210461; decimalLongitude: 9.057038; georeferencedBy: Silvia Stefanelli; georeferenceProtocol: GPS; **Identification:** identifiedBy: Wolfgang Rücker; dateIdentified: 2011
- **Type status:** Other material. **Occurrence:** recordedBy: Silvia Stefanelli; individualCount: 43; lifeStage: adult; **Taxon:** taxonID: urn:lsid:faunaeur.org:taxname:398120; scientificName: Enicmus rugosus; order: Coleoptera; family: Latridiidae; genus: Enicmus; scientificNameAuthorship: Herbst 1793; **Location:** country: Italy; stateProvince: Pavia; locality: SIC "Boschi di Vaccarizza" - V1; verbatimElevation: 62 m; verbatimCoordinates: 32T 519272E 4999526N; verbatimCoordinateSystem: UTM WGS 84; decimalLatitude: 45.148947; decimalLongitude: 9.245157; georeferencedBy: Silvia Stefanelli; georeferenceProtocol: GPS; **Identification:** identifiedBy: Wolfgang Rücker; dateIdentified: 2011
- **Type status:** Other material. **Occurrence:** recordedBy: Silvia Stefanelli; individualCount: 57; lifeStage: adult; **Taxon:** taxonID: urn:lsid:faunaeur.org:taxname:398120; scientificName: Enicmus rugosus; order: Coleoptera; family: Latridiidae; genus: Enicmus; scientificNameAuthorship: Herbst 1793; **Location:** country: Italy; stateProvince: Pavia; locality: SIC "Boschi di Vaccarizza" - V2; verbatimElevation: 65 m; verbatimCoordinates: 32T 519868E 4999488N; verbatimCoordinateSystem: UTM WGS 84; decimalLatitude: 45.148589; decimalLongitude: 9.252737; georeferencedBy: Silvia Stefanelli; georeferenceProtocol: GPS; **Identification:** identifiedBy: Wolfgang Rücker; dateIdentified: 2011

##### Distribution

Austria, Belarus, Belgium, Bosnia and Herzegovina, Britain I., Bulgaria, Croatia, Czech Republic, Danish mainland, Estonia, Finland, French mainland, Germany, Greek mainland, Hungary, Italian mainland, Latvia, Lithuania, Norwegian mainland, Poland, Portuguese mainland, Romania, Russia Central, Russia East, Russia North, Russia Northwest, Slovakia, Slovenia, Spanish mainland, Switzerland, The Netherlands, Ukraine, Yugoslavia, East Palaearctic, North Africa (Fauna Europaea 2013).

##### Notes

The species lives under the bark of old forest deadwood, mainly in oak but also in ash, beech, alder, and pine trees. It usually lives associated with fungi of the genus *Lycoperdacea*, *Polyporacea*, and myxomycetes (Alexander 2002, Rücker 2004).

#### Latridius hirtus

(Gyllenhal, 1827)

Enicmus distincticollis Roubal, 1933 – Fauna Europaea (2013)
Lathridius hirsutulus Stephens, 1829 – Fauna Europaea (2013)

##### Materials

- **Type status:** Other material. **Occurrence:** recordedBy: Silvia Stefanelli; individualCount: 3; lifeStage: adult; **Taxon:** taxonID: urn:lsid:faunaeur.org:taxname:398155; scientificName: Latridius hirtus; order: Coleoptera; family: Latridiidae; genus: Latridius; scientificNameAuthorship: Gyllenhal 1827; **Location:** country: Italy; stateProvince: Pavia; locality: SIC "Boschi Siro Negri e Moriano" - BN1; verbatimElevation: 68 m; verbatimCoordinates: 32T 503258E 5007870N; verbatimCoordinateSystem: UTM WGS 84; decimalLatitude: 45.224312; decimalLongitude: 9.041499; georeferencedBy: Silvia Stefanelli; georeferenceProtocol: GPS; **Identification:** identifiedBy: Wolfgang Rücker; dateIdentified: 2011
- **Type status:** Other material. **Occurrence:** recordedBy: Silvia Stefanelli; individualCount: 22; lifeStage: adult; **Taxon:** taxonID: urn:lsid:faunaeur.org:taxname:398155; scientificName: Latridius hirtus; order: Coleoptera; family: Latridiidae; genus: Latridius; scientificNameAuthorship: Gyllenhal 1827; **Location:** country: Italy; stateProvince: Pavia; locality: SIC "Boschi Siro Negri e Moriano" - BN21; verbatimElevation: 66 m; verbatimCoordinates: 32T 506342E 5005026N; verbatimCoordinateSystem: UTM WGS 84; decimalLatitude: 45.198691; decimalLongitude: 9.080746; georeferencedBy: Silvia Stefanelli; georeferenceProtocol: GPS; **Identification:** identifiedBy: Wolfgang Rücker; dateIdentified: 2011
- **Type status:** Other material. **Occurrence:** recordedBy: Silvia Stefanelli; individualCount: 2; lifeStage: adult; **Taxon:** taxonID: urn:lsid:faunaeur.org:taxname:398155; scientificName: Latridius hirtus; order: Coleoptera; family: Latridiidae; genus: Latridius; scientificNameAuthorship: Gyllenhal 1827; **Location:** country: Italy; stateProvince: Pavia; locality: SIC "Boschi Siro Negri e Moriano" - BN5; verbatimElevation: 62 m; verbatimCoordinates: 32T 502886E 5008393N; verbatimCoordinateSystem: UTM WGS 84; decimalLatitude: 45.229029; decimalLongitude: 9.036770; georeferencedBy: Silvia Stefanelli; georeferenceProtocol: GPS; **Identification:** identifiedBy: Wolfgang Rücker; dateIdentified: 2011
- **Type status:** Other material. **Occurrence:** recordedBy: Silvia Stefanelli; individualCount: 5; lifeStage: adult; **Taxon:** taxonID: urn:lsid:faunaeur.org:taxname:398155; scientificName: Latridius hirtus; order: Coleoptera; family: Latridiidae; genus: Latridius; scientificNameAuthorship: Gyllenhal 1827; **Location:** country: Italy; stateProvince: Pavia; locality: SIC "Boschi Siro Negri e Moriano" - BN10; verbatimElevation: 76 m; verbatimCoordinates: 32T 504479E 5006332N; verbatimCoordinateSystem: UTM WGS 84; decimalLatitude: 45.210461; decimalLongitude: 9.057038; georeferencedBy: Silvia Stefanelli; georeferenceProtocol: GPS; **Identification:** identifiedBy: Wolfgang Rücker; dateIdentified: 2011
- **Type status:** Other material. **Occurrence:** recordedBy: Silvia Stefanelli; individualCount: 8; lifeStage: adult; **Taxon:** taxonID: urn:lsid:faunaeur.org:taxname:398155; scientificName: Latridius hirtus; order: Coleoptera; family: Latridiidae; genus: Latridius; scientificNameAuthorship: Gyllenhal 1827; **Location:** country: Italy; stateProvince: Pavia; locality: SIC "Boschi di Vaccarizza" - V1; verbatimElevation: 62 m; verbatimCoordinates: 32T 519272E 4999526N; verbatimCoordinateSystem: UTM WGS 84; decimalLatitude: 45.148947; decimalLongitude: 9.245157; georeferencedBy: Silvia Stefanelli; georeferenceProtocol: GPS; **Identification:** identifiedBy: Wolfgang Rücker; dateIdentified: 2011
- **Type status:** Other material. **Occurrence:** recordedBy: Silvia Stefanelli; individualCount: 6; lifeStage: adult; **Taxon:** taxonID: urn:lsid:faunaeur.org:taxname:398155; scientificName: Latridius hirtus; order: Coleoptera; family: Latridiidae; genus: Latridius; scientificNameAuthorship: Gyllenhal 1827; **Location:** country: Italy; stateProvince: Pavia; locality: SIC "Boschi di Vaccarizza" - V2; verbatimElevation: 65 m; verbatimCoordinates: 32T 519868E 4999488N; verbatimCoordinateSystem: UTM WGS 84; decimalLatitude: 45.148589; decimalLongitude: 9.252737; georeferencedBy: Silvia Stefanelli; georeferenceProtocol: GPS; **Identification:** identifiedBy: Wolfgang Rücker; dateIdentified: 2011

##### Distribution

Austria, Belarus, Bosnia and Herzegovina, Croatia, Czech Republic, Danish mainland, Estonia, Finland, French mainland, Germany, Greek mainland, Hungary, Italian mainland, Latvia, Lithuania, Norwegian mainland, Poland, Romania, Russia Central, Russia North, Slovakia, Slovenia, Sweden, Switzerland, The Netherlands, Ukraine, East Palaearctic, Nearctic region (Fauna Europaea 2013).

##### Notes

The species lives in primary forests, and it is considered quite rare. It lives in both hardwood and softwood trunks if it is first attacked by fungi of the genus *Polyporacea* (Fauna Europaea 2013, Hůrka 2005).

#### Drapetes mordelloides

(Host, 1789)

##### Materials

- **Type status:** Other material. **Occurrence:** recordedBy: Silvia Stefanelli; individualCount: 1; lifeStage: adult; **Taxon:** scientificName: Drapetes mordelloides; order: Coleoptera; family: Lissomidae; genus: Drapetes; scientificNameAuthorship: Host 1789; **Location:** country: Italy; stateProvince: Pavia; locality: SIC "Boschi Siro Negri e Moriano" - BN1; verbatimElevation: 68 m; verbatimCoordinates: 32T 503258E 5007870N; verbatimCoordinateSystem: UTM WGS 84; decimalLatitude: 45.224312; decimalLongitude: 9.041499; georeferencedBy: Silvia Stefanelli; georeferenceProtocol: GPS; **Identification:** identifiedBy: Giuseppe Platia; dateIdentified: 2011
- **Type status:** Other material. **Occurrence:** recordedBy: Silvia Stefanelli; individualCount: 5; lifeStage: adult; **Taxon:** scientificName: Drapetes mordelloides; order: Coleoptera; family: Lissomidae; genus: Drapetes; scientificNameAuthorship: Host 1789; **Location:** country: Italy; stateProvince: Pavia; locality: SIC "Boschi Siro Negri e Moriano" - BN21; verbatimElevation: 66 m; verbatimCoordinates: 32T 506342E 5005026N; verbatimCoordinateSystem: UTM WGS 84; decimalLatitude: 45.198691; decimalLongitude: 9.080746; georeferencedBy: Silvia Stefanelli; georeferenceProtocol: GPS; **Identification:** identifiedBy: Giuseppe Platia; dateIdentified: 2011

#### Dorcus parallelipipedus

(Linneaus, 1785)

Dorcus truquiii Mulsant, 1855 – Fauna Europaea (2013)

##### Materials

- **Type status:** Other material. **Occurrence:** recordedBy: Silvia Stefanelli; individualCount: 49; lifeStage: adult; **Taxon:** taxonID: urn:lsid:faunaeur.org:taxname:123292; scientificName: Dorcus parallelipipedus; order: Coleoptera; family: Lucanidae; genus: Dorcus; scientificNameAuthorship: Linnaeus 1785; **Location:** country: Italy; stateProvince: Pavia; locality: SIC "Boschi Siro Negri e Moriano" - BN1; verbatimElevation: 68 m; verbatimCoordinates: 32T 503258E 5007870N; verbatimCoordinateSystem: UTM WGS 84; decimalLatitude: 45.224312; decimalLongitude: 9.041499; georeferencedBy: Silvia Stefanelli; georeferenceProtocol: GPS; **Identification:** identifiedBy: Giuseppe Carpaneto; dateIdentified: 2011
- **Type status:** Other material. **Occurrence:** recordedBy: Silvia Stefanelli; individualCount: 11; lifeStage: adult; **Taxon:** taxonID: urn:lsid:faunaeur.org:taxname:123292; scientificName: Dorcus parallelipipedus; order: Coleoptera; family: Lucanidae; genus: Dorcus; scientificNameAuthorship: Linnaeus 1785; **Location:** country: Italy; stateProvince: Pavia; locality: SIC "Boschi Siro Negri e Moriano" - BN21; verbatimElevation: 66 m; verbatimCoordinates: 32T 506342E 5005026N; verbatimCoordinateSystem: UTM WGS 84; decimalLatitude: 45.198691; decimalLongitude: 9.080746; georeferencedBy: Silvia Stefanelli; georeferenceProtocol: GPS; **Identification:** identifiedBy: Giuseppe Carpaneto; dateIdentified: 2011
- **Type status:** Other material. **Occurrence:** recordedBy: Silvia Stefanelli; individualCount: 7; lifeStage: adult; **Taxon:** taxonID: urn:lsid:faunaeur.org:taxname:123292; scientificName: Dorcus parallelipipedus; order: Coleoptera; family: Lucanidae; genus: Dorcus; scientificNameAuthorship: Linnaeus 1785; **Location:** country: Italy; stateProvince: Pavia; locality: SIC "Boschi Siro Negri e Moriano" - BN5; verbatimElevation: 62 m; verbatimCoordinates: 32T 502886E 5008393N; verbatimCoordinateSystem: UTM WGS 84; decimalLatitude: 45.229029; decimalLongitude: 9.036770; georeferencedBy: Silvia Stefanelli; georeferenceProtocol: GPS; **Identification:** identifiedBy: Giuseppe Carpaneto; dateIdentified: 2011
- **Type status:** Other material. **Occurrence:** recordedBy: Silvia Stefanelli; individualCount: 38; lifeStage: adult; **Taxon:** taxonID: urn:lsid:faunaeur.org:taxname:123292; scientificName: Dorcus parallelipipedus; order: Coleoptera; family: Lucanidae; genus: Dorcus; scientificNameAuthorship: Linnaeus 1785; **Location:** country: Italy; stateProvince: Pavia; locality: SIC "Boschi Siro Negri e Moriano" - BN10; verbatimElevation: 76 m; verbatimCoordinates: 32T 504479E 5006332N; verbatimCoordinateSystem: UTM WGS 84; decimalLatitude: 45.210461; decimalLongitude: 9.057038; georeferencedBy: Silvia Stefanelli; georeferenceProtocol: GPS; **Identification:** identifiedBy: Giuseppe Carpaneto; dateIdentified: 2011
- **Type status:** Other material. **Occurrence:** recordedBy: Silvia Stefanelli; individualCount: 77; lifeStage: adult; **Taxon:** taxonID: urn:lsid:faunaeur.org:taxname:123292; scientificName: Dorcus parallelipipedus; order: Coleoptera; family: Lucanidae; genus: Dorcus; scientificNameAuthorship: Linnaeus 1785; **Location:** country: Italy; stateProvince: Pavia; locality: SIC "Boschi di Vaccarizza" - V1; verbatimElevation: 62 m; verbatimCoordinates: 32T 519272E 4999526N; verbatimCoordinateSystem: UTM WGS 84; decimalLatitude: 45.148947; decimalLongitude: 9.245157; georeferencedBy: Silvia Stefanelli; georeferenceProtocol: GPS; **Identification:** identifiedBy: Giuseppe Carpaneto; dateIdentified: 2011
- **Type status:** Other material. **Occurrence:** recordedBy: Silvia Stefanelli; individualCount: 60; lifeStage: adult; **Taxon:** taxonID: urn:lsid:faunaeur.org:taxname:123292; scientificName: Dorcus parallelipipedus; order: Coleoptera; family: Lucanidae; genus: Dorcus; scientificNameAuthorship: Linnaeus 1785; **Location:** country: Italy; stateProvince: Pavia; locality: SIC "Boschi di Vaccarizza" - V2; verbatimElevation: 65 m; verbatimCoordinates: 32T 519868E 4999488N; verbatimCoordinateSystem: UTM WGS 84; decimalLatitude: 45.148589; decimalLongitude: 9.252737; georeferencedBy: Silvia Stefanelli; georeferenceProtocol: GPS; **Identification:** identifiedBy: Giuseppe Carpaneto; dateIdentified: 2011

##### Ecological interactions

###### Conservation status

Least Concern (European Environment Agency 2013).

##### Distribution

Austria, Belarus, Belgium, Britain I., Bulgaria, Corsica, Croatia, Czech Republic, Danish mainland, Estonia, European Turkey, French mainland, Germany, Greek mainland, Hungary, Ireland, Italian mainland, Latvia, Lithuania, Luxembourg, Norwegian mainland, Poland, Portuguese mainland, Russia Central, Russia East, Russia North, Russia Northwest, Russia South, Sardinia, Sicily, Slovakia, Slovenia, Spanish mainland, Sweden, Switzerland, The Netherlands, Ukraine, Near East, North Africa (Fauna Europaea 2013).

##### Notes

The species lives from the mountains to the Mediterranean lowlands. The larva develops in the rotten wood of various broadleaves and is often attacked by fungi. The adult flies on summer evenings, and it is attracted to light (Alexander 2002, Hůrka 2005)

#### Monotoma (Monotoma) longicollis

(Gyllenhal, 1827)

Monotoma gracilis Curtis, 1840 – Fauna Europaea (2013)
Monotoma angustata Stephens, 1830 – Fauna Europaea (2013)
Monotoma flavipes Kunze, 1839 – Fauna Europaea (2013)

##### Materials

- **Type status:** Other material. **Occurrence:** recordedBy: Silvia Stefanelli; individualCount: 1; lifeStage: adult; **Taxon:** taxonID: urn:lsid:faunaeur.org:taxname:190927; scientificName: Monotoma longicollis; order: Coleoptera; family: Monotomidae; genus: Monotoma; scientificNameAuthorship: Gyllenhal 1827; **Location:** country: Italy; stateProvince: Pavia; locality: SIC "Boschi Siro Negri e Moriano" - BN1; verbatimElevation: 68 m; verbatimCoordinates: 32T 503258E 5007870N; verbatimCoordinateSystem: UTM WGS 84; decimalLatitude: 45.224312; decimalLongitude: 9.041499; georeferencedBy: Silvia Stefanelli; georeferenceProtocol: GPS; **Identification:** identifiedBy: Gianfranco Salvato; dateIdentified: 2011
- **Type status:** Other material. **Occurrence:** recordedBy: Silvia Stefanelli; individualCount: 1; lifeStage: adult; **Taxon:** taxonID: urn:lsid:faunaeur.org:taxname:190927; scientificName: Monotoma longicollis; order: Coleoptera; family: Monotomidae; genus: Monotoma; scientificNameAuthorship: Gyllenhal 1827; **Location:** country: Italy; stateProvince: Pavia; locality: SIC "Boschi Siro Negri e Moriano" - BN21; verbatimElevation: 66 m; verbatimCoordinates: 32T 506342E 5005026N; verbatimCoordinateSystem: UTM WGS 84; decimalLatitude: 45.198691; decimalLongitude: 9.080746; georeferencedBy: Silvia Stefanelli; georeferenceProtocol: GPS; **Identification:** identifiedBy: Gianfranco Salvato; dateIdentified: 2011

##### Distribution

Austria, Britain I., Canary Is., Croatia, Czech Republic, Danish mainland, Finland, French mainland, Germany, Greek mainland, Hungary, Ireland, Italian mainland, Madeira, Northern Ireland, Norwegian mainland, Poland, Portuguese mainland, Romania, Russia Central, Russia North, Russia Northwest, Russia South, Sardinia, Slovakia, Spanish mainland, Sweden, Switzerland, Ukraine, Afro-tropical region, Australian region, East Palaearctic, Near East (Fauna Europaea 2013).

#### Rhizophagus (Rhizophagus) bipustulatus

(Fabricius, 1792)

Rhizophagus gyllenhalii C.G.Thomson, 1885 – Fauna Europaea (2013)
Rhizophagus magniceps Reitter, 1897 – Fauna Europaea (2013)
Rhizophagus bipunctatus Herbst, 1793 – Fauna Europaea (2013)
Rhizophagus longicollis Gyllenhal, 1827 – Fauna Europaea (2013)

##### Materials

- **Type status:** Other material. **Occurrence:** recordedBy: Silvia Stefanelli; individualCount: 33; lifeStage: adult; **Taxon:** taxonID: urn:lsid:faunaeur.org:taxname:190874; scientificName: Rhizophagus bipustulatus; order: Coleoptera; family: Monotomidae; genus: Rhizophagus; scientificNameAuthorship: Fabricius 1792; **Location:** country: Italy; stateProvince: Pavia; locality: SIC "Boschi Siro Negri e Moriano" - BN1; verbatimElevation: 68 m; verbatimCoordinates: 32T 503258E 5007870N; verbatimCoordinateSystem: UTM WGS 84; decimalLatitude: 45.224312; decimalLongitude: 9.041499; georeferencedBy: Silvia Stefanelli; georeferenceProtocol: GPS; **Identification:** identifiedBy: Gianfranco Salvato; dateIdentified: 2011
- **Type status:** Other material. **Occurrence:** recordedBy: Silvia Stefanelli; individualCount: 98; lifeStage: adult; **Taxon:** taxonID: urn:lsid:faunaeur.org:taxname:190874; scientificName: Rhizophagus bipustulatus; order: Coleoptera; family: Monotomidae; genus: Rhizophagus; scientificNameAuthorship: Fabricius 1792; **Location:** country: Italy; stateProvince: Pavia; locality: SIC "Boschi Siro Negri e Moriano" - BN21; verbatimElevation: 66 m; verbatimCoordinates: 32T 506342E 5005026N; verbatimCoordinateSystem: UTM WGS 84; decimalLatitude: 45.198691; decimalLongitude: 9.080746; georeferencedBy: Silvia Stefanelli; georeferenceProtocol: GPS; **Identification:** identifiedBy: Gianfranco Salvato; dateIdentified: 2011
- **Type status:** Other material. **Occurrence:** recordedBy: Silvia Stefanelli; individualCount: 50; lifeStage: adult; **Taxon:** taxonID: urn:lsid:faunaeur.org:taxname:190874; scientificName: Rhizophagus bipustulatus; order: Coleoptera; family: Monotomidae; genus: Rhizophagus; scientificNameAuthorship: Fabricius 1792; **Location:** country: Italy; stateProvince: Pavia; locality: SIC "Boschi Siro Negri e Moriano" - BN5; verbatimElevation: 62 m; verbatimCoordinates: 32T 502886E 5008393N; verbatimCoordinateSystem: UTM WGS 84; decimalLatitude: 45.229029; decimalLongitude: 9.036770; georeferencedBy: Silvia Stefanelli; georeferenceProtocol: GPS; **Identification:** identifiedBy: Gianfranco Salvato; dateIdentified: 2011
- **Type status:** Other material. **Occurrence:** recordedBy: Silvia Stefanelli; individualCount: 315; lifeStage: adult; **Taxon:** taxonID: urn:lsid:faunaeur.org:taxname:190874; scientificName: Rhizophagus bipustulatus; order: Coleoptera; family: Monotomidae; genus: Rhizophagus; scientificNameAuthorship: Fabricius 1792; **Location:** country: Italy; stateProvince: Pavia; locality: SIC "Boschi Siro Negri e Moriano" - BN10; verbatimElevation: 76 m; verbatimCoordinates: 32T 504479E 5006332N; verbatimCoordinateSystem: UTM WGS 84; decimalLatitude: 45.210461; decimalLongitude: 9.057038; georeferencedBy: Silvia Stefanelli; georeferenceProtocol: GPS; **Identification:** identifiedBy: Gianfranco Salvato; dateIdentified: 2011
- **Type status:** Other material. **Occurrence:** recordedBy: Silvia Stefanelli; individualCount: 53; lifeStage: adult; **Taxon:** taxonID: urn:lsid:faunaeur.org:taxname:190874; scientificName: Rhizophagus bipustulatus; order: Coleoptera; family: Monotomidae; genus: Rhizophagus; scientificNameAuthorship: Fabricius 1792; **Location:** country: Italy; stateProvince: Pavia; locality: SIC "Boschi di Vaccarizza" - V1; verbatimElevation: 62 m; verbatimCoordinates: 32T 519272E 4999526N; verbatimCoordinateSystem: UTM WGS 84; decimalLatitude: 45.148947; decimalLongitude: 9.245157; georeferencedBy: Silvia Stefanelli; georeferenceProtocol: GPS; **Identification:** identifiedBy: Gianfranco Salvato; dateIdentified: 2011
- **Type status:** Other material. **Occurrence:** recordedBy: Silvia Stefanelli; individualCount: 48; lifeStage: adult; **Taxon:** taxonID: urn:lsid:faunaeur.org:taxname:190874; scientificName: Rhizophagus bipustulatus; order: Coleoptera; family: Monotomidae; genus: Rhizophagus; scientificNameAuthorship: Fabricius 1792; **Location:** country: Italy; stateProvince: Pavia; locality: SIC "Boschi di Vaccarizza" - V2; verbatimElevation: 65 m; verbatimCoordinates: 32T 519868E 4999488N; verbatimCoordinateSystem: UTM WGS 84; decimalLatitude: 45.148589; decimalLongitude: 9.252737; georeferencedBy: Silvia Stefanelli; georeferenceProtocol: GPS; **Identification:** identifiedBy: Gianfranco Salvato; dateIdentified: 2011

##### Distribution

Austria, Belarus, Belgium, Bosnia and Herzegovina, Britain I., Bulgaria, Canary Is., Croatia, Czech Republic, Danish mainland, Estonia, European Turkey, Finland, French mainland, Germany, Greek mainland, Hungary, Italian mainland, Latvia, Liechtenstein, Lithuania, Luxembourg, Madeira, Norwegian mainland, Poland, Portuguese mainland, Romania, Russia Central, Russia East, Russia North, San Marino, Sardinia, Sicily, Slovakia, Slovenia, Spanish mainland, Sweden, Switzerland, The Netherlands, Ukraine, Yugoslavia, Near East, North Africa (Fauna Europaea 2013).

##### Notes

The species usually lives under bark on broadleaves trees, especially if attacked by fungi. The larva feeds on the mycelia and occasionally hunts bark beetles as most other species of the genus (Alexander 2002, Hůrka 2005).

#### Litargus (Litargus) connexus

(Geoffroy, 1785)

Engis lunatus Fabricius, 1792 – Fauna Europaea (2013)
Litargus mediojunctus Pic, 1903 – Fauna Europaea (2013)
Mycetophagus signatus Panzer, 1798 – Fauna Europaea (2013)
Ips bifasciatus Fabricius, 1787 – Fauna Europaea (2013)
Ips marginalis Panzer, 1793 – Fauna Europaea (2013)

##### Materials

- **Type status:** Other material. **Occurrence:** recordedBy: Silvia Stefanelli; individualCount: 101; lifeStage: adult; **Taxon:** taxonID: urn:lsid:faunaeur.org:taxname:124256; scientificName: Litargus connexus; order: Coleoptera; family: Mycetophagidae; genus: Litargus; scientificNameAuthorship: Geoffroy 1785; **Location:** country: Italy; stateProvince: Pavia; locality: SIC "Boschi Siro Negri e Moriano" - BN1; verbatimElevation: 68 m; verbatimCoordinates: 32T 503258E 5007870N; verbatimCoordinateSystem: UTM WGS 84; decimalLatitude: 45.224312; decimalLongitude: 9.041499; georeferencedBy: Silvia Stefanelli; georeferenceProtocol: GPS; **Identification:** identifiedBy: Claudio Canepari; dateIdentified: 2011
- **Type status:** Other material. **Occurrence:** recordedBy: Silvia Stefanelli; individualCount: 91; lifeStage: adult; **Taxon:** taxonID: urn:lsid:faunaeur.org:taxname:124256; scientificName: Litargus connexus; order: Coleoptera; family: Mycetophagidae; genus: Litargus; scientificNameAuthorship: Geoffroy 1785; **Location:** country: Italy; stateProvince: Pavia; locality: SIC "Boschi Siro Negri e Moriano" - BN21; verbatimElevation: 66 m; verbatimCoordinates: 32T 506342E 5005026N; verbatimCoordinateSystem: UTM WGS 84; decimalLatitude: 45.198691; decimalLongitude: 9.080746; georeferencedBy: Silvia Stefanelli; georeferenceProtocol: GPS; **Identification:** identifiedBy: Claudio Canepari; dateIdentified: 2011
- **Type status:** Other material. **Occurrence:** recordedBy: Silvia Stefanelli; individualCount: 115; lifeStage: adult; **Taxon:** taxonID: urn:lsid:faunaeur.org:taxname:124256; scientificName: Litargus connexus; order: Coleoptera; family: Mycetophagidae; genus: Litargus; scientificNameAuthorship: Geoffroy 1785; **Location:** country: Italy; stateProvince: Pavia; locality: SIC "Boschi Siro Negri e Moriano" - BN5; verbatimElevation: 62 m; verbatimCoordinates: 32T 502886E 5008393N; verbatimCoordinateSystem: UTM WGS 84; decimalLatitude: 45.229029; decimalLongitude: 9.036770; georeferencedBy: Silvia Stefanelli; georeferenceProtocol: GPS; **Identification:** identifiedBy: Claudio Canepari; dateIdentified: 2011
- **Type status:** Other material. **Occurrence:** recordedBy: Silvia Stefanelli; individualCount: 211; lifeStage: adult; **Taxon:** taxonID: urn:lsid:faunaeur.org:taxname:124256; scientificName: Litargus connexus; order: Coleoptera; family: Mycetophagidae; genus: Litargus; scientificNameAuthorship: Geoffroy 1785; **Location:** country: Italy; stateProvince: Pavia; locality: SIC "Boschi Siro Negri e Moriano" - BN10; verbatimElevation: 76 m; verbatimCoordinates: 32T 504479E 5006332N; verbatimCoordinateSystem: UTM WGS 84; decimalLatitude: 45.210461; decimalLongitude: 9.057038; georeferencedBy: Silvia Stefanelli; georeferenceProtocol: GPS; **Identification:** identifiedBy: Claudio Canepari; dateIdentified: 2011
- **Type status:** Other material. **Occurrence:** recordedBy: Silvia Stefanelli; individualCount: 125; lifeStage: adult; **Taxon:** taxonID: urn:lsid:faunaeur.org:taxname:124256; scientificName: Litargus connexus; order: Coleoptera; family: Mycetophagidae; genus: Litargus; scientificNameAuthorship: Geoffroy 1785; **Location:** country: Italy; stateProvince: Pavia; locality: SIC "Boschi di Vaccarizza" - V1; verbatimElevation: 62 m; verbatimCoordinates: 32T 519272E 4999526N; verbatimCoordinateSystem: UTM WGS 84; decimalLatitude: 45.148947; decimalLongitude: 9.245157; georeferencedBy: Silvia Stefanelli; georeferenceProtocol: GPS; **Identification:** identifiedBy: Claudio Canepari; dateIdentified: 2011
- **Type status:** Other material. **Occurrence:** recordedBy: Silvia Stefanelli; individualCount: 111; lifeStage: adult; **Taxon:** taxonID: urn:lsid:faunaeur.org:taxname:124256; scientificName: Litargus connexus; order: Coleoptera; family: Mycetophagidae; genus: Litargus; scientificNameAuthorship: Geoffroy 1785; **Location:** country: Italy; stateProvince: Pavia; locality: SIC "Boschi di Vaccarizza" - V2; verbatimElevation: 65 m; verbatimCoordinates: 32T 519868E 4999488N; verbatimCoordinateSystem: UTM WGS 84; decimalLatitude: 45.148589; decimalLongitude: 9.252737; georeferencedBy: Silvia Stefanelli; georeferenceProtocol: GPS; **Identification:** identifiedBy: Claudio Canepari; dateIdentified: 2011

##### Ecological interactions

###### Conservation status

Least Concern (European Environment Agency 2013).

##### Distribution

Albania, Austria, Belarus, Belgium, Bosnia and Herzegovina, Britain I., Bulgaria, Corsica, Croatia, Czech Republic, Danish mainland, Estonia, European Turkey, Finland, French mainland, Germany, Greek mainland, Hungary, Ireland, Italian mainland, Kaliningrad Region, Latvia, Liechtenstein, Lithuania, Macedonia, Moldova Republic of, Northern Ireland, Norwegian mainland, Poland, Portuguese mainland, Romania, Russia Central, Russia East, Russia North, Russia Northwest, Russia South, Sardinia, Sicily, Slovakia, Slovenia, Spanish mainland, Sweden, Switzerland, The Netherlands, Ukraine, Yugoslavia, East Palaearctic, Near East, North Africa (Fauna Europaea 2013).

##### Notes

The larvae feeds mainly on fungi belonging to the species *Daldinia loculata* and *Daldinia concentric* and more generally on Pyrenomycetes. The adult is found in rotten wood, especially if attacked by fungal decomposers (Alexander and Anderson 2012).

#### Mycetophagus (Ulolendus) piceus

(Fabricius, 1777)

Mycetophagus brunneus Panzer, 1798 – Fauna Europaea (2013)
Mycetophagus felicius Ragusa, 1892 – Fauna Europaea (2013)
Mycetophagus flavotinctus Roubal, 1931 – Fauna Europaea (2013)
Tritoma histrio C.Sahlberg, 1837 – Fauna Europaea (2013)
Tritoma humeralis Schilsky, 1888 – Fauna Europaea (2013)
Mycetophagus lunaris Fabricius, 1801 – Fauna Europaea (2013)
Tritoma punctulatus Schilsky, 1888 – Fauna Europaea (2013)
Boleteria undulatus (Marsham, 1802) – Fauna Europaea (2013)
Mycetophagus variabilis Hellwig, 1792 – Fauna Europaea (2013)
Boleteria varius Marsham, 1802 – Fauna Europaea (2013)
Mycetophagus bosnicus Apfelbeck, 1911 – Fauna Europaea (2013)
Mycetophagus hungaricus Papp, 1946 – Fauna Europaea (2013)

##### Materials

- **Type status:** Other material. **Occurrence:** recordedBy: Silvia Stefanelli; individualCount: 4; lifeStage: adult; **Taxon:** taxonID: urn:lsid:faunaeur.org:taxname:124237; scientificName: Mycetophagus piceus; order: Coleoptera; family: Mycetophagidae; genus: Mycetophagus; scientificNameAuthorship: Fabricius 1777; **Location:** country: Italy; stateProvince: Pavia; locality: SIC "Boschi Siro Negri e Moriano" - BN1; verbatimElevation: 68 m; verbatimCoordinates: 32T 503258E 5007870N; verbatimCoordinateSystem: UTM WGS 84; decimalLatitude: 45.224312; decimalLongitude: 9.041499; georeferencedBy: Silvia Stefanelli; georeferenceProtocol: GPS; **Identification:** identifiedBy: Gianfranco Salvato; dateIdentified: 2011
- **Type status:** Other material. **Occurrence:** recordedBy: Silvia Stefanelli; individualCount: 3; lifeStage: adult; **Taxon:** taxonID: urn:lsid:faunaeur.org:taxname:124237; scientificName: Mycetophagus piceus; order: Coleoptera; family: Mycetophagidae; genus: Mycetophagus; scientificNameAuthorship: Fabricius 1777; **Location:** country: Italy; stateProvince: Pavia; locality: SIC "Boschi Siro Negri e Moriano" - BN21; verbatimElevation: 66 m; verbatimCoordinates: 32T 506342E 5005026N; verbatimCoordinateSystem: UTM WGS 84; decimalLatitude: 45.198691; decimalLongitude: 9.080746; georeferencedBy: Silvia Stefanelli; georeferenceProtocol: GPS; **Identification:** identifiedBy: Gianfranco Salvato; dateIdentified: 2011
- **Type status:** Other material. **Occurrence:** recordedBy: Silvia Stefanelli; individualCount: 1; lifeStage: adult; **Taxon:** taxonID: urn:lsid:faunaeur.org:taxname:124237; scientificName: Mycetophagus piceus; order: Coleoptera; family: Mycetophagidae; genus: Mycetophagus; scientificNameAuthorship: Fabricius 1777; **Location:** country: Italy; stateProvince: Pavia; locality: SIC "Boschi Siro Negri e Moriano" - BN5; verbatimElevation: 62 m; verbatimCoordinates: 32T 502886E 5008393N; verbatimCoordinateSystem: UTM WGS 84; decimalLatitude: 45.229029; decimalLongitude: 9.036770; georeferencedBy: Silvia Stefanelli; georeferenceProtocol: GPS; **Identification:** identifiedBy: Gianfranco Salvato; dateIdentified: 2011

##### Ecological interactions

###### Conservation status

Least Concern (European Environment Agency 2013)

##### Distribution

Albania, Austria, Belarus, Belgium, Bosnia and Herzegovina, Britain I., Bulgaria, Croatia, Czech Republic, Danish mainland, Estonia, European Turkey, Finland, French mainland, Germany, Hungary, Italian mainland, Kaliningrad Region, Latvia, Lithuania, Norwegian mainland, Poland, Portuguese mainland, Romania, Russia Central, Russia East, Russia North, Russia Northwest, Russia South, Sicily, Slovakia, Slovenia, Spanish mainland, Sweden, Switzerland, The Netherlands, Ukraine, Yugoslavia, East Palaearctic, Near East (Fauna Europaea 2013).

##### Notes

The species lives primary in ancient forests and wood pastures. Both larva and adults are found in the fresh and moist trunks and branches of mainly oak trees, especially if attacked by the fungus *Laetiporus sulphureus* (Alexander 2002).

#### Mycetophagus (Mycetophagus) quadripustulatus

(Linnaeus, 1761)

Tritoma bipustulatus Schilsky, 1888 – Fauna Europaea (2013)
Silphoides boleti Herbst, 1783 – Fauna Europaea (2013)
Mycetophagus feliciae Ragusa, 1891 – Fauna Europaea (2013)
Silpha quadrimaculatus Schaller, 1783 – Fauna Europaea (2013)
Tritoma ruficollis  Schilsky, 1889 – Fauna Europaea (2013)
Mycetophagus winteri Reitter, 1911 – Fauna Europaea (2013)
Tritoma antemacularis Dalla Torre, 1879 – Fauna Europaea (2013)
Tritoma impustulatus Schilsky, 1888 – Fauna Europaea (2013)

##### Materials

- **Type status:** Other material. **Occurrence:** recordedBy: Silvia Stefanelli; individualCount: 22; lifeStage: adult; **Taxon:** taxonID: urn:lsid:faunaeur.org:taxname:124200; scientificName: Mycetophagus quadripustulatus; order: Coleoptera; family: Mycetophagidae; genus: Mycetophagus; scientificNameAuthorship: Linnaeus 1761; **Location:** country: Italy; stateProvince: Pavia; locality: SIC "Boschi Siro Negri e Moriano" - BN1; verbatimElevation: 68 m; verbatimCoordinates: 32T 503258E 5007870N; verbatimCoordinateSystem: UTM WGS 84; decimalLatitude: 45.224312; decimalLongitude: 9.041499; georeferencedBy: Silvia Stefanelli; georeferenceProtocol: GPS; **Identification:** identifiedBy: Claudio Canepari; dateIdentified: 2011
- **Type status:** Other material. **Occurrence:** recordedBy: Silvia Stefanelli; individualCount: 27; lifeStage: adult; **Taxon:** taxonID: urn:lsid:faunaeur.org:taxname:124200; scientificName: Mycetophagus quadripustulatus; order: Coleoptera; family: Mycetophagidae; genus: Mycetophagus; scientificNameAuthorship: Linnaeus 1761; **Location:** country: Italy; stateProvince: Pavia; locality: SIC "Boschi Siro Negri e Moriano" - BN21; verbatimElevation: 66 m; verbatimCoordinates: 32T 506342E 5005026N; verbatimCoordinateSystem: UTM WGS 84; decimalLatitude: 45.198691; decimalLongitude: 9.080746; georeferencedBy: Silvia Stefanelli; georeferenceProtocol: GPS; **Identification:** identifiedBy: Claudio Canepari; dateIdentified: 2011
- **Type status:** Other material. **Occurrence:** recordedBy: Silvia Stefanelli; individualCount: 6; lifeStage: adult; **Taxon:** taxonID: urn:lsid:faunaeur.org:taxname:124200; scientificName: Mycetophagus quadripustulatus; order: Coleoptera; family: Mycetophagidae; genus: Mycetophagus; scientificNameAuthorship: Linnaeus 1761; **Location:** country: Italy; stateProvince: Pavia; locality: SIC "Boschi Siro Negri e Moriano" - BN5; verbatimElevation: 62 m; verbatimCoordinates: 32T 502886E 5008393N; verbatimCoordinateSystem: UTM WGS 84; decimalLatitude: 45.229029; decimalLongitude: 9.036770; georeferencedBy: Silvia Stefanelli; georeferenceProtocol: GPS; **Identification:** identifiedBy: Claudio Canepari; dateIdentified: 2011
- **Type status:** Other material. **Occurrence:** recordedBy: Silvia Stefanelli; individualCount: 6; lifeStage: adult; **Taxon:** taxonID: urn:lsid:faunaeur.org:taxname:124200; scientificName: Mycetophagus quadripustulatus; order: Coleoptera; family: Mycetophagidae; genus: Mycetophagus; scientificNameAuthorship: Linnaeus 1761; **Location:** country: Italy; stateProvince: Pavia; locality: SIC "Boschi Siro Negri e Moriano" - BN10; verbatimElevation: 76 m; verbatimCoordinates: 32T 504479E 5006332N; verbatimCoordinateSystem: UTM WGS 84; decimalLatitude: 45.210461; decimalLongitude: 9.057038; georeferencedBy: Silvia Stefanelli; georeferenceProtocol: GPS; **Identification:** identifiedBy: Claudio Canepari; dateIdentified: 2011
- **Type status:** Other material. **Occurrence:** recordedBy: Silvia Stefanelli; individualCount: 2; lifeStage: adult; **Taxon:** taxonID: urn:lsid:faunaeur.org:taxname:124200; scientificName: Mycetophagus quadripustulatus; order: Coleoptera; family: Mycetophagidae; genus: Mycetophagus; scientificNameAuthorship: Linnaeus 1761; **Location:** country: Italy; stateProvince: Pavia; locality: SIC "Boschi di Vaccarizza" - V1; verbatimElevation: 62 m; verbatimCoordinates: 32T 519272E 4999526N; verbatimCoordinateSystem: UTM WGS 84; decimalLatitude: 45.148947; decimalLongitude: 9.245157; georeferencedBy: Silvia Stefanelli; georeferenceProtocol: GPS; **Identification:** identifiedBy: Claudio Canepari; dateIdentified: 2011

##### Ecological interactions

###### Conservation status

Least Concern (European Environment Agency 2013).

##### Distribution

Albania, Austria, Belarus, Belgium, Bosnia and Herzegovina, Britain I., Bulgaria, Channel Is., Corsica, Croatia, Czech Republic, Danish mainland, Estonia, European Turkey, Finland, French mainland, Germany, Greek mainland, Hungary, Italian mainland, Kaliningrad Region, Latvia, Lithuania, Macedonia, Moldova Republic of, Norwegian mainland, Poland, Portuguese mainland, Romania, Russia Central, Russia East, Russia North, Russia Northwest, Russia South, Sardinia, Sicily, Slovakia, Slovenia, Spanish mainland, Sweden, Switzerland, The Netherlands, Ukraine, Yugoslavia, East Palaearctic, Near East, North Africa (Fauna Europaea 2013).

##### Notes

This very rare species lives in old decaying broadleaves timbers with mildewed cavities. Sometimes it is found in stored products where fungal decay occurred (Alexander 2002).

#### Cryptarcha strigata

(Fabricius, 1787)

Nitidula strigata Fabricius, 1787 – Fauna Europaea (2013)

##### Materials

- **Type status:** Other material. **Occurrence:** recordedBy: Silvia Stefanelli; individualCount: 2; lifeStage: adult; **Taxon:** taxonID: urn:lsid:faunaeur.org:taxname:377854; scientificName: Cryptarcha strigata; order: Coleoptera; family: Nitidulidae; genus: Cryptarcha ; scientificNameAuthorship: Fabricius 1787; **Location:** country: Italy; stateProvince: Pavia; locality: SIC "Boschi Siro Negri e Moriano" - BN5; verbatimElevation: 62 m; verbatimCoordinates: 32T 502886E 5008393N; verbatimCoordinateSystem: UTM WGS 84; decimalLatitude: 45.229029; decimalLongitude: 9.036770; georeferencedBy: Silvia Stefanelli; georeferenceProtocol: GPS; **Identification:** identifiedBy: Paolo Audisio; dateIdentified: 2011

##### Distribution

Albania, Andorra, Austria, Belarus, Belgium, Bosnia and Herzegovina, Britain I., Bulgaria, Croatia, Czech Republic, Danish mainland, Estonia, European Turkey, Finland, French mainland, Germany, Greek mainland, Hungary, Ireland, Italian mainland, Kaliningrad Region, Latvia, Lithuania, Luxembourg, Macedonia, Moldova Republic of, Northern Ireland, Norwegian mainland, Poland, Portuguese mainland, Romania, Russia Central, Russia East, Russia North, Russia Northwest, Russia South, Sardinia, Sicily, Slovakia, Slovenia, Spanish mainland, Sweden, Switzerland, The Netherlands, Ukraine, Yugoslavia, East Palaearctic (Fauna Europaea 2013).

##### Notes

The species lives in mesophilic woodlands, especially lowland oak forests, but is also found in mixed forests. It lives associated with macromycetes in decaying trees (Audisio 1993).

#### Epuraea aestiva

(Linneaus, 1758)

Nitidula depressa Illiger, 1798 – Fauna Europaea (2013)
Epuraea ochracea Sturm, 1844 – Fauna Europaea (2013)
Epuraea bisignata Sturm, 1844 – Fauna Europaea (2013)
Epuraea grandiclava Roubal, 1939 – Fauna Europaea (2013)

##### Materials

- **Type status:** Other material. **Occurrence:** recordedBy: Silvia Stefanelli; individualCount: 1; lifeStage: adult; **Taxon:** taxonID: urn:lsid:faunaeur.org:taxname:377682; scientificName: Epuraea aestiva; order: Coleoptera; family: Nitidulidae; genus: Epuraea; scientificNameAuthorship: Linnaeus 1758; **Location:** country: Italy; stateProvince: Pavia; locality: SIC "Boschi Siro Negri e Moriano" - BN1; verbatimElevation: 68 m; verbatimCoordinates: 32T 503258E 5007870N; verbatimCoordinateSystem: UTM WGS 84; decimalLatitude: 45.224312; decimalLongitude: 9.041499; georeferencedBy: Silvia Stefanelli; georeferenceProtocol: GPS; **Identification:** identifiedBy: Paolo Audisio; dateIdentified: 2011
- **Type status:** Other material. **Occurrence:** recordedBy: Silvia Stefanelli; individualCount: 1; lifeStage: adult; **Taxon:** taxonID: urn:lsid:faunaeur.org:taxname:377682; scientificName: Epuraea aestiva; order: Coleoptera; family: Nitidulidae; genus: Epuraea; scientificNameAuthorship: Linnaeus 1758; **Location:** country: Italy; stateProvince: Pavia; locality: SIC "Boschi Siro Negri e Moriano" - BN5; verbatimElevation: 62 m; verbatimCoordinates: 32T 502886E 5008393N; verbatimCoordinateSystem: UTM WGS 84; decimalLatitude: 45.229029; decimalLongitude: 9.036770; georeferencedBy: Silvia Stefanelli; georeferenceProtocol: GPS; **Identification:** identifiedBy: Paolo Audisio; dateIdentified: 2011
- **Type status:** Other material. **Occurrence:** recordedBy: Silvia Stefanelli; individualCount: 1; lifeStage: adult; **Taxon:** taxonID: urn:lsid:faunaeur.org:taxname:377682; scientificName: Epuraea aestiva; order: Coleoptera; family: Nitidulidae; genus: Epuraea; scientificNameAuthorship: Linnaeus 1758; **Location:** country: Italy; stateProvince: Pavia; locality: SIC "Boschi Siro Negri e Moriano" - BN10; verbatimElevation: 76 m; verbatimCoordinates: 32T 504479E 5006332N; verbatimCoordinateSystem: UTM WGS 84; decimalLatitude: 45.210461; decimalLongitude: 9.057038; georeferencedBy: Silvia Stefanelli; georeferenceProtocol: GPS; **Identification:** identifiedBy: Paolo Audisio; dateIdentified: 2011
- **Type status:** Other material. **Occurrence:** recordedBy: Silvia Stefanelli; individualCount: 1; lifeStage: adult; **Taxon:** taxonID: urn:lsid:faunaeur.org:taxname:377682; scientificName: Epuraea aestiva; order: Coleoptera; family: Nitidulidae; genus: Epuraea; scientificNameAuthorship: Linnaeus 1758; **Location:** country: Italy; stateProvince: Pavia; locality: SIC "Boschi di Vaccarizza" - V1; verbatimElevation: 62 m; verbatimCoordinates: 32T 519272E 4999526N; verbatimCoordinateSystem: UTM WGS 84; decimalLatitude: 45.148947; decimalLongitude: 9.245157; georeferencedBy: Silvia Stefanelli; georeferenceProtocol: GPS; **Identification:** identifiedBy: Paolo Audisio; dateIdentified: 2011

##### Distribution

Albania, Andorra, Austria, Azores, Belarus, Belgium, Bosnia and Herzegovina, Britain I., Bulgaria, Corsica, Croatia, Czech Republic, Danish mainland, Dodecanese Is., Estonia, European Turkey, Finland, French mainland, Germany, Greek mainland, Hungary, Ireland, Italian mainland, Kaliningrad Region, Latvia, Liechtenstein, Lithuania, Luxembourg, Macedonia, Moldova Republic of, Monaco, North Aegean Is., Northern Ireland, Norwegian mainland, Poland, Portuguese mainland, Romania, Russia Central, Russia East, Russia North, Russia Northwest, Russia South, San Marino, Sardinia, Sicily, Slovakia, Slovenia, Spanish mainland, Sweden, Switzerland, The Netherlands, Ukraine, Yugoslavia, East Palaearctic, Near East, Nearctic region (Fauna Europaea 2013).

##### Notes

The larva and adults can be found in the galleries of ambrosia beetles, at oozing tree sap, and in various fungi. The larva develops in the nests of bumblebees and the adult occur on flowers and during winter can be found in mole nests (Hůrka 2005).

#### Epuraea guttata

(Oliver, 1811)

Nitidula decemguttata Fabricius, 1792 – Fauna Europaea (2013)

##### Materials

- **Type status:** Other material. **Occurrence:** recordedBy: Silvia Stefanelli; individualCount: 12; lifeStage: adult; **Taxon:** taxonID: urn:lsid:faunaeur.org:taxname:377721; scientificName: Epuraea guttata; order: Coleoptera; family: Nitidulidae; genus: Epuraea; scientificNameAuthorship: Olivier 1811; **Location:** country: Italy; stateProvince: Pavia; locality: SIC "Boschi Siro Negri e Moriano" - BN1; verbatimElevation: 68 m; verbatimCoordinates: 32T 503258E 5007870N; verbatimCoordinateSystem: UTM WGS 84; decimalLatitude: 45.224312; decimalLongitude: 9.041499; georeferencedBy: Silvia Stefanelli; georeferenceProtocol: GPS; **Identification:** identifiedBy: Paolo Audisio; dateIdentified: 2011
- **Type status:** Other material. **Occurrence:** recordedBy: Silvia Stefanelli; individualCount: 6; lifeStage: adult; **Taxon:** taxonID: urn:lsid:faunaeur.org:taxname:377721; scientificName: Epuraea guttata; order: Coleoptera; family: Nitidulidae; genus: Epuraea; scientificNameAuthorship: Olivier 1811; **Location:** country: Italy; stateProvince: Pavia; locality: SIC "Boschi Siro Negri e Moriano" - BN21; verbatimElevation: 66 m; verbatimCoordinates: 32T 506342E 5005026N; verbatimCoordinateSystem: UTM WGS 84; decimalLatitude: 45.198691; decimalLongitude: 9.080746; georeferencedBy: Silvia Stefanelli; georeferenceProtocol: GPS; **Identification:** identifiedBy: Paolo Audisio; dateIdentified: 2011
- **Type status:** Other material. **Occurrence:** recordedBy: Silvia Stefanelli; individualCount: 39; lifeStage: adult; **Taxon:** taxonID: urn:lsid:faunaeur.org:taxname:377721; scientificName: Epuraea guttata; order: Coleoptera; family: Nitidulidae; genus: Epuraea; scientificNameAuthorship: Olivier 1811; **Location:** country: Italy; stateProvince: Pavia; locality: SIC "Boschi Siro Negri e Moriano" - BN5; verbatimElevation: 62 m; verbatimCoordinates: 32T 502886E 5008393N; verbatimCoordinateSystem: UTM WGS 84; decimalLatitude: 45.229029; decimalLongitude: 9.036770; georeferencedBy: Silvia Stefanelli; georeferenceProtocol: GPS; **Identification:** identifiedBy: Paolo Audisio; dateIdentified: 2011
- **Type status:** Other material. **Occurrence:** recordedBy: Silvia Stefanelli; individualCount: 52; lifeStage: adult; **Taxon:** taxonID: urn:lsid:faunaeur.org:taxname:377721; scientificName: Epuraea guttata; order: Coleoptera; family: Nitidulidae; genus: Epuraea; scientificNameAuthorship: Olivier 1811; **Location:** country: Italy; stateProvince: Pavia; locality: SIC "Boschi Siro Negri e Moriano" - BN10; verbatimElevation: 76 m; verbatimCoordinates: 32T 504479E 5006332N; verbatimCoordinateSystem: UTM WGS 84; decimalLatitude: 45.210461; decimalLongitude: 9.057038; georeferencedBy: Silvia Stefanelli; georeferenceProtocol: GPS; **Identification:** identifiedBy: Paolo Audisio; dateIdentified: 2011
- **Type status:** Other material. **Occurrence:** recordedBy: Silvia Stefanelli; individualCount: 3; lifeStage: adult; **Taxon:** taxonID: urn:lsid:faunaeur.org:taxname:377721; scientificName: Epuraea guttata; order: Coleoptera; family: Nitidulidae; genus: Epuraea; scientificNameAuthorship: Olivier 1811; **Location:** country: Italy; stateProvince: Pavia; locality: SIC "Boschi di Vaccarizza" - V1; verbatimElevation: 62 m; verbatimCoordinates: 32T 519272E 4999526N; verbatimCoordinateSystem: UTM WGS 84; decimalLatitude: 45.148947; decimalLongitude: 9.245157; georeferencedBy: Silvia Stefanelli; georeferenceProtocol: GPS; **Identification:** identifiedBy: Paolo Audisio; dateIdentified: 2011
- **Type status:** Other material. **Occurrence:** recordedBy: Silvia Stefanelli; individualCount: 5; lifeStage: adult; **Taxon:** taxonID: urn:lsid:faunaeur.org:taxname:377721; scientificName: Epuraea guttata; order: Coleoptera; family: Nitidulidae; genus: Epuraea; scientificNameAuthorship: Olivier 1811; **Location:** country: Italy; stateProvince: Pavia; locality: SIC "Boschi di Vaccarizza" - V2; verbatimElevation: 65 m; verbatimCoordinates: 32T 519868E 4999488N; verbatimCoordinateSystem: UTM WGS 84; decimalLatitude: 45.148589; decimalLongitude: 9.252737; georeferencedBy: Silvia Stefanelli; georeferenceProtocol: GPS; **Identification:** identifiedBy: Paolo Audisio; dateIdentified: 2011

##### Distribution

Albania, Andorra, Austria, Belarus, Belgium, Bosnia and Herzegovina, Britain I., Bulgaria, Croatia, Czech Republic, Danish mainland, Estonia, European Turkey, Finland, French mainland, Germany, Greek mainland, Hungary, Ireland, Italian mainland, Kaliningrad Region, Latvia, Liechtenstein, Lithuania, Luxembourg, Macedonia, Moldova Republic of, Monaco, Northern Ireland, Norwegian mainland, Poland, Portuguese mainland, Romania, Russia Central, Russia East, Russia North, Russia Northwest, Russia South, Sardinia, Slovakia, Slovenia, Spanish mainland, Sweden, Switzerland, The Netherlands, Ukraine, Yugoslavia, Near East (Fauna Europaea 2013).

##### Notes

The larva develops in forests in mainly fermented sap and at the exit of galleries dug into the wood by other beetles. The adult sporadically attends the same microhabitat of larva, but it is also found on inflorescences and fermented fruit (Audisio 1993).

#### Epuraea marseuli

(Reitter, 1872)

Epuraea bickhardti Sainte-Claire Deville, 1906 – Fauna Europaea (2013)
Nitidula pusilla Illiger, 1798 – Fauna Europaea (2013)
Epuraea acuta Biström, 1977 – Fauna Europaea (2013)
Epuraea lenkorara Méquignon, 1945 – Fauna Europaea (2013)

##### Materials

- **Type status:** Other material. **Occurrence:** recordedBy: Silvia Stefanelli; individualCount: 3; lifeStage: adult; **Taxon:** taxonID: urn:lsid:faunaeur.org:taxname:377733; scientificName: Epuraea marseuli; order: Coleoptera; family: Nitidulidae; genus: Epuraea; scientificNameAuthorship: Reitter 1872; **Location:** country: Italy; stateProvince: Pavia; locality: SIC "Boschi di Vaccarizza" - V1; verbatimElevation: 62 m; verbatimCoordinates: 32T 519272E 4999526N; verbatimCoordinateSystem: UTM WGS 84; decimalLatitude: 45.148947; decimalLongitude: 9.245157; georeferencedBy: Silvia Stefanelli; georeferenceProtocol: GPS; **Identification:** identifiedBy: Paolo Audisio; dateIdentified: 2011

##### Distribution

Albania, Andorra, Austria, Belarus, Belgium, Bosnia and Herzegovina, Britain I., Bulgaria, Corsica, Croatia, Czech Republic, Danish mainland, Estonia, European Turkey, Finland, French mainland, Germany, Greek mainland, Hungary, Ireland, Italian mainland, Kaliningrad Region, Latvia, Liechtenstein, Lithuania, Luxembourg, Macedonia, Moldova Republic of, Northern Ireland, Norwegian mainland, Poland, Portuguese mainland, Romania, Russia Central, Russia East, Russia North, Russia Northwest, Russia South, Sicily, Slovakia, Slovenia, Spanish mainland, Sweden, Switzerland, The Netherlands, Ukraine, Yugoslavia, East Palaearctic, Near East, North Africa (Fauna Europaea 2013).

##### Notes

The species lives in conifer forests. The larva and adult are found mainly in the rotten wood of pine trees and on the fruiting bodies of Basidiomycetes that grows on this tree species. The adult is found on flowers and inflorescences of Dicotyledons, particularly during the spring and summer (Audisio 1993).

#### Silvanus bidentatus

(Fabricius, 1792)

##### Materials

- **Type status:** Other material. **Occurrence:** recordedBy: Silvia Stefanelli; individualCount: 2; lifeStage: adult; **Taxon:** taxonID: urn:lsid:faunaeur.org:taxname:124866; scientificName: Silvanus bidentatus; order: Coleoptera; family: Silvanidae; genus: Silvanus; scientificNameAuthorship: Fabricius 1792; **Location:** country: Italy; stateProvince: Pavia; locality: SIC "Boschi Siro Negri e Moriano" - BN1; verbatimElevation: 68 m; verbatimCoordinates: 32T 503258E 5007870N; verbatimCoordinateSystem: UTM WGS 84; decimalLatitude: 45.224312; decimalLongitude: 9.041499; georeferencedBy: Silvia Stefanelli; georeferenceProtocol: GPS; **Identification:** identifiedBy: Gianfranco Salvato; dateIdentified: 2011
- **Type status:** Other material. **Occurrence:** recordedBy: Silvia Stefanelli; individualCount: 3; lifeStage: adult; **Taxon:** taxonID: urn:lsid:faunaeur.org:taxname:124866; scientificName: Silvanus bidentatus; order: Coleoptera; family: Silvanidae; genus: Silvanus; scientificNameAuthorship: Fabricius 1792; **Location:** country: Italy; stateProvince: Pavia; locality: SIC "Boschi Siro Negri e Moriano" - BN21; verbatimElevation: 66 m; verbatimCoordinates: 32T 506342E 5005026N; verbatimCoordinateSystem: UTM WGS 84; decimalLatitude: 45.198691; decimalLongitude: 9.080746; georeferencedBy: Silvia Stefanelli; georeferenceProtocol: GPS; **Identification:** identifiedBy: Gianfranco Salvato; dateIdentified: 2011
- **Type status:** Other material. **Occurrence:** recordedBy: Silvia Stefanelli; individualCount: 6; lifeStage: adult; **Taxon:** taxonID: urn:lsid:faunaeur.org:taxname:124866; scientificName: Silvanus bidentatus; order: Coleoptera; family: Silvanidae; genus: Silvanus; scientificNameAuthorship: Fabricius 1792; **Location:** country: Italy; stateProvince: Pavia; locality: SIC "Boschi Siro Negri e Moriano" - BN5; verbatimElevation: 62 m; verbatimCoordinates: 32T 502886E 5008393N; verbatimCoordinateSystem: UTM WGS 84; decimalLatitude: 45.229029; decimalLongitude: 9.036770; georeferencedBy: Silvia Stefanelli; georeferenceProtocol: GPS; **Identification:** identifiedBy: Gianfranco Salvato; dateIdentified: 2011
- **Type status:** Other material. **Occurrence:** recordedBy: Silvia Stefanelli; individualCount: 4; lifeStage: adult; **Taxon:** taxonID: urn:lsid:faunaeur.org:taxname:124866; scientificName: Silvanus bidentatus; order: Coleoptera; family: Silvanidae; genus: Silvanus; scientificNameAuthorship: Fabricius 1792; **Location:** country: Italy; stateProvince: Pavia; locality: SIC "Boschi Siro Negri e Moriano" - BN10; verbatimElevation: 76 m; verbatimCoordinates: 32T 504479E 5006332N; verbatimCoordinateSystem: UTM WGS 84; decimalLatitude: 45.210461; decimalLongitude: 9.057038; georeferencedBy: Silvia Stefanelli; georeferenceProtocol: GPS; **Identification:** identifiedBy: Gianfranco Salvato; dateIdentified: 2011
- **Type status:** Other material. **Occurrence:** recordedBy: Silvia Stefanelli; individualCount: 5; lifeStage: adult; **Taxon:** taxonID: urn:lsid:faunaeur.org:taxname:124866; scientificName: Silvanus bidentatus; order: Coleoptera; family: Silvanidae; genus: Silvanus; scientificNameAuthorship: Fabricius 1792; **Location:** country: Italy; stateProvince: Pavia; locality: SIC "Boschi di Vaccarizza" - V1; verbatimElevation: 62 m; verbatimCoordinates: 32T 519272E 4999526N; verbatimCoordinateSystem: UTM WGS 84; decimalLatitude: 45.148947; decimalLongitude: 9.245157; georeferencedBy: Silvia Stefanelli; georeferenceProtocol: GPS; **Identification:** identifiedBy: Gianfranco Salvato; dateIdentified: 2011
- **Type status:** Other material. **Occurrence:** recordedBy: Silvia Stefanelli; individualCount: 2; lifeStage: adult; **Taxon:** taxonID: urn:lsid:faunaeur.org:taxname:124866; scientificName: Silvanus bidentatus; order: Coleoptera; family: Silvanidae; genus: Silvanus; scientificNameAuthorship: Fabricius 1792; **Location:** country: Italy; stateProvince: Pavia; locality: SIC "Boschi di Vaccarizza" - V2; verbatimElevation: 65 m; verbatimCoordinates: 32T 519868E 4999488N; verbatimCoordinateSystem: UTM WGS 84; decimalLatitude: 45.148589; decimalLongitude: 9.252737; georeferencedBy: Silvia Stefanelli; georeferenceProtocol: GPS; **Identification:** identifiedBy: Gianfranco Salvato; dateIdentified: 2011

##### Distribution

Austria, Belarus, Belgium, Britain I., Bulgaria, Corsica, Croatia, Czech Republic, Danish mainland, European Turkey, French mainland, Germany, Greek mainland, Hungary, Italian mainland, Lithuania, Poland, Portuguese mainland, Romania, Russia North, Russia Northwest, Russia South, Sardinia, Spanish mainland, Switzerland, Ukraine, Yugoslavia (Fauna Europaea 2013).

##### Notes

The species lives from the plains to the mountains, but is also found in urban areas. The larva develops under the bark of broadleaves such as oak, beech, elm, poplar, and hornbeam trees, and is rarely in conifers such as fir and pine trees. The adult appears from the end of April to July (Ratti 2007).

#### Silvanus unidentatus

(Olivier, 1790)

##### Materials

- **Type status:** Other material. **Occurrence:** recordedBy: Silvia Stefanelli; individualCount: 28; lifeStage: adult; **Taxon:** taxonID: urn:lsid:faunaeur.org:taxname:124868; scientificName: Silvanus unidentatus; order: Coleoptera; family: Silvanidae; genus: Silvanus; scientificNameAuthorship: Olivier 1790; **Location:** country: Italy; stateProvince: Pavia; locality: SIC "Boschi Siro Negri e Moriano" - BN1; verbatimElevation: 68 m; verbatimCoordinates: 32T 503258E 5007870N; verbatimCoordinateSystem: UTM WGS 84; decimalLatitude: 45.224312; decimalLongitude: 9.041499; georeferencedBy: Silvia Stefanelli; georeferenceProtocol: GPS; **Identification:** identifiedBy: Gianfranco Salvato; dateIdentified: 2011
- **Type status:** Other material. **Occurrence:** recordedBy: Silvia Stefanelli; individualCount: 37; lifeStage: adult; **Taxon:** taxonID: urn:lsid:faunaeur.org:taxname:124868; scientificName: Silvanus unidentatus; order: Coleoptera; family: Silvanidae; genus: Silvanus; scientificNameAuthorship: Olivier 1790; **Location:** country: Italy; stateProvince: Pavia; locality: SIC "Boschi Siro Negri e Moriano" - BN21; verbatimElevation: 66 m; verbatimCoordinates: 32T 506342E 5005026N; verbatimCoordinateSystem: UTM WGS 84; decimalLatitude: 45.198691; decimalLongitude: 9.080746; georeferencedBy: Silvia Stefanelli; georeferenceProtocol: GPS; **Identification:** identifiedBy: Gianfranco Salvato; dateIdentified: 2011
- **Type status:** Other material. **Occurrence:** recordedBy: Silvia Stefanelli; individualCount: 5; lifeStage: adult; **Taxon:** taxonID: urn:lsid:faunaeur.org:taxname:124868; scientificName: Silvanus unidentatus; order: Coleoptera; family: Silvanidae; genus: Silvanus; scientificNameAuthorship: Olivier 1790; **Location:** country: Italy; stateProvince: Pavia; locality: SIC "Boschi Siro Negri e Moriano" - BN5; verbatimElevation: 62 m; verbatimCoordinates: 32T 502949E 5008379N; verbatimCoordinateSystem: UTM WGS 84; decimalLatitude: 45.229029; decimalLongitude: 9.036770; georeferencedBy: Silvia Stefanelli; georeferenceProtocol: GPS; **Identification:** identifiedBy: Gianfranco Salvato; dateIdentified: 2011
- **Type status:** Other material. **Occurrence:** recordedBy: Silvia Stefanelli; individualCount: 4; lifeStage: adult; **Taxon:** taxonID: urn:lsid:faunaeur.org:taxname:124868; scientificName: Silvanus unidentatus; order: Coleoptera; family: Silvanidae; genus: Silvanus; scientificNameAuthorship: Olivier 1790; **Location:** country: Italy; stateProvince: Pavia; locality: SIC "Boschi Siro Negri e Moriano" - BN10; verbatimElevation: 76 m; verbatimCoordinates: 32T 504479E 5006332N; verbatimCoordinateSystem: UTM WGS 84; decimalLatitude: 45.210461; decimalLongitude: 9.057038; georeferencedBy: Silvia Stefanelli; georeferenceProtocol: GPS; **Identification:** identifiedBy: Gianfranco Salvato; dateIdentified: 2011
- **Type status:** Other material. **Occurrence:** recordedBy: Silvia Stefanelli; individualCount: 27; lifeStage: adult; **Taxon:** taxonID: urn:lsid:faunaeur.org:taxname:124868; scientificName: Silvanus unidentatus; order: Coleoptera; family: Silvanidae; genus: Silvanus; scientificNameAuthorship: Olivier 1790; **Location:** country: Italy; stateProvince: Pavia; locality: SIC "Boschi di Vaccarizza" - V1; verbatimElevation: 62 m; verbatimCoordinates: 32T 519272E 4999526N; verbatimCoordinateSystem: UTM WGS 84; decimalLatitude: 45.148947; decimalLongitude: 9.245157; georeferencedBy: Silvia Stefanelli; georeferenceProtocol: GPS; **Identification:** identifiedBy: Gianfranco Salvato; dateIdentified: 2011
- **Type status:** Other material. **Occurrence:** recordedBy: Silvia Stefanelli; individualCount: 5; lifeStage: adult; **Taxon:** taxonID: urn:lsid:faunaeur.org:taxname:124868; scientificName: Silvanus unidentatus; order: Coleoptera; family: Silvanidae; genus: Silvanus; scientificNameAuthorship: Olivier 1790; **Location:** country: Italy; stateProvince: Pavia; locality: SIC "Boschi di Vaccarizza" - V2; verbatimElevation: 65 m; verbatimCoordinates: 32T 519868E 4999488N; verbatimCoordinateSystem: UTM WGS 84; decimalLatitude: 45.148589; decimalLongitude: 9.252737; georeferencedBy: Silvia Stefanelli; georeferenceProtocol: GPS; **Identification:** identifiedBy: Gianfranco Salvato; dateIdentified: 2011

##### Distribution

Austria, Belarus, Belgium, Britain I., Bulgaria, Corsica, Croatia, Czech Republic, Danish mainland, European Turkey, French mainland, Germany, Greek mainland, Hungary, Italian mainland, Lithuania, Poland, Portuguese mainland, Romania, Russia North, Russia Northwest, Russia South, Sardinia, Spanish mainland, Switzerland, Ukraine, Yugoslavia (Fauna Europaea 2013).

##### Notes

The species is autochtonous for Italy and is the most widespread of the genus. It lives in forests, but is also found in isolated dead trees. Usually it is gregarious, and it is found under the bark of various broadleaves such as hornbeam, chestnut, beech, poplar, oak, robinia, willow, elm, and fruits trees. It is rarely found in conifers such as fir and pine trees. Both larva and adults are often found with the Silvanidae *Uleiota planata*. The adult flies during the night (Ratti 2007).

#### Uleiota planata

(Linneaus, 1761)

##### Materials

- **Type status:** Other material. **Occurrence:** recordedBy: Silvia Stefanelli; individualCount: 10; lifeStage: adult; **Taxon:** taxonID: urn:lsid:faunaeur.org:taxname:124870; scientificName: Uleiota planata; order: Coleoptera; family: Silvanidae; genus: Uleiota ; scientificNameAuthorship: Linnaeus 1761; **Location:** country: Italy; stateProvince: Pavia; locality: SIC "Boschi Siro Negri e Moriano" - BN1; verbatimElevation: 68 m; verbatimCoordinates: 32T 503258E 5007870N; verbatimCoordinateSystem: UTM WGS 84; decimalLatitude: 45.224312; decimalLongitude: 9.041499; georeferencedBy: Silvia Stefanelli; georeferenceProtocol: GPS; **Identification:** identifiedBy: Gianfranco Salvato; dateIdentified: 2011
- **Type status:** Other material. **Occurrence:** recordedBy: Silvia Stefanelli; individualCount: 16; lifeStage: adult; **Taxon:** taxonID: urn:lsid:faunaeur.org:taxname:124870; scientificName: Uleiota planata; order: Coleoptera; family: Silvanidae; genus: Uleiota ; scientificNameAuthorship: Linnaeus 1761; **Location:** country: Italy; stateProvince: Pavia; locality: SIC "Boschi Siro Negri e Moriano" - BN21; verbatimElevation: 66 m; verbatimCoordinates: 32T 506342E 5005026N; verbatimCoordinateSystem: UTM WGS 84; decimalLatitude: 45.198691; decimalLongitude: 9.080746; georeferencedBy: Silvia Stefanelli; georeferenceProtocol: GPS; **Identification:** identifiedBy: Gianfranco Salvato; dateIdentified: 2011
- **Type status:** Other material. **Occurrence:** recordedBy: Silvia Stefanelli; individualCount: 11; lifeStage: adult; **Taxon:** taxonID: urn:lsid:faunaeur.org:taxname:124870; scientificName: Uleiota planata; order: Coleoptera; family: Silvanidae; genus: Uleiota ; scientificNameAuthorship: Linnaeus 1761; **Location:** country: Italy; stateProvince: Pavia; locality: SIC "Boschi Siro Negri e Moriano" - BN5; verbatimElevation: 62 m; verbatimCoordinates: 32T 502886E 5008393N; verbatimCoordinateSystem: UTM WGS 84; decimalLatitude: 45.229029; decimalLongitude: 9.036770; georeferencedBy: Silvia Stefanelli; georeferenceProtocol: GPS; **Identification:** identifiedBy: Gianfranco Salvato; dateIdentified: 2011
- **Type status:** Other material. **Occurrence:** recordedBy: Silvia Stefanelli; individualCount: 13; lifeStage: adult; **Taxon:** taxonID: urn:lsid:faunaeur.org:taxname:124870; scientificName: Uleiota planata; order: Coleoptera; family: Silvanidae; genus: Uleiota ; scientificNameAuthorship: Linnaeus 1761; **Location:** country: Italy; stateProvince: Pavia; locality: SIC "Boschi Siro Negri e Moriano" - BN10; verbatimElevation: 76 m; verbatimCoordinates: 32T 504479E 5006332N; verbatimCoordinateSystem: UTM WGS 84; decimalLatitude: 45.210461; decimalLongitude: 9.057038; georeferencedBy: Silvia Stefanelli; georeferenceProtocol: GPS; **Identification:** identifiedBy: Gianfranco Salvato; dateIdentified: 2011
- **Type status:** Other material. **Occurrence:** recordedBy: Silvia Stefanelli; individualCount: 23; lifeStage: adult; **Taxon:** taxonID: urn:lsid:faunaeur.org:taxname:124870; scientificName: Uleiota planata; order: Coleoptera; family: Silvanidae; genus: Uleiota ; scientificNameAuthorship: Linnaeus 1761; **Location:** country: Italy; stateProvince: Pavia; locality: SIC "Boschi di Vaccarizza" - V1; verbatimElevation: 62 m; verbatimCoordinates: 32T 519272E 4999526N; verbatimCoordinateSystem: UTM WGS 84; decimalLatitude: 45.148947; decimalLongitude: 9.245157; georeferencedBy: Silvia Stefanelli; georeferenceProtocol: GPS; **Identification:** identifiedBy: Gianfranco Salvato; dateIdentified: 2011
- **Type status:** Other material. **Occurrence:** recordedBy: Silvia Stefanelli; individualCount: 4; lifeStage: adult; **Taxon:** taxonID: urn:lsid:faunaeur.org:taxname:124870; scientificName: Uleiota planata; order: Coleoptera; family: Silvanidae; genus: Uleiota ; scientificNameAuthorship: Linnaeus 1761; **Location:** country: Italy; stateProvince: Pavia; locality: SIC "Boschi di Vaccarizza" - V2; verbatimElevation: 65 m; verbatimCoordinates: 32T 519868E 4999488N; verbatimCoordinateSystem: UTM WGS 84; decimalLatitude: 45.148589; decimalLongitude: 9.252737; georeferencedBy: Silvia Stefanelli; georeferenceProtocol: GPS; **Identification:** identifiedBy: Gianfranco Salvato; dateIdentified: 2011

##### Distribution

Austria, Belarus, Belgium, Britain I., Bulgaria, Corsica, Croatia, Czech Republic, Danish mainland, European Turkey, French mainland, Germany, Greek mainland, Hungary, Italian mainland, Lithuania, Poland, Portuguese mainland, Romania, Russia North, Russia Northwest, Russia South, Sardinia, Spanish mainland, Switzerland, Ukraine, Yugoslavia (Fauna Europaea 2013).

##### Notes

The species is native for Italy and is found both in igrophilus, mesophilic, and mesotermophilic forests. The larva develops under the bark of broadleaves deadwood such as chestnut, beech, poplar, oak, robinia, willow, elm, alder, birch, and fruits trees, but is also found in conifers. The adult appears mainly in July (Ratti 2007).

#### Diaperis boleti

(Linneaus, 1758)

##### Materials

- **Type status:** Other material. **Occurrence:** recordedBy: Silvia Stefanelli; individualCount: 1; lifeStage: adult; **Taxon:** taxonID: urn:lsid:faunaeur.org:taxname:282077; scientificName: Diaperis boleti; order: Coleoptera; family: Tenebrionidae; genus: Diaperis; scientificNameAuthorship: Linnaeus 1758; **Location:** country: Italy; stateProvince: Pavia; locality: SIC "Boschi Siro Negri e Moriano" - BN1; verbatimElevation: 68 m; verbatimCoordinates: 32T 503258E 5007870N; verbatimCoordinateSystem: UTM WGS 84; decimalLatitude: 45.224312; decimalLongitude: 9.041499; georeferencedBy: Silvia Stefanelli; georeferenceProtocol: GPS; **Identification:** identifiedBy: Giuseppe Carpaneto; dateIdentified: 2001
- **Type status:** Other material. **Occurrence:** recordedBy: Silvia Stefanelli; individualCount: 1; lifeStage: adult; **Taxon:** taxonID: urn:lsid:faunaeur.org:taxname:282077; scientificName: Diaperis boleti; order: Coleoptera; family: Tenebrionidae; genus: Diaperis; scientificNameAuthorship: Linnaeus 1758; **Location:** country: Italy; stateProvince: Pavia; locality: SIC "Boschi Siro Negri e Moriano" - BN10; verbatimElevation: 76 m; verbatimCoordinates: 32T 504479E 5006332N; verbatimCoordinateSystem: UTM WGS 84; decimalLatitude: 45.210461; decimalLongitude: 9.057038; georeferencedBy: Silvia Stefanelli; georeferenceProtocol: GPS; **Identification:** identifiedBy: Giuseppe Carpaneto; dateIdentified: 2011

##### Distribution

Albania, Austria, Belgium, Bosnia and Herzegovina, Britain I., Bulgaria, Corsica, Croatia, Czech Republic, Danish mainland, Estonia, Finland, French mainland, Germany, Greek mainland, Hungary, Italian mainland, Latvia, Lithuania, Luxembourg, Macedonia, Norwegian mainland, Poland, Romania, Russia North, Sardinia, Sicily, Slovakia, Sweden (Fauna Europaea 2013).

##### Notes

The species is common locally and is associated with tree fungi. The larva develops in various polypore fungi such as *Polyporus squamosus*, *Laetiporus sulphureus*, and *Piptoporus betulinus* (Hůrka 2005).

#### Hypophloeus bicolor

(Oliver, 1790)

##### Materials

- **Type status:** Other material. **Occurrence:** recordedBy: Silvia Stefanelli; individualCount: 2; lifeStage: adult; **Taxon:** taxonID: urn:lsid:faunaeur.org:taxname:282083; scientificName: Hypophloeus bicolor; order: Coleoptera; family: Tenebrionidae; genus: Hypophloeus; scientificNameAuthorship: Olivier 1790; **Location:** country: Italy; stateProvince: Pavia; locality: SIC "Boschi Siro Negri e Moriano" - BN21; verbatimElevation: 66 m; verbatimCoordinates: 32T 506342E 5005026N; verbatimCoordinateSystem: UTM WGS 84; decimalLatitude: 45.198691; decimalLongitude: 9.080746; georeferencedBy: Silvia Stefanelli; georeferenceProtocol: GPS; **Identification:** identifiedBy: Giuseppe Carpaneto; dateIdentified: 2011
- **Type status:** Other material. **Occurrence:** recordedBy: Silvia Stefanelli; individualCount: 3; lifeStage: adult; **Taxon:** taxonID: urn:lsid:faunaeur.org:taxname:282083; scientificName: Hypophloeus bicolor; order: Coleoptera; family: Tenebrionidae; genus: Hypophloeus; scientificNameAuthorship: Olivier 1790; **Location:** country: Italy; stateProvince: Pavia; locality: SIC "Boschi di Vaccarizza" - V2; verbatimElevation: 65 m; verbatimCoordinates: 32T 519868E 4999488N; verbatimCoordinateSystem: UTM WGS 84; decimalLatitude: 45.148589; decimalLongitude: 9.252737; georeferencedBy: Silvia Stefanelli; georeferenceProtocol: GPS; **Identification:** identifiedBy: Giuseppe Carpaneto; dateIdentified: 2011

##### Distribution

Albania, Austria, Belgium, Bosnia and Herzegovina, Britain I., Bulgaria, Corsica, Czech Republic, Danish mainland, Estonia, Finland, French mainland, Germany, Greek mainland, Hungary, Italian mainland, Latvia, Lithuania, Luxembourg, Malta, Norwegian mainland, Poland, Romania, Russia North, Sardinia, Sicily, Slovakia, Sweden (Fauna Europaea 2013).

##### Notes

The species is commensal in burrows of the bark beetles *Scolytus scolytus* and *Scolytus multistriatus*. It usually lives in elm and oak trees on mainly fungi and detritus; sometimes it is also associated with *Daldinia concentrica* on old ash and with *Polyporus squamosus* (Alexander 2002).

#### Hypophloeus fasciatus

(Fabricius, 1790)

##### Materials

- **Type status:** Other material. **Occurrence:** recordedBy: Silvia Stefanelli; individualCount: 3; lifeStage: adult; **Taxon:** taxonID: urn:lsid:faunaeur.org:taxname:282085; scientificName: Hypophloeus fasciatus; order: Coleoptera; family: Tenebrionidae; genus: Hypophloeus; scientificNameAuthorship: Fabricius 1790; **Location:** country: Italy; stateProvince: Pavia; locality: SIC "Boschi Siro Negri e Moriano" - BN10; verbatimElevation: 76 m; verbatimCoordinates: 32T 504479E 5006332N; verbatimCoordinateSystem: UTM WGS 84; decimalLatitude: 45.210461; decimalLongitude: 9.057038; georeferencedBy: Silvia Stefanelli; georeferenceProtocol: GPS; **Identification:** identifiedBy: Giuseppe Carpaneto; dateIdentified: 2011

##### Distribution

Albania, Austria, Bulgaria, Corsica, Crete, Croatia, Czech Republic, Danish mainland, Estonia, French mainland, Germany, Greek mainland, Hungary, Italian mainland, Poland, Romania, Sardinia, Sicily, Slovakia, Sweden (Fauna Europaea 2013).

##### Notes

The larva develops together with other bark beetles living under the bark of broadleaves, especially oaks (Hůrka 2005).

#### Hypophloeus unicolor

(Piller & Mitterpacher, 1783)

##### Materials

- **Type status:** Other material. **Occurrence:** recordedBy: Silvia Stefanelli; individualCount: 3; lifeStage: adult; **Taxon:** taxonID: urn:lsid:faunaeur.org:taxname:282096; scientificName: Hypophloeus unicolor; order: Coleoptera; family: Tenebrionidae; genus: Hypophloeus; scientificNameAuthorship: Piller & Mitterpacher 1783; **Location:** country: Italy; stateProvince: Pavia; locality: SIC "Boschi di Vaccarizza" - V1; verbatimElevation: 62 m; verbatimCoordinates: 32T 519272E 4999526N; verbatimCoordinateSystem: UTM WGS 84; decimalLatitude: 45.148947; decimalLongitude: 9.245157; georeferencedBy: Silvia Stefanelli; georeferenceProtocol: GPS; **Identification:** identifiedBy: Giuseppe Carpaneto; dateIdentified: 2011
- **Type status:** Other material. **Occurrence:** recordedBy: Silvia Stefanelli; individualCount: 26; lifeStage: adult; **Taxon:** taxonID: urn:lsid:faunaeur.org:taxname:282096; scientificName: Hypophloeus unicolor; order: Coleoptera; family: Tenebrionidae; genus: Hypophloeus; scientificNameAuthorship: Piller & Mitterpacher 1783; **Location:** country: Italy; stateProvince: Pavia; locality: SIC "Boschi di Vaccarizza" - V2; verbatimElevation: 65 m; verbatimCoordinates: 32T 519868E 4999488N; verbatimCoordinateSystem: UTM WGS 84; decimalLatitude: 45.148589; decimalLongitude: 9.252737; georeferencedBy: Silvia Stefanelli; georeferenceProtocol: GPS; **Identification:** identifiedBy: Giuseppe Carpaneto; dateIdentified: 2011

##### Distribution

Albania, Austria, Belgium, Bosnia and Herzegovina, Britain I., Bulgaria, Corsica, Croatia, Czech Republic, Danish mainland, Estonia, French mainland, Germany, Greek mainland, Hungary, Italian mainland, Latvia, Lithuania, Malta, Norwegian mainland, Poland, Sardinia, Sicily, Slovakia, Sweden, Yugoslavia (Fauna Europaea 2013).

##### Notes

The species develops in freshly the dead wood of birch, beech, and oak trees. It is probably a predator of the larvae of the beetle *Hylecoetus* and other wood borers (Alexander 2002).

#### Platydema violaceum

(Fabricius, 1790)

##### Materials

- **Type status:** Other material. **Occurrence:** recordedBy: Silvia Stefanelli; individualCount: 1; lifeStage: adult; **Taxon:** taxonID: urn:lsid:faunaeur.org:taxname:282066; scientificName: Platydema violaceum; order: Coleoptera; family: Tenebrionidae; genus: Platydema; scientificNameAuthorship: Fabricius 1790; **Location:** country: Italy; stateProvince: Pavia; locality: SIC "Boschi Siro Negri e Moriano" - BN21; verbatimElevation: 66 m; verbatimCoordinates: 32T 506342E 5005026N; verbatimCoordinateSystem: UTM WGS 84; decimalLatitude: 45.198691; decimalLongitude: 9.080746; georeferencedBy: Silvia Stefanelli; georeferenceProtocol: GPS; **Identification:** identifiedBy: Giuseppe Carpaneto; dateIdentified: 2011

##### Distribution

Albania, Austria, Belgium, Bosnia and Herzegovina, Britain I., Bulgaria, Croatia, Czech Republic, Danish mainland, Estonia, Finland, French mainland, Germany, Greek mainland, Hungary, Italian mainland, Latvia, Lithuania, Luxembourg, Poland, Romania, Slovakia, Sweden (Fauna Europaea 2013).

##### Notes

The species lives under the bark of old broadleaves invaded by fungi, especially oak and beech trees. It is also found under the bark of black elms *Sambucus nigra* invaded by the fungus *Hirneola auricola-judae* (Hůrka 2005).

#### Scaphidema metallicum

(Fabricius, 1792)

##### Materials

- **Type status:** Other material. **Occurrence:** recordedBy: Silvia Stefanelli; individualCount: 4; lifeStage: adult; **Taxon:** taxonID: urn:lsid:faunaeur.org:taxname:281913; scientificName: Scaphidema metallicum; order: Coleoptera; family: Tenebrionidae; genus: Scaphidema; scientificNameAuthorship: Fabricius 1792; **Location:** country: Italy; stateProvince: Pavia; locality: SIC "Boschi Siro Negri e Moriano" - BN5; verbatimElevation: 62 m; verbatimCoordinates: 32T 502886E 5008393N; verbatimCoordinateSystem: UTM WGS 84; decimalLatitude: 45.229029; decimalLongitude: 9.036770; georeferencedBy: Silvia Stefanelli; georeferenceProtocol: GPS; **Identification:** identifiedBy: Giuseppe Carpaneto; dateIdentified: 2011
- **Type status:** Other material. **Occurrence:** recordedBy: Silvia Stefanelli; individualCount: 2; lifeStage: adult; **Taxon:** taxonID: urn:lsid:faunaeur.org:taxname:281913; scientificName: Scaphidema metallicum; order: Coleoptera; family: Tenebrionidae; genus: Scaphidema; scientificNameAuthorship: Fabricius 1792; **Location:** country: Italy; stateProvince: Pavia; locality: SIC "Boschi di Vaccarizza" - V1; verbatimElevation: 62 m; verbatimCoordinates: 32T 519272E 4999526N; verbatimCoordinateSystem: UTM WGS 84; decimalLatitude: 45.148947; decimalLongitude: 9.245157; georeferencedBy: Silvia Stefanelli; georeferenceProtocol: GPS; **Identification:** identifiedBy: Giuseppe Carpaneto; dateIdentified: 2011
- **Type status:** Other material. **Occurrence:** recordedBy: Silvia Stefanelli; individualCount: 4; lifeStage: adult; **Taxon:** taxonID: urn:lsid:faunaeur.org:taxname:281913; scientificName: Scaphidema metallicum; order: Coleoptera; family: Tenebrionidae; genus: Scaphidema; scientificNameAuthorship: Fabricius 1792; **Location:** country: Italy; stateProvince: Pavia; locality: SIC "Boschi di Vaccarizza" - V2; verbatimElevation: 65 m; verbatimCoordinates: 32T 519868E 4999488N; verbatimCoordinateSystem: UTM WGS 84; decimalLatitude: 45.148589; decimalLongitude: 9.252737; georeferencedBy: Silvia Stefanelli; georeferenceProtocol: GPS; **Identification:** identifiedBy: Giuseppe Carpaneto; dateIdentified: 2011

##### Distribution

Albania, Austria, Belgium, Bosnia and Herzegovina, Britain I., Bulgaria, Croatia, Czech Republic, Danish mainland, Estonia, Finland, French mainland, Germany, Greek mainland, Italian mainland, Latvia, Lithuania, Luxembourg, Norwegian mainland, Poland, Russia North, Slovakia, Sweden, Yugoslavia (Fauna Europaea 2013).

##### Notes

The species is common. The larva develops in the rotting wood of broadleaves invaded by fungi (Fauna Europaea 2013).

#### Stenomax aeneus

(Scopoli, 1763)

Tenebrio aenus Scopoli, 1763 – Fauna Europaea (2013)

##### Materials

- **Type status:** Other material. **Occurrence:** recordedBy: Silvia Stefanelli; individualCount: 21; lifeStage: adult; **Taxon:** taxonID: urn:lsid:faunaeur.org:taxname:281396; scientificName: Stenomax aeneus; order: Coleoptera; family: Tenebrionidae; genus: Stenomax; scientificNameAuthorship: Scopoli 1763; **Location:** country: Italy; stateProvince: Pavia; locality: SIC "Boschi Siro Negri e Moriano" - BN21; verbatimElevation: 66 m; verbatimCoordinates: 32T 506342E 5005026N; verbatimCoordinateSystem: UTM WGS 84; decimalLatitude: 45.198691; decimalLongitude: 9.080746; georeferencedBy: Silvia Stefanelli; georeferenceProtocol: GPS; **Identification:** identifiedBy: Giuseppe Carpaneto; dateIdentified: 2011
- **Type status:** Other material. **Occurrence:** recordedBy: Silvia Stefanelli; individualCount: 3; lifeStage: adult; **Taxon:** taxonID: urn:lsid:faunaeur.org:taxname:281396; scientificName: Stenomax aeneus; order: Coleoptera; family: Tenebrionidae; genus: Stenomax; scientificNameAuthorship: Scopoli 1763; **Location:** country: Italy; stateProvince: Pavia; locality: SIC "Boschi Siro Negri e Moriano" - BN10; verbatimElevation: 76 m; verbatimCoordinates: 32T 504479E 5006332N; verbatimCoordinateSystem: UTM WGS 84; decimalLatitude: 45.210461; decimalLongitude: 9.057038; georeferencedBy: Silvia Stefanelli; georeferenceProtocol: GPS; **Identification:** identifiedBy: Giuseppe Carpaneto; dateIdentified: 2011
- **Type status:** Other material. **Occurrence:** recordedBy: Silvia Stefanelli; individualCount: 16; lifeStage: adult; **Taxon:** taxonID: urn:lsid:faunaeur.org:taxname:281396; scientificName: Stenomax aeneus; order: Coleoptera; family: Tenebrionidae; genus: Stenomax; scientificNameAuthorship: Scopoli 1763; **Location:** country: Italy; stateProvince: Pavia; locality: SIC "Boschi di Vaccarizza" - V1; verbatimElevation: 62 m; verbatimCoordinates: 32T 519272E 4999526N; verbatimCoordinateSystem: UTM WGS 84; decimalLatitude: 45.148947; decimalLongitude: 9.245157; georeferencedBy: Silvia Stefanelli; georeferenceProtocol: GPS; **Identification:** identifiedBy: Giuseppe Carpaneto; dateIdentified: 2011
- **Type status:** Other material. **Occurrence:** recordedBy: Silvia Stefanelli; individualCount: 2; lifeStage: adult; **Taxon:** taxonID: urn:lsid:faunaeur.org:taxname:281396; scientificName: Stenomax aeneus; order: Coleoptera; family: Tenebrionidae; genus: Stenomax; scientificNameAuthorship: Scopoli 1763; **Location:** country: Italy; stateProvince: Pavia; locality: SIC "Boschi di Vaccarizza" - V2; verbatimElevation: 65 m; verbatimCoordinates: 32T 519868E 4999488N; verbatimCoordinateSystem: UTM WGS 84; decimalLatitude: 45.148589; decimalLongitude: 9.252737; georeferencedBy: Silvia Stefanelli; georeferenceProtocol: GPS; **Identification:** identifiedBy: Giuseppe Carpaneto; dateIdentified: 2011

##### Distribution

Albania, Austria, Bosnia and Herzegovina, Bulgaria, Croatia, Czech Republic, European Turkey, French mainland, Germany, Greek mainland, Hungary, Italian mainland, Macedonia, Poland, Romania, Slovakia, Slovenia, Switzerland, Yugoslavia (Fauna Europaea 2013).

#### Uloma culinaris

(Linneaus, 1758)

Tenebrio culinaris Linneaus, 1758 – Fauna Europaea (2013)

##### Materials

- **Type status:** Other material. **Occurrence:** recordedBy: Silvia Stefanelli; individualCount: 10; lifeStage: adult; **Taxon:** taxonID: urn:lsid:faunaeur.org:taxname:281973; scientificName: Uloma culinaris; order: Coleoptera; family: Tenebrionidae; genus: Uloma; scientificNameAuthorship: Linnaeus 1758; **Location:** country: Italy; stateProvince: Pavia; locality: SIC "Boschi Siro Negri e Moriano" - BN1; verbatimElevation: 68 m; verbatimCoordinates: 32T 503258E 5007870N; verbatimCoordinateSystem: UTM WGS 84; decimalLatitude: 45.224312; decimalLongitude: 9.041499; georeferencedBy: Silvia Stefanelli; georeferenceProtocol: GPS; **Identification:** identifiedBy: Giuseppe Carpaneto; dateIdentified: 2011
- **Type status:** Other material. **Occurrence:** recordedBy: Silvia Stefanelli; individualCount: 1; lifeStage: adult; **Taxon:** taxonID: urn:lsid:faunaeur.org:taxname:281973; scientificName: Uloma culinaris; order: Coleoptera; family: Tenebrionidae; genus: Uloma; scientificNameAuthorship: Linnaeus 1758; **Location:** country: Italy; stateProvince: Pavia; locality: SIC "Boschi Siro Negri e Moriano" - BN21; verbatimElevation: 66 m; verbatimCoordinates: 32T 506342E 5005026N; verbatimCoordinateSystem: UTM WGS 84; decimalLatitude: 45.198691; decimalLongitude: 9.080746; georeferencedBy: Silvia Stefanelli; georeferenceProtocol: GPS; **Identification:** identifiedBy: Giuseppe Carpaneto; dateIdentified: 2011
- **Type status:** Other material. **Occurrence:** recordedBy: Silvia Stefanelli; individualCount: 14; lifeStage: adult; **Taxon:** taxonID: urn:lsid:faunaeur.org:taxname:281973; scientificName: Uloma culinaris; order: Coleoptera; family: Tenebrionidae; genus: Uloma; scientificNameAuthorship: Linnaeus 1758; **Location:** country: Italy; stateProvince: Pavia; locality: SIC "Boschi Siro Negri e Moriano" - BN5; verbatimElevation: 62 m; verbatimCoordinates: 32T 502886E 5008393N; verbatimCoordinateSystem: UTM WGS 84; decimalLatitude: 45.229029; decimalLongitude: 9.036770; georeferencedBy: Silvia Stefanelli; georeferenceProtocol: GPS; **Identification:** identifiedBy: Giuseppe Carpaneto; dateIdentified: 2011
- **Type status:** Other material. **Occurrence:** recordedBy: Silvia Stefanelli; individualCount: 5; lifeStage: adult; **Taxon:** taxonID: urn:lsid:faunaeur.org:taxname:281973; scientificName: Uloma culinaris; order: Coleoptera; family: Tenebrionidae; genus: Uloma; scientificNameAuthorship: Linnaeus 1758; **Location:** country: Italy; stateProvince: Pavia; locality: SIC "Boschi Siro Negri e Moriano" - BN10; verbatimElevation: 76 m; verbatimCoordinates: 32T 504479E 5006332N; verbatimCoordinateSystem: UTM WGS 84; decimalLatitude: 45.210461; decimalLongitude: 9.057038; georeferencedBy: Silvia Stefanelli; georeferenceProtocol: GPS; **Identification:** identifiedBy: Giuseppe Carpaneto; dateIdentified: 2011
- **Type status:** Other material. **Occurrence:** recordedBy: Silvia Stefanelli; individualCount: 18; lifeStage: adult; **Taxon:** taxonID: urn:lsid:faunaeur.org:taxname:281973; scientificName: Uloma culinaris; order: Coleoptera; family: Tenebrionidae; genus: Uloma; scientificNameAuthorship: Linnaeus 1758; **Location:** country: Italy; stateProvince: Pavia; locality: SIC "Boschi di Vaccarizza" - V1; verbatimElevation: 62 m; verbatimCoordinates: 32T 519272E 4999526N; verbatimCoordinateSystem: UTM WGS 84; decimalLatitude: 45.148947; decimalLongitude: 9.245157; georeferencedBy: Silvia Stefanelli; georeferenceProtocol: GPS; **Identification:** identifiedBy: Giuseppe Carpaneto; dateIdentified: 2011
- **Type status:** Other material. **Occurrence:** recordedBy: Silvia Stefanelli; individualCount: 3; lifeStage: adult; **Taxon:** taxonID: urn:lsid:faunaeur.org:taxname:281973; scientificName: Uloma culinaris; order: Coleoptera; family: Tenebrionidae; genus: Uloma; scientificNameAuthorship: Linnaeus 1758; **Location:** country: Italy; stateProvince: Pavia; locality: SIC "Boschi di Vaccarizza" - V2; verbatimElevation: 65 m; verbatimCoordinates: 32T 519868E 4999488N; verbatimCoordinateSystem: UTM WGS 84; decimalLatitude: 45.148589; decimalLongitude: 9.252737; georeferencedBy: Silvia Stefanelli; georeferenceProtocol: GPS; **Identification:** identifiedBy: Giuseppe Carpaneto; dateIdentified: 2011

##### Distribution

Albania, Austria, Belgium, Bosnia and Herzegovina, Bulgaria, Corsica, Crete, Croatia, Czech Republic, Danish mainland, Estonia, French mainland, Germany, Greek mainland, Hungary, Italian mainland, Latvia, Lithuania, Luxembourg, Norwegian mainland, Poland, Romania, Sicily, Slovakia, Sweden, Yugoslavia (Fauna Europaea 2013).

##### Notes

The larva and adults live together under the bark and in the rotten wood of conifers and broadleaves (Hůrka 2005).

#### Bitoma crenata

(Fabricius, 1775)

##### Materials

- **Type status:** Other material. **Occurrence:** recordedBy: Silvia Stefanelli; individualCount: 11; lifeStage: adult; **Taxon:** taxonID: urn:lsid:faunaeur.org:taxname:127036; scientificName: Bitoma crenata; order: Coleoptera; family: Zopheridae; genus: Bitoma; scientificNameAuthorship: Fabricius 1775; **Location:** country: Italy; stateProvince: Pavia; locality: SIC "Boschi Siro Negri e Moriano" - BN1; verbatimElevation: 68 m; verbatimCoordinates: 32T 503258E 5007870N; verbatimCoordinateSystem: UTM WGS 84; decimalLatitude: 45.224312; decimalLongitude: 9.041499; georeferencedBy: Silvia Stefanelli; georeferenceProtocol: GPS; **Identification:** identifiedBy: Claudio Canepari; dateIdentified: 2011
- **Type status:** Other material. **Occurrence:** recordedBy: Silvia Stefanelli; individualCount: 20; lifeStage: adult; **Taxon:** taxonID: urn:lsid:faunaeur.org:taxname:127036; scientificName: Bitoma crenata; order: Coleoptera; family: Zopheridae; genus: Bitoma; scientificNameAuthorship: Fabricius 1775; **Location:** country: Italy; stateProvince: Pavia; locality: SIC "Boschi Siro Negri e Moriano" - BN21; verbatimElevation: 66 m; verbatimCoordinates: 32T 506342E 5005026N; verbatimCoordinateSystem: UTM WGS 84; decimalLatitude: 45.198691; decimalLongitude: 9.080746; georeferencedBy: Silvia Stefanelli; georeferenceProtocol: GPS; **Identification:** identifiedBy: Claudio Canepari; dateIdentified: 2011
- **Type status:** Other material. **Occurrence:** recordedBy: Silvia Stefanelli; individualCount: 1; lifeStage: adult; **Taxon:** taxonID: urn:lsid:faunaeur.org:taxname:127036; scientificName: Bitoma crenata; order: Coleoptera; family: Zopheridae; genus: Bitoma; scientificNameAuthorship: Fabricius 1775; **Location:** country: Italy; stateProvince: Pavia; locality: SIC "Boschi Siro Negri e Moriano" - BN5; verbatimElevation: 62 m; verbatimCoordinates: 32T 502886E 5008393N; verbatimCoordinateSystem: UTM WGS 84; decimalLatitude: 45.229029; decimalLongitude: 9.036770; georeferencedBy: Silvia Stefanelli; georeferenceProtocol: GPS; **Identification:** identifiedBy: Claudio Canepari; dateIdentified: 2011

##### Distribution

Austria, Azores, Belarus, Belgium, Bosnia and Herzegovina, Britain I., Bulgaria, Corsica, Crete, Croatia, Cyprus, Danish mainland, European Turkey, Faroe Is., Finland, French mainland, Germany, Greek mainland, Hungary, Italian mainland, Latvia, Lithuania, Macedonia, Madeira, Malta, Moldova Republic of, Norwegian mainland, Poland, Portuguese mainland, Romania, Russia Central, Russia East, Russia North, Russia Northwest, Russia South, Sardinia, Sicily, Slovakia, Slovenia, Spanish mainland, Sweden, Switzerland, The Netherlands, Ukraine, Yugoslavia, Near East (Fauna Europaea 2013).

##### Notes

The species lives in ancient wood pastures. It lives under the bark of beech and oak tree deadwood in the early stages of decay and still sappy. It can also be found on birch, horse chestnut, and sycamore trees. The larva and adults are gregarious (Alexander 2002, Hůrka 2005).

#### Colobicus hirtus

(Rossi, 1790)

##### Materials

- **Type status:** Other material. **Occurrence:** recordedBy: Silvia Stefanelli; individualCount: 1; lifeStage: adult; **Taxon:** taxonID: urn:lsid:faunaeur.org:taxname:127034; scientificName: Colobicus hirtus; order: Coleoptera; family: Zopheridae; genus: Colobicus; scientificNameAuthorship: Rossi 1790; **Location:** country: Italy; stateProvince: Pavia; locality: SIC "Boschi Siro Negri e Moriano" - BN21; verbatimElevation: 66 m; verbatimCoordinates: 32T 506342E 5005026N; verbatimCoordinateSystem: UTM WGS 84; decimalLatitude: 45.198691; decimalLongitude: 9.080746; georeferencedBy: Silvia Stefanelli; georeferenceProtocol: GPS; **Identification:** identifiedBy: Claudio Canepari; dateIdentified: 2011
- **Type status:** Other material. **Occurrence:** recordedBy: Silvia Stefanelli; individualCount: 1; lifeStage: adult; **Taxon:** taxonID: urn:lsid:faunaeur.org:taxname:127034; scientificName: Colobicus hirtus; order: Coleoptera; family: Zopheridae; genus: Colobicus; scientificNameAuthorship: Rossi 1790; **Location:** country: Italy; stateProvince: Pavia; locality: SIC "Boschi Siro Negri e Moriano" - BN5; verbatimElevation: 62 m; verbatimCoordinates: 32T 502886E 5008393N; verbatimCoordinateSystem: UTM WGS 84; decimalLatitude: 45.229029; decimalLongitude: 9.036770; georeferencedBy: Silvia Stefanelli; georeferenceProtocol: GPS; **Identification:** identifiedBy: Claudio Canepari; dateIdentified: 2011

##### Distribution

Austria, Bosnia and Herzegovina, Bulgaria, Corsica, Croatia, Czech Republic, European Turkey, French mainland, Germany, Greek mainland, Hungary, Italian mainland, Poland, Romania, Russia South, Slovakia, Slovenia, Spanish mainland, Switzerland, Ukraine, Yugoslavia (Fauna Europaea 2013).

##### Notes

The species lives under the bark of various dead broadleaves (Hůrka 2005).

#### Colydium elongatum

(Fabricius, 1787)

Bostrichus elongatum Fabricius, 1787 – Fauna Europaea (2013)

##### Materials

- **Type status:** Other material. **Occurrence:** recordedBy: Silvia Stefanelli; individualCount: 1; lifeStage: adult; **Taxon:** taxonID: urn:lsid:faunaeur.org:taxname:127031; scientificName: Colydium elongatum; order: Coleoptera; family: Zopheridae; genus: Colydium; scientificNameAuthorship: Fabricius 1787; **Location:** country: Italy; stateProvince: Pavia; locality: SIC "Boschi Siro Negri e Moriano" - BN21; verbatimElevation: 66 m; verbatimCoordinates: 32T 506342E 5005026N; verbatimCoordinateSystem: UTM WGS 84; decimalLatitude: 45.198691; decimalLongitude: 9.080746; georeferencedBy: Silvia Stefanelli; georeferenceProtocol: GPS; **Identification:** identifiedBy: Claudio Canepari; dateIdentified: 2011
- **Type status:** Other material. **Occurrence:** recordedBy: Silvia Stefanelli; individualCount: 1; lifeStage: adult; **Taxon:** taxonID: urn:lsid:faunaeur.org:taxname:127031; scientificName: Colydium elongatum; order: Coleoptera; family: Zopheridae; genus: Colydium; scientificNameAuthorship: Fabricius 1787; **Location:** country: Italy; stateProvince: Pavia; locality: SIC "Boschi Siro Negri e Moriano" - BN5; verbatimElevation: 62 m; verbatimCoordinates: 32T 502886E 5008393N; verbatimCoordinateSystem: UTM WGS 84; decimalLatitude: 45.229029; decimalLongitude: 9.036770; georeferencedBy: Silvia Stefanelli; georeferenceProtocol: GPS; **Identification:** identifiedBy: Claudio Canepari; dateIdentified: 2011
- **Type status:** Other material. **Occurrence:** recordedBy: Silvia Stefanelli; individualCount: 5; lifeStage: adult; **Taxon:** taxonID: urn:lsid:faunaeur.org:taxname:127031; scientificName: Colydium elongatum; order: Coleoptera; family: Zopheridae; genus: Colydium; scientificNameAuthorship: Fabricius 1787; **Location:** country: Italy; stateProvince: Pavia; locality: SIC "Boschi Siro Negri e Moriano" - BN10; verbatimElevation: 76 m; verbatimCoordinates: 32T 504479E 5006332N; verbatimCoordinateSystem: UTM WGS 84; decimalLatitude: 45.210461; decimalLongitude: 9.057038; georeferencedBy: Silvia Stefanelli; georeferenceProtocol: GPS; **Identification:** identifiedBy: Claudio Canepari; dateIdentified: 2011
- **Type status:** Other material. **Occurrence:** recordedBy: Silvia Stefanelli; individualCount: 13; lifeStage: adult; **Taxon:** taxonID: urn:lsid:faunaeur.org:taxname:127031; scientificName: Colydium elongatum; order: Coleoptera; family: Zopheridae; genus: Colydium; scientificNameAuthorship: Fabricius 1787; **Location:** country: Italy; stateProvince: Pavia; locality: SIC "Boschi di Vaccarizza" - V2; verbatimElevation: 65 m; verbatimCoordinates: 32T 519868E 4999488N; verbatimCoordinateSystem: UTM WGS 84; decimalLatitude: 45.148589; decimalLongitude: 9.252737; georeferencedBy: Silvia Stefanelli; georeferenceProtocol: GPS; **Identification:** identifiedBy: Claudio Canepari; dateIdentified: 2011

##### Distribution

Austria, Belarus, Belgium, Bosnia and Herzegovina, Britain I., Bulgaria, Corsica, Croatia, Czech Republic, Danish mainland, European Turkey, Finland, French mainland, Germany, Greek mainland, Hungary, Italian mainland, Poland, Romania, Russia East, Russia North, Russia Northwest, Russia South, Sardinia, Slovakia, Slovenia, Spanish mainland, Sweden, Switzerland, Ukraine, Yugoslavia, Near East (Fauna Europaea 2013).

##### Notes

The species is rare. The larva develops under the bark and in the rotting wood of various dead broadleaves and conifer trees. It is a voracious predator of the larvae and pupae of other insects, especially *Platypus* sp. and *Xyloterus* sp. (Alexander 2002, Hůrka 2005).

#### Endophloeus marcovichianus

(Piller & Mitterpacher, 1783)

Silpha marcovichianus (Piller & Mitterpacher, 1783) – Fauna Europaea (2013)

##### Materials

- **Type status:** Other material. **Occurrence:** recordedBy: Silvia Stefanelli; individualCount: 2; lifeStage: adult; **Taxon:** taxonID: urn:lsid:faunaeur.org:taxname:127144; scientificName: Endophloeus marcovichianus; order: Coleoptera; family: Zopheridae; genus: Endophloeus; scientificNameAuthorship: Piller & Mitterpacher 1783; **Location:** country: Italy; stateProvince: Pavia; locality: SIC "Boschi Siro Negri e Moriano" - BN1; verbatimElevation: 68 m; verbatimCoordinates: 32T 503258E 5007870N; verbatimCoordinateSystem: UTM WGS 84; decimalLatitude: 45.224312; decimalLongitude: 9.041499; georeferencedBy: Silvia Stefanelli; georeferenceProtocol: GPS; **Identification:** identifiedBy: Claudio Canepari; dateIdentified: 2011

##### Distribution

Austria, Bosnia and Herzegovina, Bulgaria, Corsica, Croatia, European Turkey, French mainland, Germany, Greek mainland, Hungary, Italian mainland, Macedonia, Poland, Portuguese mainland, Romania, Russia Central, Russia East, Russia North, Russia South, Sardinia, Sicily, Slovakia, Slovenia, Spanish mainland, Switzerland, Ukraine, Yugoslavia, Near East, North Africa (Fauna Europaea 2013).

##### Notes

The species lives mainly in the mountains and occasionally in the lowlands. The larva and adults are found under the loose bark of several broadleaves, especially beech trees (Alexander 2002, Cianferoni et al. 2009).

#### Pycnomerus terebrans

(Oliver, 1790)

##### Materials

- **Type status:** Other material. **Occurrence:** recordedBy: Silvia Stefanelli; individualCount: 7; lifeStage: adult; **Taxon:** taxonID: urn:lsid:faunaeur.org:taxname:127162; scientificName: Pycnomerus terebrans; order: Coleoptera; family: Zopheridae; genus: Pycnomerus; scientificNameAuthorship: Olivier 1790; **Location:** country: Italy; stateProvince: Pavia; locality: SIC "Boschi Siro Negri e Moriano" - BN10; verbatimElevation: 76 m; verbatimCoordinates: 32T 504479E 5006332N; verbatimCoordinateSystem: UTM WGS 84; decimalLatitude: 45.210461; decimalLongitude: 9.057038; georeferencedBy: Silvia Stefanelli; georeferenceProtocol: GPS; **Identification:** identifiedBy: Claudio Canepari; dateIdentified: 2011
- **Type status:** Other material. **Occurrence:** recordedBy: Silvia Stefanelli; individualCount: 1; lifeStage: adult; **Taxon:** taxonID: urn:lsid:faunaeur.org:taxname:127162; scientificName: Pycnomerus terebrans; order: Coleoptera; family: Zopheridae; genus: Pycnomerus; scientificNameAuthorship: Olivier 1790; **Location:** country: Italy; stateProvince: Pavia; locality: SIC "Boschi di Vaccarizza" - V1; verbatimElevation: 62 m; verbatimCoordinates: 32T 519272E 4999526N; verbatimCoordinateSystem: UTM WGS 84; decimalLatitude: 45.148947; decimalLongitude: 9.245157; georeferencedBy: Silvia Stefanelli; georeferenceProtocol: GPS; **Identification:** identifiedBy: Claudio Canepari; dateIdentified: 2011

##### Distribution

Austria, Belgium, Croatia, Czech Republic, European Turkey, French mainland, Germany, Greek mainland, Hungary, Italian mainland, Poland, Portuguese mainland, Romania, Russia East, Russia North, Russia Northwest, Russia South, Sardinia, Sicily, Slovakia, Slovenia, Spanish mainland, Switzerland, Ukraine, Yugoslavia, Near East (Fauna Europaea 2013).

##### Notes

The species is rare and lives in undisturbed broadleaves forests. The larva develops under bark and in old rotting wood (Hůrka 2005).

#### Rhopalocerus rondanii

(Villa & Villa, 1833)

##### Materials

- **Type status:** Other material. **Occurrence:** recordedBy: Silvia Stefanelli; individualCount: 36; lifeStage: adult; **Taxon:** taxonID: urn:lsid:faunaeur.org:taxname:127013; scientificName: Rhopalocerus rondanii; order: Coleoptera; family: Zopheridae; genus: Rhopalocerus; scientificNameAuthorship: Villa & Villa 1833; **Location:** country: Italy; stateProvince: Pavia; locality: SIC "Boschi Siro Negri e Moriano" - BN21; verbatimElevation: 66 m; verbatimCoordinates: 32T 506342E 5005026N; verbatimCoordinateSystem: UTM WGS 84; decimalLatitude: 45.198691; decimalLongitude: 9.080746; georeferencedBy: Silvia Stefanelli; georeferenceProtocol: GPS; **Identification:** identifiedBy: Claudio Canepari; dateIdentified: 2011
- **Type status:** Other material. **Occurrence:** recordedBy: Silvia Stefanelli; individualCount: 1; lifeStage: adult; **Taxon:** taxonID: urn:lsid:faunaeur.org:taxname:127013; scientificName: Rhopalocerus rondanii; order: Coleoptera; family: Zopheridae; genus: Rhopalocerus; scientificNameAuthorship: Villa & Villa 1833; **Location:** country: Italy; stateProvince: Pavia; locality: SIC "Boschi Siro Negri e Moriano" - BN5; verbatimElevation: 62 m; verbatimCoordinates: 32T 502886E 5008393N; verbatimCoordinateSystem: UTM WGS 84; decimalLatitude: 45.229029; decimalLongitude: 9.036770; georeferencedBy: Silvia Stefanelli; georeferenceProtocol: GPS; **Identification:** identifiedBy: Claudio Canepari; dateIdentified: 2011
- **Type status:** Other material. **Occurrence:** recordedBy: Silvia Stefanelli; individualCount: 3; lifeStage: adult; **Taxon:** taxonID: urn:lsid:faunaeur.org:taxname:127013; scientificName: Rhopalocerus rondanii; order: Coleoptera; family: Zopheridae; genus: Rhopalocerus; scientificNameAuthorship: Villa & Villa 1833; **Location:** country: Italy; stateProvince: Pavia; locality: SIC "Boschi Siro Negri e Moriano" - BN10; verbatimElevation: 76 m; verbatimCoordinates: 32T 504479E 5006332N; verbatimCoordinateSystem: UTM WGS 84; decimalLatitude: 45.210461; decimalLongitude: 9.057038; georeferencedBy: Silvia Stefanelli; georeferenceProtocol: GPS; **Identification:** identifiedBy: Claudio Canepari; dateIdentified: 2011

##### Distribution

Bosnia and Herzegovina, Croatia, Czech Republic, French mainland, Germany, Hungary, Italian mainland, Poland, Romania, Russia Central, Russia East, Slovakia, Slovenia, Switzerland, Ukraine, Yugoslavia (Fauna Europaea 2013).

##### Notes

The species lives in isolated and relict forests. The larva develops under the bark of deadwood and in the tree humus often associated with ants of the genus *Lasius*. In one case, the species was found with another beetle *Osmoderma eremita* (Bussler et al. 2005).

#### Synchita humeralis

(Fabricius, 1792)

##### Materials

- **Type status:** Other material. **Occurrence:** recordedBy: Silvia Stefanelli; individualCount: 1; lifeStage: adult; **Taxon:** taxonID: urn:lsid:faunaeur.org:taxname:127099; scientificName: Synchita humeralis; order: Coleoptera; family: Zopheridae; genus: Synchita; scientificNameAuthorship: Fabricius 1792; **Location:** country: Italy; stateProvince: Pavia; locality: SIC "Boschi Siro Negri e Moriano" - BN1; verbatimElevation: 68 m; verbatimCoordinates: 32T 503258E 5007870N; verbatimCoordinateSystem: UTM WGS 84; decimalLatitude: 45.224312; decimalLongitude: 9.041499; georeferencedBy: Silvia Stefanelli; georeferenceProtocol: GPS; **Identification:** identifiedBy: Claudio Canepari; dateIdentified: 2011
- **Type status:** Other material. **Occurrence:** recordedBy: Silvia Stefanelli; individualCount: 1; lifeStage: adult; **Taxon:** taxonID: urn:lsid:faunaeur.org:taxname:127099; scientificName: Synchita humeralis; order: Coleoptera; family: Zopheridae; genus: Synchita; scientificNameAuthorship: Fabricius 1792; **Location:** country: Italy; stateProvince: Pavia; locality: SIC "Boschi Siro Negri e Moriano" - BN10; verbatimElevation: 76 m; verbatimCoordinates: 32T 504479E 5006332N; verbatimCoordinateSystem: UTM WGS 84; decimalLatitude: 45.210461; decimalLongitude: 9.057038; georeferencedBy: Silvia Stefanelli; georeferenceProtocol: GPS; **Identification:** identifiedBy: Claudio Canepari; dateIdentified: 2011
- **Type status:** Other material. **Occurrence:** recordedBy: Silvia Stefanelli; individualCount: 2; lifeStage: adult; **Taxon:** taxonID: urn:lsid:faunaeur.org:taxname:127099; scientificName: Synchita humeralis; order: Coleoptera; family: Zopheridae; genus: Synchita; scientificNameAuthorship: Fabricius 1792; **Location:** country: Italy; stateProvince: Pavia; locality: SIC "Boschi di Vaccarizza" - V1; verbatimElevation: 62 m; verbatimCoordinates: 32T 519272E 4999526N; verbatimCoordinateSystem: UTM WGS 84; decimalLatitude: 45.148947; decimalLongitude: 9.245157; georeferencedBy: Silvia Stefanelli; georeferenceProtocol: GPS; **Identification:** identifiedBy: Claudio Canepari; dateIdentified: 2011

##### Distribution

Belarus, Belgium, Bosnia and Herzegovina, Britain I., Bulgaria, Croatia, Czech Republic, Danish mainland, Estonia, European Turkey, Finland, French mainland, Germany, Greek mainland, Hungary, Italian mainland, Latvia, Lithuania, Macedonia, Moldova Republic of, Norwegian mainland, Poland, Romania, Russia Central, Russia East, Russia North, Russia Northwest, Russia South, Slovakia, Slovenia, Spanish mainland, Sweden, Switzerland, The Netherlands, Ukraine, Yugoslavia (Fauna Europaea 2013).

##### Notes

The species develops on dry branches and under the bark of broadleaves (Hůrka 2005).

## Analysis

Within the 21 families considered, we determined 4,387 individuals belonging to 87 species. Of these species, only 36 were included in the "Atlas of Biodiversity" published from Ticino Valley Regional Park (Furlanetto 2002).

In the SCI "Bosco Siro Negri", we collected 21 families, and we identified 78 saproxylic species (3,310 species), 46 of which had never been reported in the Park (Table 1).

The family with the highest number of species was that of Cerambycidae with 18 different saproxylic species all of which were previously reported in the Atlas of Biodiversity except for *Xylotrechus rusticus* (Linneaus, 1958) which was captured exclusively in the poplar forests. The second largest family was Elateridae with 8 species; 3 of them were not reported in the Park: *Calambus bipustulatus* (Linneaus, 1767), *Cardiophorus anticus* (Erichson, 1840), and *Lacon punctatus* (Herbst, 1779).

The families with only one saproxyilic species were: Cerylonidae with *Cerylon ferrugineum* (Stephens, 1830), Curculionidae with *Phloeophagus lignarius* (Marsham, 1802), Dryophthoridae with *Dryophthorus corticalis* (Paykull, 1792), Eucnemidae with *Melasis buprestoides* (Linneaus, 1761), Lissomidae with *Drapetes mordelloides* (Host, 1789), and Lucanidae with *Dorcus parallelipipedus* (Linneaus, 1785).

Regarding the single species, the most abundant beetle was *Enicmus rugosus* (Herbst, 1793) (Latridiidae) with 708 individuals of which most were captured in the *Populus* managed forests BN5.

In the SCI "Boschi di Vaccarizza", we collected 1.077 individuals belonging to 17 families and 48 species of which 29 species have never been reported in the Park (Table 2). In this SCI, saproxylic beetles belonging to the families of Cerylonidae, Curculionidae, Dryophthoridae, and Lissomidae were not found.

Also in this case, the family with the greatest number of species was Cerambycidae with 10 species already reported within the Park. The second and the third largest families were Elateridae and Tenebrionidae with 5 species each. The families of Bothrideridae, Erotylidae, Laemophloeidae, Lucanidae, and Monotomidae were those with the lowest number of saproxylic species being represented by a single species.

The most abundant species was the Mycetophagidae *Litargus connexus* (Geoffroy, 1785), not reported in the Atlas of Biodiversity in the Park. It is an obligate saproxylic beetle classified as "Least Concern" by the IUCN Red List (Nieto and Alexander 2010) with a total of 236 identified specimens and the highest number of 125 individuals captured in the *Alnus* unmanaged forest (V1).

**Figure 3.**
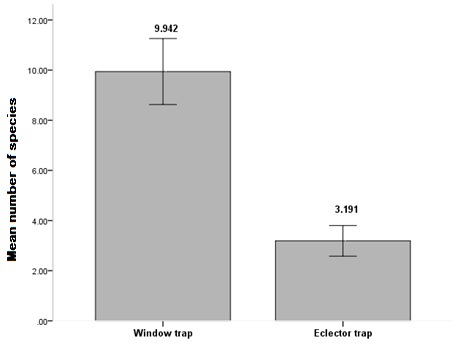
Mean number of saproxylic species captured with Trunk Window Traps and Eclector Traps.

By comparing the two different techniques used for catching saproxylic beetles, we found a significantly high difference in species richness between Window Traps (WT) and Eclector Traps (ET) with a higher number of species captured in the Window Traps (N = 34; WT = 9.942 ± 1.7; ET = 3.191 ± 3.89; Ttest = -9.357, p < 0.05) (Fig. 3).

**Figure 4.**
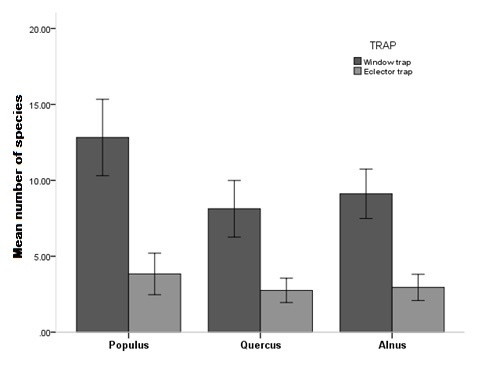
Mean number of saproxylic species captured in the three habitat type with both Trunk Window Traps (dark gray) and Eclector Traps (light gray).

**Figure 5.**
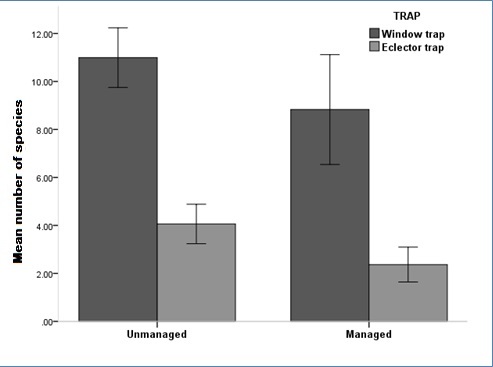
Mean number of saproxylic species captured in the managed and unmanaged forests with both Trunk Windows Traps (dark gray) and Eclector Traps (light gray).

By comparing the species richness among the three habitat types (Table 3, Fig. 4) we observed an high difference for the species collected by the windows traps (One–Way ANOVA F_2,34_ = 5.905; p < 0.01). On the contrary, by considering managed and unmanaged forests instead of habitat types (Table 3, Fig. 5), species richness was significantly higher in unmanaged forests only for those species collected by the eclector traps (T-test: t = 0.371; p < 0.01).

## Discussion

Of the 21 families determined, 7 were not included in the previous checklist published in the "Atlas of Biodiversity" (Furlanetto 2002): Cerylonidae, Dryophthoridae, Eucnemidae, Laemophloeidae, Lissomidae, Monotomidae, and Zopheridae. Among these families, we found interesting species such as the Eucnemidae *Melasis buprestoides* which is an obligate saproxyilic beetle that develops in broadleaved forests both in the plains and in the mountains (Hůrka 2005) and is reported as Least Concern in the IUCN Red List (IUCN Red List, 2010). Another interesting species is the Anthribidae *Eusphyrus vasconicus* which was caught in the *Alnus* forests of the SCI "Boschi di Vaccarizza" and is rare and has been only recently reported in Italy (Tryzna and Valentine 2011, Cornacchia and Colonelli 2012).

Also, the Elateridae *Calambus bipustulatus* is rare for Italy as well as the 4 *Ampedus* species that are all classified as Least Concern in the IUCN Red List (IUCN Red List, 2010) and are closely associated with ancient and mature forests (Platia pers. comm.).

It is important to note the presence of other species included as Least Concern in the Red List of the IUCN and never previously found in the Park: the Cerambycidae *Xylotrechus rusticus*, the Cetoniidae *Valgus hemipterus*, the Elateridae *Lacon punctatus*, the Mycetophagidae *Mycetophagus piceus*, and the most abundant species of our study area, *Litargus connexus*.

Finally, it is interesting to underline the presence of two invasive species captured during the study: Cerambycidae *Neoclytus acuminatus* (Fabricius, 1775) and *Xylotrechus stebbingi* (Gahan, 1906). The latter was recently introduced into Italy but now is widely spread throughout Northern and Central Italy.

The period of field collection was planned in detail to provide a long and exclusive season of work to fully cover the reproductive cycle of a large number of beetles.

The combined use of two different types of traps significantly expanded the spectrum of insects capturable. The Eclector Trap caught a lower number of individuals and beetle species compared to Window Traps as is also described by other authors (Okland 1996, Bakke 1999, Schiegg 2000, Ranius and Jansson 2002, Wikars et al. 2005). However, this trapping method is more efficient in catching truly saproxyilic beetles (Alinvi et al. 2007), and the species were collected exactly where they developed.

The Trunk Window Trap captured a large number of insects also coming from the neighboring plots and/or forests. In this case, the site specific differences (i.e: amount of deadwood) disappeared and the differences became more evident at a landscape level such as habitat type.

Very few saproxylic species are listed in the EU Habitat Directive, but there are many others that should be considered in conservation plans for which we haven’t had sufficient information about. The lack of knowledge on the ecology and distribution of these species make it difficult to establish criteria for their protection. Although we didn’t find species listed in the Annexes of the EU Habitat Directive, some of the species found are locally threatened anyway because of their rarity, local distribution, and strong linkage to old forests. Among these species there are the Bothrideridae *Bothrideres bipunctatus*, the Cerambycidae *Prionus coriarius* and *Xylotrechus rusticus*, the Dryophthoridae *Dryophthorus corticalis*, the Eucnemidae *Nematodes filum* (with only 1 individual captured in *Alnus* unmanged forest), the Histeridae *Aeletes atomarius* and *Paromalus flavicornis*, the Laemophloeidae *Cryptolestes duplicatus*,, the Latridiidae *Enicmus rugosus* and *Latridius hirtus*, the Mycetophagidae *Mycetophagus piceus*, and the Zopheridae *Colydium elongatum* and *Pycnomerus terebrans*.

Thus, studies like this are important for increasing the ecological knowledge of forest beetle species and can provide a starting point for implementing management and conservation actions.

## Supplementary Material

XML Treatment for Anthribus nebulosus

XML Treatment for Eusphyrus vasconicus

XML Treatment for Phaenotherion fasciculatum

XML Treatment for Platystomos albinus

XML Treatment for Bothrideres bipunctatus

XML Treatment for Oxylaemus cylindricus

XML Treatment for Aegomorphus clavipes

XML Treatment for Aegosoma scabricorne

XML Treatment for Cerambyx scopolii

XML Treatment for Chlorophorus varius

XML Treatment for Clytus arietis

XML Treatment for Grammoptera ruficornis

XML Treatment for Leiopus nebulosus

XML Treatment for Leptura aurulenta

XML Treatment for Mesosa nebulosa

XML Treatment for Morimus asper

XML Treatment for Neoclytus acuminatus

XML Treatment for Phymatodes testaceus

XML Treatment for Pogonocherus hispidus

XML Treatment for Prionus (Prionus) coriarius

XML Treatment for Pseudovadonia livida

XML Treatment for Stenurella melanura

XML Treatment for Stictoleptura cordigera

XML Treatment for Strangalia attenuata

XML Treatment for Tetrops praeustus

XML Treatment for Xylotrechus antilope

XML Treatment for Xylotrechus rusticus

XML Treatment for Xylotrechus stebbingi

XML Treatment for Cerylon ferrugineum

XML Treatment for Cetonia aurata

XML Treatment for Oxythyrea funesta

XML Treatment for Potosia cuprea

XML Treatment for Tropinota (Epicometis) hirta

XML Treatment for Valgus hemipterus

XML Treatment for Phloeophagus lignarius

XML Treatment for Dryophthorus corticalis

XML Treatment for Ampedus (Ampedus) cinnaberinus

XML Treatment for Ampedus (Ampedus) pomonae

XML Treatment for Ampedus (Ampedus) pomorum

XML Treatment for Ampedus (Ampedus) sanguinolentus

XML Treatment for Calambus bipustulatus

XML Treatment for Cardiophorus (Cardiophorus) anticus

XML Treatment for Lacon punctatus

XML Treatment for Melanotus (Melanotus) villosus

XML Treatment for Dacne (Dacne) bipustulata

XML Treatment for Tritoma bipustulata

XML Treatment for Melasis buprestoides

XML Treatment for Nematodes filum

XML Treatment for Aeletes (Aeletes) atomarius

XML Treatment for Gnathoncus rotundatus

XML Treatment for Hololepta (Hololepta) plana

XML Treatment for Paromalus (Paromalus) flavicornis

XML Treatment for Platylomalus complanatus

XML Treatment for Cryptolestes duplicatus

XML Treatment for Laemophloeus monilis

XML Treatment for Placonotus testaceus

XML Treatment for Enicmus rugosus

XML Treatment for Latridius hirtus

XML Treatment for Drapetes mordelloides

XML Treatment for Dorcus parallelipipedus

XML Treatment for Monotoma (Monotoma) longicollis

XML Treatment for Rhizophagus (Rhizophagus) bipustulatus

XML Treatment for Litargus (Litargus) connexus

XML Treatment for Mycetophagus (Ulolendus) piceus

XML Treatment for Mycetophagus (Mycetophagus) quadripustulatus

XML Treatment for Cryptarcha strigata

XML Treatment for Epuraea aestiva

XML Treatment for Epuraea guttata

XML Treatment for Epuraea marseuli

XML Treatment for Silvanus bidentatus

XML Treatment for Silvanus unidentatus

XML Treatment for Uleiota planata

XML Treatment for Diaperis boleti

XML Treatment for Hypophloeus bicolor

XML Treatment for Hypophloeus fasciatus

XML Treatment for Hypophloeus unicolor

XML Treatment for Platydema violaceum

XML Treatment for Scaphidema metallicum

XML Treatment for Stenomax aeneus

XML Treatment for Uloma culinaris

XML Treatment for Bitoma crenata

XML Treatment for Colobicus hirtus

XML Treatment for Colydium elongatum

XML Treatment for Endophloeus marcovichianus

XML Treatment for Pycnomerus terebrans

XML Treatment for Rhopalocerus rondanii

XML Treatment for Synchita humeralis

## Figures and Tables

**Table 1.** Saproxylic beetle species collected in the SCI “Boschi Siro Negri e Moriano” (number of specimens per taxon).

FAMILY	POPULUS FOREST MANAGED BN1	POPULUS FOREST UNMANAGED BN21	OAK FOREST MANAGED BN5	OAK FOREST UNMANAGED BN10	TOTAL	ALREADY REPORTED IN THE PARK
** ANTHRIBIDAE **	1	0	2	5	8	
*Anthribus nebulosus*	0	0	1	0	1	X
*Phaenotherion fasciculatum*	0	0	0	2	2	X
*Platystomos albinus*	1	0	1	3	5	
** BOTHRIDERIDAE **	11	2	6	15	34	
*Bothrideres bipunctatus*	11	2	4	6	23	
*Oxylaemus cylindricus*	0	0	2	9	11	
** CERAMBYCIDAE **	47	16	23	19	105	
*Aegomorphus clavipes*	0	5	0	0	5	X
*Aegosoma scabricorne*	7	1	3	7	18	X
*Chlorophorus varius*	3	1	0	0	4	X
*Clytus arietis*	1	0	0	1	2	X
*Grammoptera ruficornis*	0	0	1	0	1	X
*Leiopus nebulosus*	3	0	1	1	5	X
*Mesosa nebulosa*	0	0	1	0	1	X
*Neoclytus acuminatus*	0	3	1	0	4	X
*Phymatodes testaceus*	1	0	0	1	2	X
*Pogonocherus hispidus*	2	0	4	0	6	X
*Prionus coriarius*	1	0	3	4	8	X
*Pseudovadonia livida*	2	0	2	3	7	X
*Stenurella melanura*	3	1	4	1	9	X
*Strangalia attenuata*	4	0	0	0	4	X
*Tetrops praeustus*	0	0	1	0	1	X
*Xylotrechus antilope*	0	0	1	0	1	X
*Xylotrechus rusticus*	19	2	0	0	21	
*Xylotrechus stebbingi*	1	3	1	1	6	X
** CERYLONIDAE **	3	2	4	0	9	
*Cerylon ferrugineum*	3	2	4	0	9	
** CETONIIDAE **	95	116	31	32	274	
*Cetonia aurata*	13	2	5	2	22	
*Oxythyrea funesta*	16	2	4	0	22	
*Potosia cuprea*	1	3	0	0	4	
*Tropinota hirta*	1	1	0	0	2	
*Valgus hemipterus*	64	108	22	30	224	
** CURCULIONIDAE **	0	1	0	1	2	
*Phloeophagus lignarius*	0	1	0	1	2	
** DRYOPHTHORIDAE **	0	2	1	28	31	
*Dryophthorus corticalis*	0	2	1	28	31	
** ELATERIDAE **	18	0	6	22	46	
*Ampedus cinnaberinus*	0	0	2	0	2	X
*Ampedus pomonae*	2	0	0	0	2	X
*Ampedus pomorum*	3	0	0	3	6	X
*Ampedus sanguinolentus*	11	0	1	0	12	X
*Calambus bipustulatus*	0	0	0	1	1	
*Cardiophorus anticus*	2	0	1	3	6	
*Lacon punctatus*	0	0	2	2	4	
*Melanotus villosus*	0	0	0	13	13	X
** EROTYLIDAE **	10	23	45	4	82	
*Dacne bipustulata*	9	23	39	4	75	
*Tritoma bipustulata*	1	0	6	0	7	X
** EUCNEMIDAE **	1	0	2	4	7	
*Melasis buprestoides*	1	0	2	4	7	
** HISTERIDAE **	12	40	25	60	137	
*Aeletes atomarius*	2	10	2	2	16	
*Gnathoncus rotundatus*	1	1	0	0	2	
*Hololepta plana*	0	5	0	0	5	X
*Paromalus flavicornis*	9	24	23	58	114	
** LAEMOPHLOEIDAE **	22	16	206	15	259	
*Cryptolestes duplicatus*	0	0	1	5	6	
*Laemophloeus monilis*	1	0	0	0	1	
*Placonotus testaceus*	21	16	205	10	252	
** LATRIDIIDAE **	70	79	526	65	740	
*Enicmus rugosus*	67	57	524	60	708	
*Latridius hirtus*	3	22	2	5	32	
** LISSOMIDAE **	1	5	0	0	6	
*Drapetes mordelloides*	1	5	0	0	6	
** LUCANIDAE **	49	11	7	38	105	
*Dorcus parallelipipedus*	49	11	7	38	105	X
** MONOTOMIDAE **	34	99	50	315	498	
*Monotoma longicollis*	1	1	0	0	2	
*Rhizophagus bipustulatus*	33	98	50	315	496	
** MYCETOPHAGIDAE **	127	121	122	217	587	
*Litargus connexus*	101	91	115	211	518	
*Mycetophagus piceus*	4	3	1	0	8	
*Mycetophagus quadripustulatus*	22	27	6	6	61	X
** NITIDULIDAE **	13	6	42	53	114	
*Cryptarcha strigata*	0	0	2	0	2	
*Epuraea aestiva*	1	0	1	1	3	
*Epuraea guttata*	12	6	39	52	109	
** SILVANIDAE **	40	56	22	21	139	
*Silvanus bidentatus*	2	3	6	4	15	
*Silvanus unidentatus*	28	37	5	4	74	
*Uleiota planatus*	10	16	11	13	50	X
** TENEBRIONIDAE **	11	25	18	12	66	
*Diaperis boleti*	1	0	0	1	2	
*Hypophloeus bicolor*	0	2	0	0	2	
*Hypophloeus fasciatus*	0	0	0	3	3	
*Platydema violaceum*	0	1	0	0	1	
*Scaphidema metallicum*	0	0	4	0	4	
*Stenomax aeneus*	0	21	0	3	24	X
*Uloma culinaris*	10	1	14	5	30	X
** ZOPHERIDAE **	14	58	4	16	92	
*Bitoma crenata*	11	20	1	0	32	X
*Colobicus hirtus*	0	1	1	0	2	
*Colydium elongatum*	0	1	1	5	7	
*Endophloeus marcovichianus*	2	0	0	0	2	
*Pycnomerus terebrans*	0	0	0	7	7	
*Rhopalocerus rondanii*	0	36	1	3	40	
*Synchita humeralis*	1	0	0	1	2	
**TOTAL**	579	676	1141	914	3310	

**Table 2.** Saproxylic beetle species collected in the SCI “Boschi di Vaccarizza” (number of specimens per taxon).

FAMILY	ALDER FOREST UNMANAGED V1	ALDER FOREST MANAGED V2	TOTAL	ALREADY REPORTED IN THE PARK
** ANTHRIBIDAE **	15	7	22	
*Eusphyrus vasconicus*	12	5	17	
*Platystomos albinus*	3	2	5	
** BOTHRIDERIDAE **	3	2	5	
*Bothrideres bipunctatus*	3	2	5	
** CERAMBYCIDAE **	13	10	23	
*Aegosoma scabricorne*	3	4	7	X
*Cerambyx scopolii*	0	1	1	X
*Grammoptera ruficornis*	0	2	2	X
*Leiopus nebulosus*	0	3	3	X
*Leptura aurulenta*	1	0	1	X
*Morimus asper*	4	0	4	X
*Pogonocherus hispidus*	2	0	2	X
*Stenurella melanura*	1	0	1	X
*Stictoleptura cordigera*	1	0	1	X
*Xylotrechus stebbingi*	1	0	1	X
** CETONIIDAE **	23	18	41	
*Cetonia aurata*	1	3	4	
*Valgus hemipterus*	22	15	37	
** ELATERIDAE **	10	6	16	
*Ampedus pomonae*	1	0	1	X
*Ampedus pomorum*	2	2	4	X
*Ampedus sanguinolentus*	0	1	1	X
*Cardiophorus anticus*	3	0	3	
*Lacon punctatus*	4	3	7	
** EROTYLIDAE **	1	1	2	
*Dacne bipustulata*	1	1	2	
** EUCNEMIDAE **	2	2	4	
*Melasis buprestoides*	1	2	3	
*Nematodes filum*	1	0	1	
** HISTERIDAE **	66	97	163	
*Aeletes atomarius*	1	0	1	
*Paromalus flavicornis*	58	93	151	
*Platylomalus complanatus*	7	4	11	
** LAEMOPHLOEIDAE **	20	21	41	
*Placonotus testaceus*	20	21	41	
** LATRIDIIDAE **	51	63	114	
*Enicmus rugosus*	43	57	100	
*Latridius hirtus*	8	6	14	
** LUCANIDAE **	77	60	137	
*Dorcus parallelipipedus*	77	60	137	X
** MONOTOMIDAE **	52	48	100	
*Rhizophagus bipustulatus*	52	48	100	
** MYCETOPHAGIDAE **	127	111	238	
*Litargus connexus*	125	111	236	
*Mycetophagus quadripustulatus*	2	0	2	X
** NITIDULIDAE **	7	5	12	
*Epuraea aestiva*	1	0	1	
*Epuraea guttata*	3	5	8	
*Epuraea marseuli*	3	0	3	
** SILVANIDAE **	55	11	66	
*Silvanus bidentatus*	5	2	7	
*Silvanus unidentatus*	27	5	32	
*Uleiota planatus*	23	4	27	X
** TENEBRIONIDAE **	39	38	77	
*Hypophloeus bicolor*	0	3	3	
*Hypophloeus unicolor*	3	26	29	
*Scaphidema metallicum*	2	4	6	
*Stenomax aeneus*	16	2	18	X
*Uloma culinaris*	18	3	21	X
** ZOPHERIDAE **	3	13	16	
*Colydium elongatum*	0	13	13	
*Pycnomerus terebrans*	1	0	1	
*Synchita humeralis*	2	0	2	
**TOTAL**	564	513	1077	

**Table 3.** Difference in species richness among the three habitat type and between managed and unmanaged forests. Table shows the mean number of saproxylic species collected using both Eclector and Trunk Windows Traps. (** p < 0.01)

	Populus	Quercus	Alnus	F _ANOVA_	Unmanaged	Managed	F _T.TEST_
Window Traps	12.8 ± 4.1	8.1 ± 3.2	9.1 ± 2.8	5.905**	10.9 ± 2.6	8.8 ± 4.7	2.051
Eclector Traps	3.8 ± 2.3	2.7 ± 1.32	2.9 ± 1.4	1.205	4.0 ±1.6	2.3 ± 1.5	0.371**
